# Foodborne Illness Acquired in the United States—Major Pathogens

**DOI:** 10.3201/eid1701.P11101

**Published:** 2011-01

**Authors:** Elaine Scallan, Robert M. Hoekstra, Frederick J. Angulo, Robert V. Tauxe, Marc-Alain Widdowson, Sharon L. Roy, Jeffery L. Jones, Patricia M. Griffin

**Affiliations:** Author affiliation: Centers for Disease Control and Prevention, Atlanta, Georgia, USA

**Keywords:** Food poisoning, gastroenteritis, diarrhea, population surveillance, incidence estimates, norovirus, viruses, bacteria, United States, research

## Abstract

Estimates of foodborne illness can be used to direct food safety policy and interventions. We used data from active and passive surveillance and other sources to estimate that each year 31 major pathogens acquired in the United States caused 9.4 million episodes of foodborne illness (90% credible interval [CrI] 6.6–12.7 million), 55,961 hospitalizations (90% CrI 39,534–75,741), and 1,351 deaths (90% CrI 712–2,268). Most (58%) illnesses were caused by norovirus, followed by nontyphoidal *Salmonella* spp. (11%), *Clostridium perfringens* (10%), and *Campylobacter* spp. (9%). Leading causes of hospitalization were nontyphoidal *Salmonella* spp. (35%), norovirus (26%), *Campylobacter* spp. (15%), and *Toxoplasma* *gondii* (8%). Leading causes of death were nontyphoidal *Salmonella* spp. (28%), *T.* *gondii* (24%), *Listeria* *monocytogenes* (19%), and norovirus (11%). These estimates cannot be compared with prior (1999) estimates to assess trends because different methods were used. Additional data and more refined methods can improve future estimates.

## Abstract 2

Each year, 31 pathogens caused 9.4 million episodes of foodborne illness, resulting in 55,961 hospitalizations and 1,351 deaths.

Estimates of the overall number of episodes of foodborne illness are helpful for allocating resources and prioritizing interventions. However, arriving at these estimates is challenging because food may become contaminated by many agents (e.g., a variety of bacteria, viruses, parasites, and chemicals), transmission can occur by nonfood mechanisms (e.g., contact with animals or consumption of contaminated water), the proportion of disease transmitted by food differs by pathogen and by host factors (e.g. age and immunity), and only a small proportion of illnesses are confirmed by laboratory testing and reported to public health agencies.

Laboratory-based surveillance provides crucial information for assessing foodborne disease trends. However, because only a small proportion of illnesses are diagnosed and reported, periodic assessments of total episodes of illness are also needed. (Hereafter, episodes of illness are referred to as illnesses.) Several countries have conducted prospective population-based or cross-sectional studies to supplement surveillance and estimate the overall number of foodborne illnesses (*1*). In 2007, the World Health Organization launched an initiative to estimate the global burden of foodborne diseases (*2*).

In 1999, the Centers for Disease Control and Prevention provided comprehensive estimates of foodborne illnesses, hospitalizations, and deaths in the United States caused by known and unknown agents (*3*). This effort identified many data gaps and methodologic limitations. Since then, new data and methods have become available. This article is 1 of 2 reporting new estimates of foodborne diseases acquired in the United States (hereafter referred to as domestically acquired). This article provides estimates of major known pathogens; the other provides estimates for agents of acute gastroenteritis not specified in this article (*4*).

## Methods

Adequate data for preparing national estimates were available for 31 pathogens. We estimated the number of foodborne illnesses, hospitalizations, and deaths caused by these 31 domestically acquired pathogens by using data shown in Table A1 and Technical Appendix 1.

Data were mostly from 2000–2008, and all estimates were based on the US population in 2006 (299 million persons). Estimates were derived from statistical models with many inputs, each with some measure of uncertainty (*5*). To reflect this uncertainty, we used probability distributions to describe a range of plausible values for all model inputs. We expressed model outputs as probability distributions summarized by a mean point estimate with 90% credible intervals (CrIs). We used 2 types of modeling approaches for different types of data: 1) models that began with counts of laboratory-confirmed illnesses and were adjusted for undercounts (because of underreporting and underdiagnosis) and thus scaled up to the estimated number of illnesses and 2) models that began with a US population and used incidence data to scale down to the estimated number of illnesses (Table 1). The modeling approaches used and parameters of these probability distributions are detailed in Technical Appendix 2 and Technical Appendix 3; the proportions cited are modal values.

**Table 1:** Modeling approaches used to estimate the total number of illnesses for different types of data, United States*

Pathogens for which laboratory-confirmed illnesses were scaled up			Pathogens for which US population was scaled down
Active surveillance data	Passive surveillance data	Outbreak surveillance data	
*Campylobacter s*pp.	*Brucella* spp.	*Bacillus cereus*	Astrovirus
*Cryptosporidium* spp.	*Clostridium botulinum*	*Clostridium perfringens*	Norovirus
*Cyclospora cayetanensis*	*Giardia intestinalis*	ETEC†	Rotavirus
STEC O157	Hepatitis A virus	*Staphylococcus aureus*	Sapovirus
STEC non-O157	*Mycobacterium bovis*	*Streptococcus* spp. group A	*Toxoplasma gondii*
*Listeria monocytogenes*	*Trichinella* spp.		
*Salmonella* spp., nontyphoidal‡	*Vibrio cholerae*, toxigenic		
*S. enterica* serotype Typhi	*Vibrio parahaemolyticus*		
*Shigella* spp.	*Vibrio vulnificus*		
*Yersinia enterocolitica*	*Vibrio* spp., other		

*ETEC, enterotoxigenic *Escherichi coli*; STEC, Shiga toxin–producing *E. coli.* †Numbers of *E. coli* other than STEC or ETEC assumed to be same as for ETEC. ‡Includes all serotypes other than Typhi.

### Illnesses

Laboratory-based surveillance data were available for 25 pathogens (Table A1). The following events must occur for an illness to be ascertained and included in laboratory-based surveillance: the ill person must seek medical care, a specimen must be submitted for laboratory testing, the laboratory must test for and identify the causative agent, and the illness must be reported to public health authorities. If a break occurs in any of the first 3 steps of this surveillance chain, the causative agent will not be laboratory confirmed (underdiagnosis). Furthermore, although all laboratory-confirmed illnesses are reported by active surveillance, some will not be reported by passive surveillance (underreporting). Therefore, to estimate the number of illnesses caused by pathogens under public health surveillance, we determined the number of laboratory-confirmed illnesses and adjusted for underdiagnosis and, if necessary, for underreporting by using a series of component multipliers.

Laboratory-confirmed illnesses for these 25 pathogens were reported through 5 surveillance programs: the Foodborne Diseases Active Surveillance Network (FoodNet) for *Campylobacter* spp., *Cryptosporidium* spp., *Cyclospora cayetanensis*, Shiga toxin–producing *Escherichia coli* (STEC) O157, STEC non-O157, *Listeria monocytogenes*, nontyphoidal *Salmonella* spp., *Salmonella enterica* serotype Typhi, *Shigella* spp., and *Yersinia enterocolitica*; the National Notifiable Diseases Surveillance System (NNDSS) for *Brucella* spp., *Clostridium botulinum*, *Trichinella* spp., hepatitis A virus, and *Giardia intestinalis*; the Cholera and Other *Vibrio* Illness Surveillance (COVIS) system for toxigenic *Vibrio cholerae*, *V. vulnificus*, *V. parahemolyticus*, and other *Vibrio* spp.; the National Tuberculosis Surveillance System (NTSS) for *Mycobacterium bovis*; and the Foodborne Disease Outbreak Surveillance System (FDOSS) for *Bacillus cereus, Clostridium perfringens,* enterotoxigenic *E. coli* (ETEC), *Staphylococcus aureu*s, and *Streptococcus* spp. group A (Table A1; Technical Appendix 1). When data were available from >1 surveillance system, we used active surveillance data from FoodNet, except for *Vibrio* spp., for which we used COVIS because of geographic clustering of *Vibrio* spp. infections outside FoodNet sites. We used data on outbreak-associated illnesses from FDOSS only for pathogens for which no data were available from other systems.

Because FoodNet conducts surveillance at 10 sites (*6*), we estimated the number of laboratory-confirmed illnesses in the United States by applying incidence from FoodNet to the estimated US population for 2006 (*7*). We constructed a probability distribution based on extrapolation of rates by year (2005–2008) in each FoodNet site (Technical Appendix 3). We used data from 2005–2008 because the FoodNet surveillance area was constant during that period and because FoodNet began collecting information on foreign travel in 2004. We used data from 2000–2007 for NNDSS, COVIS, and FDOSS and annual counts of reported illnesses for our probability distributions. Some evidence of trend was found for illness caused by hepatitis A virus, *S. aureus,* and *Vibrio* spp.; therefore, recent years were weighted more heavily (Technical Appendix 2, Technical Appendix 3). NTSS was used to determine the number of reported illnesses caused by *M. bovis* during 2004–2007.

We assumed that all laboratory-confirmed illnesses were reported to FoodNet active surveillance in the relevant catchment areas. Because COVIS and NNDSS conduct passive surveillance, we applied an underreporting multiplier (1.1 for bacteria and 1.3 for parasites) derived by comparing incidence of all nationally notifiable illnesses ascertained through FoodNet with that reported to NNDSS (Technical Appendix 4) For the 5 bacteria for which only outbreak data were available, we estimated the number of laboratory-confirmed illnesses by creating an underreporting multiplier as follows. We determined the proportion of illnesses ascertained through FoodNet that were caused by *Campylobacter* spp.*, Cryptosporidium* spp., *C. cayatanensis*, *L. monocytogenes*, *Salmonella* spp., *Shigella* spp., STEC, *Vibrio* spp., and *Y.* *enterocolitica* that were also reported to FDOSS as outbreak associated and applied the inverse of this proportion, 25.5, to those pathogens (Technical Appendix 4). We assumed that all illnesses caused by *M. bovis* were reported to NTSS.

To adjust for underdiagnosis resulting from variations in medical care seeking, specimen submission, laboratory testing, and test sensitivity, we created pathogen-specific multipliers. To adjust for medical care seeking and specimen submission, we pooled data from FoodNet Population Surveys in 2000–2001, 2002–2003 (*8*), and 2006–2007 (Centers for Disease Control and Prevention, unpub. data) from which we estimated the proportion of persons who in the past month reported an acute diarrheal illness (>3 loose stools in 24 hours lasting >1 day or resulting in restricted daily activities) and sought medical care and submitted a stool sample for that illness. Because persons with more severe illness are more likely to seek care (*9*), we estimated pathogen-specific proportions of persons with laboratory-confirmed infections who had severe illness (e.g., bloody diarrhea) and used medical care seeking and stool sample submission rates for bloody (35% and 36%, respectively) and nonbloody (18% and 19%, respectively) diarrhea as surrogates for severe and mild cases of most illnesses (Technical Appendix 3). However, for infections with *L. monocytogenes*, *M. bovis*, and *V. vulnificus* and severe infections with hepatitis A virus, we assumed high rates of medical care seeking (i.e., we assumed that 100% of persons with *M. bovis* infection and 90% with *L. monocytogenes*, *V. vulnificus*, or severe hepatitis A virus infections sought care) and specimen submission (100% for hepatitis A virus and *M. bovis*, 80% for others). We accounted for percentage of laboratories that routinely tested for specific pathogens (25%–100%) and test sensitivity (28%–100%) by using data from FoodNet (*10**,**11*) and other surveys of clinical diagnostic laboratory practices ( Technical Appendix 3). For the 5 pathogens for which data were from outbreaks only, we used the nontyphoidal *Salmonella* spp. underdiagnosis multiplier.

Alternative approaches were used for infections not routinely reported by any surveillance system (i.e., diarrheagenic *E. coli* other than STEC and ETEC, *T. gondii*, astrovirus, rotavirus, sapovirus, and norovirus) (Technical Appendix 1, Technical Appendix 2, Technical Appendix 3). We assumed diarrheagenic *E. coli* other than STEC and ETEC to be as common as ETEC. Illnesses caused by *T. gondii* were estimated by using nationally representative serologic data from the 1999–2004 National Health and Nutrition Examination Survey (*12*) and an estimate that clinical illness develops in 15% of persons who seroconvert (*13*). We assumed that 75% of children experience an episode of clinical rotavirus illness by 5 years of age, consistent with findings from other studies (*14*), and used this estimate for astrovirus and sapovirus. We estimated norovirus illnesses by applying mean proportion of all acute gastroenteritis caused by norovirus (11%) according to studies in other industrialized countries (*15**–**18*) to estimates of acute gastroenteritis from FoodNet Population Surveys ( Table A1; Technical Appendix 1, Technical Appendix 2, Technical Appendix 3) (*4*).

### Hospitalizations and Deaths

For most pathogens, numbers of hospitalizations and deaths were estimated by determining (from surveillance data) the proportion of persons who were hospitalized and the proportion who died and applying these proportions to the estimated number of laboratory-confirmed illnesses (Table A1; Technical Appendix 1, Technical Appendix 3). Rates of hospitalization and death caused by *G.* *intestinalis* and *T. gondii* were based on the 2000–2006 Nationwide Inpatient Sample. Because some persons with illnesses that were not laboratory confirmed would also have been hospitalized and died, we doubled the number of hospitalizations and deaths to adjust for underdiagnosis, similar to the method used by Mead et al. (*3*) but applied an uncertainty distribution (Technical Appendix 3). For diarrheagenic *E. coli* other than STEC and ETEC, total numbers of hospitalizations and deaths were assumed to be the same as those for ETEC. For rotavirus, we used previous estimates (*14*). For astrovirus and sapovirus, we assumed that the number was 25% that of rotavirus (*19**,**20*). Numbers of norovirus hospitalizations and deaths were determined by multiplying the estimated number of hospitalizations and deaths caused by acute gastroenteritis, estimated by using national data on outpatient visits resulting in hospitalization, hospital discharge surveys, and death certificates (Table A1; Technical Appendix 1, Technical Appendix 2, Technical Appendix 3) (*4*), by the same norovirus proportion (11%) used to estimate illnesses (*15**–**18*).

### Domestically Acquired Foodborne Illnesses

Data from published studies and surveillance were used to determine, for each pathogen, the proportion of illnesses acquired while the person had been traveling outside the United States (Technical Appendix 1, Technical Appendix 2, Technical Appendix 3). The remaining proportion was considered domestically acquired. We based our estimates of the proportion of domestically acquired foodborne illnesses caused by each pathogen on data from surveillance, risk factor studies, and a literature review (Technical Appendix 1, Technical Appendix 2, Technical Appendix 3).

### Uncertainty Analysis

We used empirical data, when available, to define entire distributions or parameters of distributions (Technical Appendix 3). When data were sparse, we made reasoned judgments based on context, plausibility, and previously published estimates. The parametric distribution used for almost all multipliers was a 4-parameter beta (modified PERT) distribution (*21*). The first 3 parameters are low, modal, and high. The fourth parameter is related to the variability of the distribution. We typically fixed this last parameter at 4, which yields the simple PERT distribution (*21*). However, when describing the outbreak reporting multiplier, we used a value of 20 (Technical Appendix 44).

Uncertainty in the estimates is the cumulative effect of uncertainty of each of the model inputs. We iteratively generated sets of independent pathogen-specific adjustment factors and used these multipliers to estimate illnesses, hospitalizations, and deaths (Figure; Technical Appendix 2). On the basis of 100,000 iterations, we obtained empirical distributions of counts corresponding to Bayesian posterior distributions and used these posterior distributions to generate a point estimate (posterior mean) and upper and lower 5% limits for 90% CrIs. Because incidence of illnesses differed by location and over time, we included these variations in the models, which led to wider CrIs than if we had assumed that inputs represented independent random samples of a fixed US population. We used SAS version 9.2 (SAS Institute, Cary, NC, USA) for these analyses.

## Results

### Foodborne Illnesses

We estimate that each year in the United States, 31 pathogens caused 37.2 million (90% CrI 28.4–47.6 million) illnesses, of which 36.4 million (90% CrI 27.7–46.7 million) were domestically acquired; of these, 9.4 million (90% CrI 6.6–12.7 million) were foodborne (Table 2). We estimate that 5.5 million (59%) foodborne illnesses were caused by viruses, 3.6 million (39%) by bacteria, and 0.2 million (2%) by parasites. The pathogens that caused the most illnesses were norovirus (5.5 million, 58%), nontyphoidal *Salmonella* spp. (1.0 million, 11%), *C. perfringens* (1.0 million, 10%), and *Campylobacter* spp. (0.8 million, 9%).

**Table 2:** Estimated annual number of episodes of domestically acquired foodborne illnesses caused by 31 pathogens, United States*

Pathogen	Laboratory confirmed	Multipliers		Travel related, %	Foodborne, %†	Domestically acquired foodborne, mean (90% credible interval)
		Under-reporting	Under-diagnosis			
Bacteria						
*Bacillus cereus,* foodborne	85‡	25.5	29.3	<1	100	63,400 (15,719–147,354)
*Brucella* spp.	120§	1.1	15.2	16	50	839 (533–1,262)
*Campylobacter* spp.	43,696¶	1.0	30.3	20	80	845,024 (337,031–1,611,083)
*Clostridium botulinum,* foodborne	25§	1.1	2.0	<1	100	55 (34–91)
*Clostridium perfringens,* foodborne	1,295‡	25.5	29.3	<1	100	965,958 (192,316–2,483,309)
STEC O157	3,704¶	1.0	26.1	4	68	63,153 (17,587–149,631)
STEC non-O157	1,579¶	1.0	106.8	18	82	112,752 (11,467–287,321)
ETEC, foodborne	53‡	25.5	29.3	55	100	17,894 (24–46,212)
Diarrheagenic *E. coli* other than STEC and ETEC	53	25.5	29.3	<1	30	11,982 (16–30,913)
* Listeria monocytogenes*	808¶	1.0	2.1	3	99	1,591 (557–3,161)
* Mycobacterium bovis*	195¶	1.0	1.1	70	95	60 (46–74)
*Salmonella* spp., nontyphoidal	41,930¶	1.0	29.3	11	94	1,027,561 (644,786–1,679,667)
*S. enterica* serotype Typhi	433¶	1.0	13.3	67	96	1,821 (87–5,522)
*Shigella* spp.	14,864¶	1.0	33.3	15	31	131,254 (24,511–374,789)
*Staphylococcus aureus,* foodborne	323‡	25.5	29.3	<1	100	241,148 (72,341–529,417)
*Streptococcus* spp. group A, foodborne	15‡	25.5	29.3	<1	100	11,217 (15–77,875)
*Vibrio cholerae,* toxigenic	8§	1.1	33.1	70	100	84 (19–213)
* V. vulnificus*	111§	1.1	1.7	2	47	96 (60–139)
* V. parahaemolyticus*	287§	1.1	142.4	10	86	34,664 (18,260–58,027)
*Vibrio* spp., other	220§	1.1	142.7	11	57	17,564 (10,848–26,475)
* Yersinia enterocolitica*	950¶	1.0	122.8	7	90	97,656 (30,388–172,734)
Subtotal						3,645,773 (2,321,468–5,581,290)
Parasites						
*Cryptosporidium* spp.	7,594¶	1.0	98.6	9	8	57,616 (12,060–166,771)
* Cyclospora cayetanensis*	239¶	1.0	83.1	42	99	11,407 (137–37,673)
* Giardia intestinalis*	20,305§	1.3	46.3	8	7	76,840 (51,148–109,739)
* Toxoplasma gondii*		1.0	0.0	<1	50	86,686 (64,861–111,912)
*Trichinella* spp.	13§	1.3	9.8	4	100	156 (42–341)
Subtotal						232,705 (161,923–369,893)
Viruses						
Astrovirus	NA	NA	NA	0	<1	15,433 (5,569–26,643)
Hepatitis A virus	3,576§	1.1	9.1	41	7	1,566 (702–3,024)
Norovirus	NA	NA	NA	<1	26	5,461,731 (3,227,078–8,309,480)
Rotavirus	NA	NA	NA	0	<1	15,433 (5,569–26,643)
Sapovirus	NA	NA	NA	0	<1	15,433 (5,569–26,643)
Subtotal						5,509,597 (3,273,623–8,355,568)
Total						9,388,075 (6,641,440–12,745,709)

*All estimates based on US population in 2006. Modal or mean value shown unless otherwise stated; see Technical Appendix 3 for the parameters of these distributions. STEC, Shiga toxin–producing *Escherichia coli*; ETEC, enterotoxigenic *E. coli*; NA, not applicable.  †Percentage foodborne among domestically acquired illnesses. ‡Passive surveillance data on outbreak-associated illnesses from the Foodborne Disease Outbreak Surveillance System. Estimates based on the number of foodborne illnesses ascertained in surveillance and therefore assumed to reflect only foodborne transmission. §Passive surveillance data from Cholera and Other *Vibrio* Illness Surveillance or the National Notifiable Disease Surveillance System. ¶Active surveillance data from Foodborne Diseases Active Surveillance Network, adjusted for geographic coverage; data from the National Tuberculosis Surveillance System for *M. bovis*.

### Hospitalizations

We estimate that these 31 pathogens caused 228,744 (90% CrI 188,326–275,601) hospitalizations annually, of which 55,961 (90% CrI 39,534–75,741) were caused by contaminated food eaten in the United States (Table 3). Of these, 64% were caused by bacteria, 27% by viruses, and 9% by parasites. The leading causes of hospitalization were nontyphoidal *Salmonella* spp. (35%), norovirus (26%), *Campylobacter* spp. (15%), and *T. gondii* (8%).

**Table 3:** Estimated annual number of domestically acquired foodborne hospitalizations and deaths caused by 31 pathogens, United States*

Pathogen	Hospitalization rate, %†	Hospitalizations, mean (90% credible interval)	Death rate, %†	Deaths, mean (90% credible interval)
Bacteria				
*Bacillus cereus,* foodborne‡	0.4	20 (0–85)	0	0
*Brucella* spp.	55.0	55 (33–84)	0.9	1 (0–2)
*Campylobacter* spp.	17.1	8,463 (4,300–15,227)	0.1	76 (0–332)
*Clostridium botulinum,* foodborne‡	82.6	42 (19–77)	17.3	9 (0–51)
*Clostridium perfringens,* foodborne‡	0.6	438 (44–2,008)	<0.1	26 (0–163)
STEC O157	46.2	2,138 (549–4,614)	0.5	20 (0–113)
STEC non-O157	12.8	271 (0–971)	0.3	0 (0–0)§
ETEC, foodborne	0.8	12 (0–53)	0	0
Diarrheagenic *E. coli* other than STEC and ETEC	0.8	8 (0–36)	0	0
* Listeria monocytogenes*	94.0	1,455 (521–3,018)	15.9	255 (0–733)
* Mycobacterium bovis*	55.0	31 (21–42)	4.7	3 (2–3)
*Salmonella* spp., nontyphoidal	27.2	19,336 (8,545–37,490)	0.5	378 (0–1,011)
*S. enterica* serotype Typhi	75.7	197 (0–583)	0	0
*Shigella* spp.	20.2	1,456 (287–3,695)	0.1	10 (0–67)
*Staphylococcus aureus*, foodborne‡	6.4	1,064 (173–2,997)	<0.1	6 (0–48)
*Streptococcus* spp. group A, foodborne‡	0.2	1 (0–6)	0	0
*Vibrio cholerae,* toxigenic	43.1	2 (0–5)	0	0
* V. vulnificus*	91.3	93 (53–145)	34.8	36 (19–57)
* V. parahaemolyticus*	22.5	100 (50–169)	0.9	4 (0–17)
*Vibrio* spp., other	37.1	83 (51–124)	3.7	8 (3–19)
* Yersinia enterocolitica*	34.4	533 (0–1,173)	2.0	29 (0–173)
Subtotal		35,796 (21,519–53,414)		861 (260–1,761)
Parasites				
*Cryptosporidium* spp.	25.0	210 (58–518)	0.3	4 (0–19)
* Cyclospora cayetanensis*	6.5	11 (0–109)	0.0	0
* Giardia intestinalis*	8.8	225 (141–325)	0.1	2 (1–3)
* To xoplasma gondii*	2.6	4,428 (2,634–6,674)	0.2	327 (200–482)
*Trichinella* spp.	24.3	6 (0–17)	0.2	0 (0–0)
Subtotal		4,881 (3,060–7,146)		333 (205–488)
Viruses				
Astrovirus	0.4	87 (32–147)	<0.1	0
Hepatitis A virus	31.5	99 (42–193)	2.4	7 (3–15)
Norovirus	0.03	14,663 (8,097–23,323)	<0.1	149 (84–237)
Rotavirus	1.7	348 (128–586)	<0.1	0
Sapovirus	0.4	87 (32–147)	<0.1	0
Subtotal		15,284 (8,719–23,962)		157 (91–245)
Total		55,961 (39,534–75,741)		1,351 (712–2,268)

*All estimates based on US population in 2006. STEC, Shiga toxin–producing *Escherichia coli*; ETEC, enterotoxigenic *E. coli*.  †Unadjusted hospitalization and death rates are presented here. These rates were doubled to adjust for underdiagnosis before being applied to the number of laboratory-confirmed cases to estimate the total number of hospitalizations and deaths. The hospitalization and death rates for astrovirus, norovirus, rotavirus, and sapovirus presented here are the percentage of total estimated illness and were not subject to further adjustment. ‡Estimates based on the number of foodborne illnesses ascertained in surveillance, therefore assumed to reflect only foodborne transmission. §We report median values instead of means for the distributions of deaths caused by STEC non-O157 because of extremely skewed data.

### Deaths

We estimate that these 31 pathogens caused 2,612 deaths (90% CrI 1,723–3,819), of which 1,351 (90% CrI 712–2,268) were caused by contaminated food eaten in the United States (Table 3). Of these, 64% were caused by bacteria, 25% by parasites, and 12% by viruses. The leading causes of death were nontyphoidal *Salmonella* spp. (28%), *T. gondii* (24%), *L. monocytogenes* (19%), and norovirus (11%).

## Discussion

We estimate that foods consumed in the United States that were contaminated with 31 known agents of foodborne disease caused 9.4 million illnesses, 55,961 hospitalizations, and 1,351 deaths each year. Norovirus caused the most illnesses; nontyphoidal *Salmonella* spp., norovirus, *Campylobacter* spp.*,* and *T. gondii* caused the most hospitalizations; and nontyphoidal *Salmonella* spp., *T.* *gondii*, *L. monocytogenes*, and norovirus caused the most deaths. Scarce data precluded estimates for other known infectious and noninfectious agents, such as chemicals. Foodborne diseases are also caused by agents not yet recognized as being transmitted in food and by unknown agents (*22*). The numbers of illnesses caused by these unspecified agents are estimated elsewhere (*4*).

Studies estimating the overall number of foodborne illnesses have been conducted in England and Wales and in Australia (*23**,**24*). Similar to our findings, in Australia norovirus was the leading cause of foodborne illness, accounting for 30% of illnesses caused by known pathogens. In England and Wales, norovirus accounted for only 8% of known foodborne illnesses; however, stool sample reexamination using molecular techniques documented higher rates (*18*). Nontyphoidal *Salmonella* spp. and *Campylobacter* spp. were leading causes of foodborne illnesses in all 3 countries (England and Wales, Australia, and the United States), although nontyphoidal *Salmonella* spp. accounted for a greater proportion of illness in the United States. Recent serologic data from Europe suggest that *Salmonella* spp. infections are more common than estimated by our methods; however, many infections may be asymptomatic (*25*). Our estimates did not capture mild illnesses associated with some pathogens. For example, mild cases of botulism are often recognized as part of outbreaks, but affected persons seldom seek medical care and are not captured by surveillance except during outbreaks (*26**,**27*). Likewise, *L. monocytogenes* is rarely diagnosed as the cause of gastroenteritis and fever, partly because this organism is not detected by routine stool culture (*28*). Early spontaneous abortion or miscarriage associated with listeriosis may also be underdiagnosed.

Accurately estimating hospitalizations and deaths caused by foodborne pathogens is particularly challenging. National data on outpatient visits resulting in hospitalization, hospital discharges, and death certificates probably substantially underestimate pathogen-specific cases because for pathogen-specific diagnoses to be recorded, health care providers must order the appropriate diagnostic tests and coding must be accurate. Particularly in vulnerable populations, dehydration or electrolyte imbalance from a gastrointestinal illness may exacerbate a chronic illness, resulting in hospitalization or death well after resolution of the gastrointestinal illness; thus, the gastrointestinal illness may not be coded as a contributing factor. Moreover, if a pathogen is not detected, infections may be coded as noninfectious illnesses (*29*). For norovirus, we estimated the number of hospitalizations and deaths by applying the estimated proportion of acute gastroenteritis illnesses caused by norovirus to overall estimates of hospitalizations and deaths from acute gastroenteritis; this choice is supported by studies of hospitalizations for norovirus (*30**,**31*). For most other pathogens, we used data from surveillance to estimate pathogen-specific hospitalizations and deaths and doubled the numbers to adjust for underdiagnosis. More precise information about the degree of undercounting of hospitalizations and deaths for each pathogen would improve these estimates.

Our methods and data differed from those used for the 1999 estimates (*3*). Our estimate of medical care seeking among persons with a diarrheal illness, derived from the 3 most recent FoodNet Population Surveys conducted during 2000–2007, was higher than that estimated from the 1996–1997 FoodNet Population Survey used for the 1999 estimates (35% and 18% among persons reporting bloody and nonbloody diarrhea, respectively, compared with 15% and 12% in the earlier [1999] study) (*8*). These data resulted in lower underdiagnosis multipliers, which contributed to lower estimates of number of illnesses. The biggest change from the earlier estimate was the estimated number of norovirus illnesses, which decreased for 2 reasons. First, the number of acute gastrointestinal illnesses estimated from the FoodNet Population Survey and used in the current study was lower than the estimated number of acute gastrointestinal illnesses used in the 1999 assessment. The earlier study used data from 1996–1997; the sample size was one fifth as large as ours and incorporated data from US studies conducted before 1980 (*32**,**33*). Both estimates excluded persons reporting concurrent cough or sore throat, but the proportion of persons reporting these signs and symptoms was higher in the FoodNet Population Surveys we used than that in the older US studies (38% vs. 25%), contributing to a lower estimated prevalence of acute gastroenteritis (0.60 vs. 0.79 episodes/person/year) (*4*,*32**,**33*). Additionally, the current study excluded persons with vomiting who were ill for <1 day or whose illness did not result in restricted daily activities, whereas the earlier study included all vomiting episodes. These factors contributed to the new estimate of acute gastroenteritis being 24% lower than the earlier estimate, more likely the result of increased accuracy than a true decrease in illnesses (*4*). Second, the lower current estimate for norovirus illnesses resulted from a lower proportion of norovirus estimated to be foodborne (decreased from 40% to 26%); this lower proportion is similar to that estimated in recent studies from other countries (*23**,**24*). Because of these reasons and use of other data sources and methods, our estimate cannot be compared with the 1999 estimate for the purpose of assessing trends. FoodNet provides the best data on trends over time (*34*).

Data used in the current study came from a variety of sources and were of variable quality and representativeness. FoodNet sites, from which we used data for 10 pathogens, are not completely representative of the US population, but 1 study indicated that demographic data from FoodNet and from the 2005 US census did not differ much (*6*). For 5 pathogens, only data on foodborne outbreak–related cases were available. No routine surveillance data were available for most viruses, forcing us to use a different modeling approach for viruses than for most other pathogens. Given the large number of norovirus illnesses in these estimates, the paucity of supporting data is a major limitation. Moreover, combining different methods is not optimal because methods themselves may affect the estimates. We chose our modeling approach and used the PERT distribution for many inputs because data were sometimes limited and subjective decisions were required. Other investigators could have chosen other distributions, for good reasons, and arrived at different estimates.

Our assumptions about the proportion of illnesses transmitted by food profoundly affect our estimates, but data on which to base these estimates were often lacking. We used data from surveillance, risk factor studies, and the current literature to estimate the proportion of pathogen-specific illnesses caused by consumption of contaminated food (*35*), but it is not known how representative these data are of total illnesses and whether the foodborne proportion is similar across age groups. For example, the proportion of some illnesses acquired from animals (e.g., STEC O157) may be higher among children than adults (*36*), and the proportions that spread person-to-person (e.g., norovirus) may be higher among institutionalized elderly persons (*37*). Because a higher proportion of cases are reportedly associated with hospitalization or death in these vulnerable groups, we may have overestimated the total contribution of foodborne transmission for these outcomes.

The methods used for this study could be adapted to estimate the proportion of illnesses attributable to other modes of transmission, such as waterborne and direct animal contact. The estimates from this study can be used to help direct policy and interventions; to conduct other analyses (e.g., evaluation of economic cost of these diseases and attribution to various food commodities); and as a platform for developing estimates of effects of disease caused by sequelae of foodborne infections.

## Supplementary Material

Appendix Overview of Methods and Summary of Data Sources.

Appendix Model Structures Used to Make Estimates.

Appendix Estimation and Uncertainty Model Inputs for 31 Major Known Pathogens Transmitted Through Food.

Appendix Data Used to Estimate Passive and Outbreak Surveillance Underreporting Multipliers.

## Figures and Tables

**Figure:**
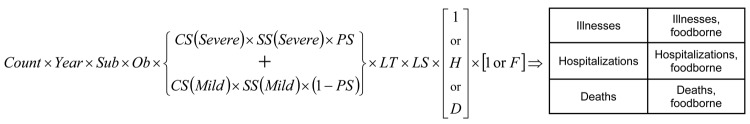
Example schematic diagram of the estimation and uncertainty model used to estimate episodes of illness, hospitalizations, and deaths in the United States. *Count*, data (empirical distribution); *Year,* factor to standardize non-2006 counts to 2006 (constant); *Sub*, expansive factor to scale area surveillance to the entire US population (constant); *Ob,* expansive factor to scale outbreak counts up to outbreak plus sporadic counts (beta distribution); *CS*, expansive factor to scale care seekers to all ill, with severe and mild illness versions (PERT distribution); *SS,* expansive factor to scale submitted samples to all visits, with severe and mild illness versions (PERT distribution); *PS*, estimated proportion of illnesses that are severe (PERT distribution); *LT,* expansive factor to scale tests performed up to samples submitted (PERT distribution); *LS,* expansive factor to scale positive test results up to true positive specimens (PERT distribution); *H,* contractive factor to scale illnesses down to hospitalized illnesses (PERT distribution); *D*, contractive factor to scale illnesses down to deaths (PERT distribution); *F*, contractive factor to scale illnesses down to foodborne illnesses (PERT distribution).

## Appendix

**Table A1:** Data sources used to estimate illnesses, hospitalizations, and deaths caused by 31 pathogens transmitted through food, United States*

Data source	Data	Pathogen(s) or acute gastroenteritis	Geographic coverage	Time frame	Adjustments
COVIS System†	Number of case-patient reports, proportion hospitalized, proportion who died	*Vibrio cholerae,* toxigenic; *V. vulnificus*; *V. parahaemolyticus*; other *Vibrio* spp.	United States	2000–2007	Underreporting; underdiagnosis
FoodNet	Number of laboratory-confirmed illnesses, proportion hospitalized, proportion who died	*Campylobacter* spp.; *Cryptosporidium *spp.; *Cyclospora cayetanensis;* Shiga toxin–producing *Escherichia coli *O157; Shiga toxin-producing *E. coli* non-O157*; Listeria monocytogenes; *non-typhoidal *Salmonella* spp.; *S. enterica* serotype Typhi; *Shigella* spp.; *Yersinia enterocolitica*	FoodNet sites‡	2005–2008	Geographic coverage;§ underdiagnosis
FDOSS	Number of foodborne outbreak-associated illnesses	*Bacillus cereus; Clostridium perfringens; *enterotoxigenic *E. coli; Staphylococcus aureus; Streptococcus *spp.*,* Group A	United States	2000–2007; (*Streptococcus* spp., Group A 1996–2007)¶	Underreporting; underdiagnosis
	Proportion hospitalized and proportion who died in foodborne outbreaks	*Bacillus cereus; C. perfringens;* enterotoxigenic *E. coli; S. aureus; Streptococcus* spp., Group A; *Clostridium botulinum; Trichinella* spp.	United States	2000–2007; (*Streptococcus* spp., Group A 1981–2007)¶	Underdiagnosis
FoodNet Population Survey	Rate of acute gastroenteritis	Average annual rate of acute gastroenteritis was derived by multiplying the average monthly prevalence by 12, where an episode of acute gastroenteritis was defined as diarrhea (>3 loose stools in 24 hours) or vomiting in the past month with both lasting >1 day or resulting in restricted daily activities. Persons with a chronic condition in which diarrhea or vomiting was a major symptom and persons with concurrent symptoms of cough or sore throat were excluded.	FoodNet sites‡	2000–2001, 2002–2003, 2006–2007	Percentage of acute gastroenteritis attributable to norovirus
Multiple-cause-of-death data from the National Vital Statistics System	Death rate	Acute gastroenteritis deaths were identified from the underlying or contributing cause of death classified by ICD-10 diagnostic codes A00.9–A08.5 (infectious gastroenteritis of known cause), A09 (diarrhea and gastroenteritis of presumed infectious origin), or K52.9 (noninfectious gastroenteritis and colitis, unspecified), excluding A04.7 (enterocolitis due to *Clostridium difficile*) and A05.1 (botulism)#	United States	2000–2006	Percentage of acute gastroenteritis deaths attributable to norovirus
NAMCS, NHAMCS	Hospitalization rate	Acute gastroenteritis hospitalizations were identified from patient visits to clinical settings, including physician offices, hospital emergency and outpatient departments with a diagnosis of infectious enteritis [ICD-9-CM diagnostic codes 001–008 (infectious gastroenteritis of known cause), 009 (infectious gastroenteritis), 558.9 (other and unspecified noninfectious gastroenteritis and colitis), or 787.9 (other symptoms involving digestive system: diarrhea), excluding 008.45 (*C, difficile* colitis) and 005.1 (botulism)#]	Nationally representative sample of US clinical settings	2000–2006	Weighted to give national estimates according to NCHS criteria; percentage of acute gastroenteritis hospitalizations (combined with NIS and NHDS) attributable to norovirus
Nationwide Inpatient Sample (NIS)	Hospitalization rate	Acute gastroenteritis hospitalizations were identified from discharges with one of the first three listed diagnoses classified by ICD-9-CM diagnostic codes 001–008 (infectious gastroenteritis of known cause), 009 (infectious gastroenteritis), 558.9 (other and unspecified noninfectious gastroenteritis and colitis), or 787.9 (other symptoms involving digestive system: diarrhea), excluding 008.45 (*C. difficile* colitis) and 005.1 (botulism)#; *Giardia intestinalis* (ICD-9-CM code 007.1); *Toxoplasma gondii* (ICD-9-CM codes 130.0–9)	Sample of discharge records from US hospitals	2000–2006	Weighted to give national estimates according to Healthcare Cost and Utilization Project criteria; Percentage of acute gastroenteritis (combined with NAMCS/NHAMCS and NHDS) hospitalizations attributable to norovirus; underdiagnosis (*G, intestinalis* and *T, gondii* )
	Death rate	*G. intestinalis* (ICD-9-CM code 007.1), *T. gondii* (ICD-9-CM codes 130.0–9)	Sample of discharge records from US hospitals	2000–2006	Underdiagnosis
National Health and Nutrition Examination Survey	Seroprevalence	*T. gondii*	United States	1999–2004	Rate of infection over time and percent symptomatic
NHDS	Hospitalization rate	Acute gastroenteritis hospitalizations were identified from discharges with one of the first three listed diagnoses classified by ICD-9-CM diagnostic codes 001–008 (infectious gastroenteritis of known cause), 009 (infectious gastroenteritis), 558.9 (other and unspecified noninfectious gastroenteritis and colitis), or 787.9 (other symptoms involving digestive system: diarrhea), excluding 008.45 (*C. difficile* colitis) and 005.1 (botulism)#	Nationally representative sample of discharge records from US hospitals	2000–2006	Weighted to give national estimates according to NCHS criteria; percentage of acute gastroenteritis hospitalizations (combined with NAMCS/NHAMCS and NIS) attributable to norovirus
National Notifiable Disease Surveillance System	Number of case-patient reports	*Brucella* spp.; *C. botulinum* (foodborne); *Trichinella* spp*.*; hepatitis A; *G. intestinalis*	United States	2000–2007 (2002–2007 for *G. intestinalis*)**	Underreporting; underdiagnosis
	Hospitalization rate	Hepatitis A	United States	2000–2007	Underdiagnosis
National Tuberculosis Surveillance System	Number of tuberculosis case-patient reports, proportion who died	*Mycobacterium bovis*	United States	2004–2007	Percentage of tuberculosis cases attributable to *M. bovis*; underdiagnosis
US Census	Resident population estimates	Astrovirus, rotavirus, sapovirus	United States	2006	75% of children experience an episode of clinical illness by 5 years of age.

*COVIS, Cholera and Other *Vibrio* Illness Surveillance; FoodNet, Foodborne Diseases Active Surveillance Network; FDOSS, Foodborne Disease Outbreak Surveillance System; ICD-10, International Classification of Diseases, 10th Revision; NAMCS, National Ambulatory Medical Care Survey; NHAMCS, National Hospital Ambulatory Medical Care Survey; ICD-9-CM, International Classification of Diseases, 9th Revision, Clinical Modification; NCHS, National Center for Health Statistics; NIS, Nationwide Inpatient Sample; NHDS, National Hospital Discharge Survey. †Passive surveillance from COVIS was used in preference to active surveillance from FoodNet for *Vibrio* spp. because most illnesses are reported by Gulf Coast States (Florida, Alabama, Louisiana, Texas) that are not included in the FoodNet surveillance area. ‡Beginning in 2000, there were 10 FoodNet sites. In 2008, the population of these sites was 45 million persons, 15% of the US population. §Incidence of laboratory-confirmed illnesses in FoodNet for 2005–2008 was applied to 2006 US Census population estimates. ¶Data from FDOSS on *Streptococcus* spp., group A, were included for 1996–2007 for illnesses and for 1981–2007 for hospitalizations and deaths because of a paucity of data (Technical Appendix 1, Technical Appendix 3). #Codes for other and unspecified noninfectious gastroenteritis and colitis were included because infectious illnesses of unknown etiology are sometimes coded as noninfectious. ***G. intestinalis* became nationally notifiable in 2002.
