# Supplementary material for: Foodborne Illness Acquired in the United States—Major Pathogens
Source: Emerg Infect Dis. 2011 Jan;17(1):7–15. doi: 10.3201/eid1701.P11101 (PMC3375761; doi:10.3201/eid1701.P11101)
Supplement: Appendix — Overview of Methods and Summary of Data Sources. [file 09-1101p1-Techapp1.pdf]

Article DOI: 10.3201/eid1701.P11101

Foodborne Illness Acquired in the United States—Major Pathogens

Technical Appendix 1

Overview of Methods and Summary of Data Sources

This appendix provides a summary of data sources and methods used to estimate the annual number of illnesses, the annual number of hospitalizations, the annual number of deaths, the proportion travel-related, and the proportion foodborne for 31 major known pathogens transmitted through food

This appendix includes only modal values.
Technical Appendix 2 (www.cdc.gov/EID/content/17/1/7-Techapp2.pdf) contains a full description of the uncertainty model parameters.

**Pathogen: Astrovirus**	
**Estimate**	**Data source(s)**
**Number of illnesses**	There is evidence suggesting high seroprevalences of astroviruses in young children (1); therefore, we assumed that 75% of children experience an episode of clinical illness due to astrovirus by 5 years of age similar to other childhood gastroenteritis viruses such as rotavirus (2). The person-time at risk for 2006 was estimated as the 0-4 year population (20,417,636) divided by 5 and rounded (4,084,000) (3).
**Number of hospitalizations**	Assumed to equal 25% of number of hospitalizations for rotavirus based on published studies (4).
**Number of deaths**	Assumed to be very low: <10 deaths per year.
**Proportion travel-related**	Assumed to be 100% domestically acquired.
**Proportion foodborne**	Very low (<1%) based on published review (5).
**Comments**	Significant illness assumed to occur only among children <5 years of age. Very few foodborne outbreaks reported (CDC, unpublished data).

**Pathogen:** ***Bacillus cereus***	
**Estimate**	**Data source(s) and method**
**Number of illnesses**	Annual number of *Bacillus cereus* outbreak-associated illnesses reported to CDC's Foodborne Disease Outbreak Surveillance System (2000–2007) (6), adjusted for underreporting due to surveillance type (see outbreak surveillance underreporting multiplier described in online Technical Appendixes 2 and 4 (www.cdc.gov/EID/content/17/1/zzz-Techapp4.pdf) and under-diagnosis resulting from the following surveillance steps: medical care seeking, specimen submission, laboratory testing, and test sensitivity (see online Technical Appendix 2).
**Number of hospitalizations**	Proportion hospitalized in *Bacillus cereus* outbreaks reported to the Foodborne Disease Outbreak Surveillance System (2000–2007) applied to the estimated number of reported illnesses (after adjusting for underreporting) and doubled to adjust for under-diagnosis.
**Number of deaths**	Proportion who died in *Bacillus cereus* outbreaks reported to the Foodborne Disease Outbreak Surveillance System (2000–2007) applied to the estimated number of reported illnesses (after adjusting for underreporting) and doubled to adjust for under-diagnosis.
**Proportion travel-related**	Because of the rapid onset and short duration of *Bacillus cereus* illnesses, we assumed that almost all *Bacillus cereus* illnesses occurring in the United States were domestically acquired.
**Proportion foodborne**	Our estimate of the number of illnesses was based on outbreak-associated *Bacillus cereus* illnesses reported to CDC through the Foodborne Disease Outbreak Surveillance System. Because all these outbreaks were foodborne, our estimate of the number of illnesses was based solely on foodborne outbreak-associated illnesses. Therefore, 100% of the estimated number of illnesses was considered foodborne.

**Pathogen: *Brucella* spp.**	
**Estimate**	**Data source(s)**
**Number of illnesses**	Annual number of brucellosis illnesses reported to CDC's National Notifiable Disease Surveillance System (NNDSS) (2000–2007) (7); adjusted for underreporting due to surveillance type (see passive surveillance underreporting multiplier described in online Technical Appendixes 2 and 4) and under-diagnosis resulting from the following surveillance steps: medical care seeking, specimen submission, laboratory testing, and test sensitivity (see online Technical Appendix 2).
**Number of hospitalizations**	Proportion (55%) hospitalized in *Brucella* spp. outbreaks reported to the CDC (CDC, unpublished data) applied to the estimated number of reported illnesses (after adjusting for underreporting) and doubled to adjust for under-diagnosis.
**Number of deaths**	Death rate among persons with brucellosis was 0.9% in studies in California and Texas (8, 9). This proportion was applied to the estimated number of reported illnesses (after adjusting for underreporting) and doubled to adjust for under-diagnosis.
**Proportion travel-related**	16% of cases of brucellosis reported to NNDSS (2000-2007) were reported to have acquired their infection outside the United States.
**Proportion foodborne**	We used the estimate of 50% foodborne used by Mead *et al.* (1999) (10). Overall, consumption of milk or cheese products from Mexico was implicated in 45% of cases reported from California from 1973 to 1992 (9). Because the proportion of cases due to foodborne transmission was higher in the latter half of this period, 50% of cases were assumed to be foodborne.
**Comments**	Reports from California and Texas account for most illnesses.

**Pathogen: *Campylobacter* spp.**	
**Estimate**	**Data source(s)**
**Number of illnesses**	Annual incidence of campylobacterosis reported to CDC's Foodborne Diseases Active Surveillance Network (FoodNet) sites (2005-2008) (11); adjusted for geographical coverage (FoodNet is in 10 sites around the United States) and under-diagnosis resulting from the following surveillance steps: medical care seeking, specimen submission, laboratory testing, and test sensitivity (see online Technical Appendix 2).
**Number of hospitalizations**	Proportion of FoodNet cases of *Campylobacter* spp. infection hospitalized (2005–2008) applied the estimated number of reported cases (after adjusting for geographical coverage) and doubled to adjust for under-diagnosis.
**Number of deaths**	Proportion of FoodNet cases of *Campylobacter* spp. infection who died (2005–2008) applied the estimated number of reported cases (after adjusting for geographical coverage) and doubled to adjust for under-diagnosis.
**Proportion travel-related**	20% based on surveillance data from FoodNet (2005–2008). Cases of *Campylobacter* spp. infection in FoodNet were queried about international travel in the seven days before illness began. Estimates were based on cases with known travel history.
**Proportion foodborne**	80% based on a FoodNet case-control study of sporadic *Campylobacter* illnesses (12). We assumed a total population attributable fraction of 100% and subtracted from this the non-foodborne population attributable fractions from the case-control study (non-foodborne risk factors included: Had contact with animal stool [6%]; Had pet puppy [5%]; Had contact with farm animals [6%]; Drank untreated water from a lake, river, or stream [3%]). The remaining fraction (80%) was assumed to be due to contaminated food.

**Pathogen:** ***Clostridium botulinum***	
**Estimate**	**Data source(s)**
**Number of illnesses**	Annual number of foodborne botulism illnesses reported to CDC's National Notifiable Disease Surveillance System (NNDSS) (2000–2007) (7); adjusted for underreporting due to surveillance type (see passive surveillance underreporting multiplier described in online Technical Appendixes 2 and 4) and under-diagnosis resulting from the following surveillance steps: medical care seeking, specimen submission, laboratory testing, and test sensitivity (see online Technical Appendix 2).
**Number of hospitalizations**	Proportion hospitalized in foodborne botulism outbreaks reported to the Foodborne Disease Outbreak Surveillance System (2000–2007) (6) applied to the estimated number of reported illnesses (after adjusting for underreporting) and doubled to adjust for under-diagnosis.
**Number of deaths**	Proportion who died in foodborne botulism outbreaks reported to the Foodborne Disease Outbreak Surveillance System (2000–2007) applied to the estimated number of reported illnesses (after adjusting for underreporting) and doubled to adjust for under-diagnosis.
**Proportion travel-related**	Almost all cases reported to CDC's botulism surveillance were domestically acquired.
**Proportion foodborne**	Estimates based on the number of illnesses reported as foodborne botulism (as opposed to wound botulism or infant botulism); therefore, assumed to be 100% foodborne.
**Comments**	Almost all cases of foodborne botulism reported to CDC are in persons hospitalized for life-threatening manifestations. Mild cases of botulism are often recognized as part of outbreaks (13, 14), but these persons seldom seek medical care and so are not likely to be captured in routine surveillance.

**Pathogen:** ***Clostridium perfringens***	
**Estimate**	**Data source(s)**
**Number of illnesses**	Annual number of *Clostridium perfringens* outbreak-associated illnesses reported to CDC's Foodborne Disease Outbreak Surveillance System (2000–2007) (6); adjusted for underreporting due to surveillance type (see outbreak surveillance underreporting multiplier described in online Technical Appendixes 2 and 4) and under-diagnosis resulting from the following surveillance steps: medical care seeking, specimen submission, laboratory testing, and test sensitivity (see online Technical Appendix 2).
**Number of hospitalizations**	Proportion hospitalized in *Clostridium perfringens* outbreaks reported to the Foodborne Disease Outbreak Surveillance System (2000–2007) applied to the estimated number of reported illnesses (after adjusting for underreporting) and doubled to adjust for under-diagnosis.
**Number of deaths**	Proportion who died in *Clostridium perfringens* outbreaks reported to the Foodborne Disease Outbreak Surveillance System (2000–2007) applied to the estimated number of reported illnesses (after adjusting for underreporting) and doubled to adjust for under-diagnosis.
**Proportion travel-related**	Because of the rapid onset and short duration of *Clostridium perfringens* illnesses, we assumed that 100% *Clostridium perfringens* illnesses occurring in the United States were domestically acquired.
**Proportion foodborne**	Our estimate of the number of illnesses was based on outbreak-associated *Clostridium perfringens* illnesses reported to CDC through the Foodborne Disease Outbreak Surveillance System. Because all these outbreaks were foodborne, our estimate of the number of illnesses was based solely on foodborne outbreak-associated illnesses. Therefore, 100% of the estimated number of illnesses was considered foodborne.

**Pathogen: *Cryptosporidium* spp.**	
**Estimate**	**Data source(s)**
**Number of illnesses**	Annual incidence of cryptosporidiosis reported to CDC's Foodborne Diseases Active Surveillance Network (FoodNet) sites (2005-2008) (11); adjusted for geographical coverage (FoodNet is in 10 sites around the United States) and under-diagnosis resulting from the following surveillance steps: medical care seeking, specimen submission, laboratory testing, and test sensitivity (see online Technical Appendix 2).
**Number of hospitalizations**	Proportion of FoodNet cases of *Cryptosporidium* spp. infection hospitalized (2005–2008) applied the estimated number of reported cases (after adjusting for geographical coverage) and doubled to adjust for under-diagnosis.
**Number of deaths**	Proportion of FoodNet cases of *Cryptosporidium* spp. infection who died (2005–2008) applied the estimated number of reported cases (after adjusting for geographical coverage) and doubled to adjust for under-diagnosis.
**Proportion travel-related**	9% based on surveillance data from FoodNet (2005–2008). Cases of *Cryptosporidium* spp. infection in FoodNet were queried about international travel in the 15 days before illness began. Estimates were based on cases with known travel history.
**Proportion foodborne**	We estimated that 8% of cases were foodborne based on data from a Canadian study (15).

Pathogen: *Cyclospora cayetanensis*	
**Estimate**	**Data source(s)**
**Number of illnesses**	Annual incidence of *Cyclospora cayetanensis* infection reported to CDC's Foodborne Diseases Active Surveillance Network (FoodNet) sites (2005-2008) (11); adjusted for geographical coverage (FoodNet is in 10 sites around the United States) and under-diagnosis resulting from the following surveillance steps: medical care seeking, specimen submission, laboratory testing, and test sensitivity (see online Technical Appendix 2).
**Number of hospitalizations**	Proportion of FoodNet cases of *Cyclospora cayetanensis* infection hospitalized (2005–2008) applied the estimated number of reported cases (after adjusting for geographical coverage) and doubled to adjust for under-diagnosis.
**Number of deaths**	Proportion of FoodNet cases of *Cyclospora cayetanensis* infection who died (2005–2008) applied the estimated number of reported cases (after adjusting for geographical coverage) and doubled to adjust for under-diagnosis.
**Proportion travel-related**	42% based on surveillance data from FoodNet (2005–2008). Cases of *Cyclospora cayetanensis* infection in FoodNet were queried about international travel in the 15 days before illness began. Estimates were based on cases with known travel history.
**Proportion foodborne**	99% based on outbreaks reported to CDC; foodborne outbreaks have been identified during most years since the mid 1990's and have been associated with various types of imported fresh produce (16, 17).
**Comments**	*Cyclospora cayetanensis* infection appears to be most common in tropical and subtropical regions and is not thought to be endemic in the United States (localized, low-level endemicity cannot be excluded). The main identified risk factor for domestic acquisition of infection is consumption of contaminated fresh produce imported from cyclosporiasis-endemic areas. Importation, distribution, and consumption of contaminated produce are not uniform in place or time. The "true" number of affected persons could range from 0 to many thousands from year to year. (In some years, documented cases have exceeded 1,000). FoodNet data/estimates were used for methodologic consistency but should be interpreted with caution, as marked geographic and temporal variability (both "true" and artifactual) confound

	attempts to generalize from particular sites and years.

**Pathogen: *E. coli,* enterotoxigenic (ETEC)**	
**Estimate**	**Data source(s)**
**Number of illnesses**	Annual number of ETEC outbreak-associated illnesses reported to CDC's Foodborne Disease Outbreak Surveillance System (2000–2007) (6); adjusted for underreporting due to surveillance type (see outbreak surveillance underreporting multiplier described in online Technical Appendixes 2 and 4) and under-diagnosis resulting from the following surveillance steps: medical care seeking, specimen submission, laboratory testing, and test sensitivity (see online Technical Appendix 2).
**Number of hospitalizations**	Proportion hospitalized in ETEC outbreaks reported to the Foodborne Disease Outbreak Surveillance System (2000–2007) applied to the estimated number of reported illnesses (after adjusting for underreporting) and doubled to adjust for under-diagnosis.
**Number of deaths**	Proportion who died in ETEC outbreaks reported to the Foodborne Disease Outbreak Surveillance System (2000–2007) applied to the estimated number of reported illnesses (after adjusting for underreporting) and doubled to adjust for under-diagnosis.
**Proportion travel-related**	55% based on surveillance data from Minnesota FoodNet site (Minnesota Department of Health, unpublished data). Estimates were based on cases with a known travel history.
**Proportion foodborne**	Our estimate of the number of illnesses was based on outbreak-associated ETEC illnesses reported to CDC through the Foodborne Disease Outbreak Surveillance System. Because all these outbreaks were foodborne, our estimate of the number of illnesses was based solely on foodborne outbreak-associated illnesses. Therefore, 100% of the estimated number of illnesses was considered foodborne.
**Comments**	Many sporadic cases are associated with travel to other countries where both water and foodborne exposures are likely.

**Pathogen:** ***Escherichia coli*** **O157, Shiga toxin–producing (STEC O157)**	
**Estimate**	**Data source(s)**
**Number of illnesses**	Annual incidence of STEC O157 infections reported to CDC's Foodborne Diseases Active Surveillance Network (FoodNet) sites (2005-2008) (11); adjusted for geographical coverage (FoodNet is in 10 sites around the United States) and under-diagnosis resulting from the following surveillance steps: medical care seeking, specimen submission, laboratory testing, and test sensitivity (see online Technical Appendix 2).
**Number of hospitalizations**	Proportion of FoodNet cases of STEC O157 infection hospitalized (2005–2008) applied the estimated number of reported cases (after adjusting for geographical coverage) and doubled to adjust for under-diagnosis.
**Number of deaths**	Proportion of FoodNet cases of STEC O157 infection who died (2005–2008) applied the number of reported cases (after adjusting for geographical coverage) and doubled to adjust for under-diagnosis.
**Proportion travel-related**	3.5% based on surveillance data from FoodNet (2005–2008). Cases of STEC O157 infection in FoodNet were queried about international travel in the seven days before illness began. Estimates were based on cases with known travel history.
**Proportion foodborne**	68% based on outbreak-associated illnesses from outbreaks reported to CDC from 1982-2002 (18) for which a mode of transmission was known.

Pathogen: *E. coli*, Shiga-toxin-producing (STEC), non-O157	
**Estimate**	**Data source(s)**
**Number of illnesses**	Annual incidence of non-O157 STEC reported to CDC's Foodborne Diseases Active Surveillance Network (FoodNet) (2005-2008) (11); adjusted for geographical coverage (FoodNet is in 10 sites around the United States) and under-diagnosis resulting from the following surveillance steps: medical care seeking, specimen submission, laboratory testing, and test sensitivity (see online Technical Appendix 2). Laboratory testing and test sensitivity multipliers based on evidence that non-O157 STEC is at least as common as STEC O157 (19-21).
**Number of hospitalizations**	Proportion of FoodNet cases of non-O157 STEC infection hospitalized (2005–2008) applied the estimated number of reported cases (after adjusting for geographical coverage) and doubled to adjust for under-diagnosis.
**Number of deaths**	Proportion of FoodNet cases of non-O157 STEC infection who died (2005–2008) applied the estimated number of reported cases (after adjusting for geographical coverage) and doubled to adjust for under-diagnosis.
**Proportion travel-related**	18% based on surveillance data from FoodNet (2005–2008). Cases of non-O157 STEC infection in FoodNet were queried about international travel in the seven days before illness began. Estimates were based on cases with known travel history.
**Proportion foodborne**	82% based outbreak-associated illnesses from outbreaks reported to CDC from 1990-2008 (22).
**Comments**	There is good evidence that, when appropriate laboratory methods are employed, non-O157 STEC infections are as common as STEC O157 infections (19-21). Non-O157 STEC infections are, however, less likely to cause bloody diarrhea (21).

**Pathogen: *E. coli,* diarrheagenic other than STEC and ETEC**	
**Estimate**	**Data source(s)**
**Number of illnesses**	Assumed to be as common as ETEC
**Number of hospitalizations**	Assumed to be as common as ETEC
**Number of deaths**	Assumed to be as common as ETEC
**Proportion travel-related**	Assumed to be almost 100% domestically acquired.
**Proportion foodborne**	Very little data available; a few foodborne outbreaks have been reported. Assumed to be 30% foodborne (10).
**Comments**	Includes enteropathogenic *E. coli*, enteroaggregative *E. coli*, enteroinvasive *E. coli*, and other poorly defined pathogenic groups. Little data are available on the incidence of these infections in the United States; however, some studies suggest that these pathogens are under-recognized (23, 24).

**Pathogen:** ***Giardia intestinalis***	
**Estimate**	**Data source(s)**
**Number of illnesses**	Annual number of cases of *Giardia intestinalis* infection reported to CDC's National Notifiable Disease Surveillance System (NNDSS) (2002–2007) (7); adjusted for underreporting due to surveillance type (see passive surveillance underreporting multiplier described in online Technical Appendixes 2 and 4) and under-diagnosis resulting from the following surveillance steps: medical care seeking, specimen submission, laboratory testing, and test sensitivity (see online Technical Appendix 2).
**Number of hospitalizations**	Estimated based on national estimates of hospital discharge from the Healthcare Cost and Utilization Project (HCUP) Nationwide Inpatient Sample (NIS) (2002-2006) (25) using ICD-9-CM code 007.1 (Giardiasis) and doubled to adjust for under-diagnosis.
**Number of deaths**	Estimated based on national estimates of inpatient deaths from NIS (2002-2006) using ICD-9-CM code 007.1 (Giardiasis) and doubled to adjust for under-diagnosis.
**Proportion travel-related**	8% based on a published study (26).
**Proportion foodborne**	7% based on outbreaks reported to CDC (CDC, unpublished data).
**Comments**	*Giardia intestinalis* only became nationally notifiable in 2002.

Pathogen: Hepatitis A	
**Estimate**	**Data source(s)**
**Number of illnesses**	Annual number of cases of hepatitis A infection reported to CDC's National Notifiable Disease Surveillance System (NNDSS) (2000–2007) (7, 27) with an adjustment for trend (recent years were weighted more heavily) (see online Technical Appendix 3, www.cdc.gov/EID/content/17/1/zzz-Techapp2.pdf); adjusted for underreporting due to surveillance type (see passive surveillance underreporting multiplier described in online Technical Appendixes 2 and 4) and under-diagnosis resulting from the following surveillance steps: medical care seeking, specimen submission, laboratory testing, and test sensitivity (see online Technical Appendix 2).
**Number of hospitalizations**	Proportion of hepatitis A cases reported to NNDSS (2001–2007) who were hospitalized applied to the estimated number of reported illnesses (after adjusting for underreporting) and doubled to adjust for under-diagnosis. Data from 2001 were used because hospitalizations were more carefully evaluated since 2001.
**Number of deaths**	Number of deaths estimated using data from the National Center for Health Statistics (NCHS) multiple cause-of-death data from the National Vital Statistics System (28) and doubled to adjust for under-diagnosis.
**Proportion travel-related**	41% based on enhanced surveillance in 6 US states (2005-2007) (29).
**Proportion foodborne**	6% based on exposure data from NNDSS (2000-2007).

**Pathogen:** ***Listeria monocytogenes***	
**Estimate**	**Data source(s)**
**Number of illnesses**	Annual incidence of listeriosis reported to CDC's Foodborne Diseases Active Surveillance Network (FoodNet) sites (2005-2008) (11); adjusted for geographical coverage (FoodNet is in 10 sites around the Unites States) and under-diagnosis resulting from the following surveillance steps: medical care seeking, specimen submission, laboratory testing, and test sensitivity (see online Technical Appendix 2). All invasive cases of listeriosis reported to FoodNet were included (including cases of congenital and acquired listeriosis). A case of invasive listeriosis was defined as isolation of *Listeria monocytogenes* from a normally sterile site (e.g., blood, cerebrospinal fluid, or amniotic □uid) or from the placenta or products of conception. Mother-infant pairs were counted separately.
**Number of hospitalizations**	Proportion of FoodNet cases of *Listeria monocytogenes* infection hospitalized (2005–2008) applied the estimated number of reported cases (after adjusting for geographical coverage) and doubled to adjust for under-diagnosis.
**Number of deaths**	Proportion of FoodNet cases of *Listeria monocytogenes* infection who died (2005–2008) applied the estimated number of reported cases (after adjusting for geographical coverage) and doubled to adjust for under-diagnosis.
**Proportion travel-related**	3% based on surveillance data from FoodNet (2005–2008). Cases of listeriosis in FoodNet were queried about international travel in the 30 days before illness began. Estimates were based on cases with known travel history.
**Proportion foodborne**	Almost 100%. Microbiologic and epidemiologic data demonstrate that food is the source of infection almost all cases (30-34).
**Comments**	*Listeria monocytogenes* can cause febrile gastroenteritis (35), but these illnesses are rarely diagnosed, at least partly because clinical laboratories do not routinely test stool specimens for *Listeria*. Listeriosis can result in spontaneous abortion or miscarriage; these infections may also be under-represented here.

**Pathogen:** ***Mycobacterium bovis***	
**Estimate**	**Data source(s)**
**Number of illnesses**	Annual number of cases of tuberculosis reported to CDC's National Tuberculosis Surveillance System (NTSS) (2004–2007) (36); adjusted for the proportion of tuberculosis attributable to *Mycobacterium bovis* (37) and under-diagnosis resulting from the following surveillance steps: medical care seeking, specimen submission, laboratory testing, and test sensitivity (see online Technical Appendix 2).
**Number of hospitalizations**	Limited data available on *Mycobacterium bovis.* Proportion hospitalized assumed to be 55% based on a study of hospitalizations among persons with tuberculosis (38).
**Number of deaths**	Limited data available on *Mycobacterium bovis*. Assumed to be equal the proportion of tuberculosis cases reported to NTSS (2004–2007) who died.
**Proportion travel-related**	Limited data available on *Mycobacterium bovis*. The majority of persons who have tuberculosis come from countries where the infection is prevalent in cattle and where they presumably acquired infection (39). 70% of cases assumed to be travel-related.
**Proportion foodborne**	Most cases (95%) assumed to be foodborne (39). Historically, *Mycobacterium bovis* disease in humans was associated with consumption of unpasteurized milk from infected cattle. Successful *Mycobacterium bovis* eradication programs have nearly eradicated the disease in industrialized countries. In the United States, the disease is almost exclusively confined to Mexican-born adults and US-born Hispanic children. *Mycobacterium bovis* is still found in Mexican dairy herds and one study reported that consumption of Mexican dairy products, especially cheese and cream, is common among patients with *Mycobacterium bovis* infection (39).
**Comment**	Nationally, 1.4% of TB cases were attributed to *Mycobacterium bovis.* However, in the San Diego, California the incidence of *Mycobacterium bovis* has been noted to be increasing. In a retrospective analysis of TB case surveillance data between 1994 and 2005, the annual proportion of culture-positive TB cases attributed to *Mycobacterium bovis* increased from 5 to 11%. From 2001-2005, *Mycobacterium bovis* accounted for 10% of reported cases (40).

**Pathogen: Norovirus**	
**Estimate**	**Data source(s)**
**Number of illnesses**	• Proportion of acute gastroenteritis due to norovirus (11%) estimated from studies in the Netherlands (41), England and Wales (42, 43), and Australia (44) was applied to the estimated number of acute gastroenteritis illnesses in the United States.<br>• Rate of acute gastroenteritis illnesses in the United States was estimated using combined data from FoodNet Population Surveys in 2000–2001, 2002–2003, and 2006–2007 (CDC, unpublished data). The FoodNet Population Surveys are random-digit-dial telephone surveys of the general FoodNet population conducted over 12-month periods. Information was collected on episodes of gastrointestinal symptoms in the past month. Methods are described in detail elsewhere (45).<br>• Average annual rate of acute gastroenteritis was derived by multiplying the average monthly prevalence by 12, where an episode of acute gastroenteritis was defined as diarrhea (≥3 loose stools in 24 hours) or vomiting in the past month with both lasting >1 day or resulting in restricted daily activities. Persons with a chronic condition in which diarrhea or vomiting was a major symptom and persons with concurrent symptoms of cough or sore throat were excluded. Data were weighted to compensate for unequal probabilities of selection and to reflect the surveillance population by age and sex.<br>• Number of acute gastroenteritis illnesses was estimated by applying the average rate of acute gastroenteritis from the combined surveys (0.6 episodes per person per year) to the 2006 US Census population estimate (299 million persons) (3). The rate from individual surveys was 0.49 (2000-2001), 0.54 (2002-2003), and 0.73 episodes per person per year (2006-2007).
**Number of hospitalizations**	• Proportion of acute gastroenteritis due to norovirus (11%) estimated from studies in the Netherlands (41), England and Wales (42, 43), and Australia (44) was applied to the estimated number of acute gastroenteritis hospitalizations in the United States. The decision to apply this proportion to hospitalizations was supported by published studies (46, 47).<br>• The hospitalization rate for acute gastroenteritis was estimated using 2000-2006 data from three sources:

	1) CDC's National Center for Health Statistics (NCHS) National Hospital Discharge System (NHDS) (48, 49)
**Estimate**	**Data source(s)**
	2) Healthcare Cost and Utilization Project (HCUP) Nationwide Inpatient Sample (NIS) (25); and<br>3) Combined data from CDC's NCHS National Ambulatory and National Hospital Ambulatory Medical Care Surveys (NAMCS/NHAMCS) (50)<br>• The International Classification of Diseases, Ninth Revision, Clinical Modification (ICD-9-CM) codes were used to extract hospital discharges from NHDS and NIS where acute gastroenteritis was listed as one of the first three diagnoses. Acute gastroenteritis was defined as ICD-9-CM diagnostic codes 001–008 (infectious gastroenteritis of known cause); 009 (infectious gastroenteritis); 558.9 (other and unspecified noninfectious gastroenteritis and colitis); or 787.9 (other symptoms involving digestive system: diarrhea); excluding 008.45 (*Clostridium difficile* colitis) and 005.1 (botulism). Hospital discharge records were selected on the basis of the first three listed diagnoses as a compromise between limiting the analysis to hospitalizations where acute gastroenteritis was listed as the primary cause and including all hospitalizations in which it was listed. This approach has been taken in other studies (51, 52). National estimates from NHDS and NIS were obtained for each year (2000-2006) by weighting the sample data according to the NCHS and HCUP criteria.<br>• To estimate acute gastroenteritis hospitalizations from NAMCS/NHAMCS from 2000-2006, we combined data across the two surveys and extracted patient visits to clinical settings, including physician offices, hospital emergency and outpatient departments with a diagnosis of acute gastroenteritis resulting in hospitalization. Acute gastroenteritis was defined using the same ICD-9-CM codes as described above for NHDS and NIS. Hospitalization rates for each year were obtained by weighting the sample data according to the NCHS criteria.<br>• We estimated the number of hospitalizations by applying the mean (166 per 100,000) of these 21 annual hospitalization rates to the 2006 US Census population estimate. The mean rate from 2000-2006 NHDS data was 203 per 100,000 persons; the mean rate from 2000-2006 NIS data was 187 per 100,000, the rate from combined NAMCS/NHAMCS data was 109 per 100,000 persons.

Pathogen: Norovirus (continued)	
**Estimate**	**Data source(s)**
**Number of deaths**	• Proportion of acute gastroenteritis due to norovirus (11%) estimated from studies in the Netherlands (41), England and Wales (42, 43), and Australia (44) was applied to an estimate of the number of acute gastroenteritis deaths in the United States.<br>• The death rate for acute gastroenteritis was estimated using multiple cause-of-death data from the National Vital Statistics System (2000–2006) (28, 53) where acute gastroenteritis was listed as the underlying or a contributing cause. Acute gastroenteritis was defined as ICD-10 diagnostic codes A00.9–A08.5 (infectious gastroenteritis of known cause); A09 (diarrhea and gastroenteritis of presumed infectious origin); and K52.9 (noninfectious gastroenteritis and colitis, unspecified)]; excluding A04.7 (enterocolitis due to *Clostridium difficile*) and A05.1 (botulism).<br>• We estimated the number of acute gastroenteritis deaths by applying the mean death rate from 2000-2006 (1.5 per 100,000 population) to the 2006 US Census population estimate.
**Proportion travel-related**	• Important cause of traveler's diarrhea, but this proportion is estimated to be small (<1% of illnesses) given the large number of domestically acquired illnesses and the short incubation period.
**Proportion foodborne**	• Based on 179 norovirus outbreaks examined by CDC from 2000-2005. Of 13,944 persons ill, 3,628 (26%) were in foodborne outbreaks (CDC, unpublished data).

**Pathogen: Rotavirus**	
**Estimate**	**Data source(s)**
**Number of illnesses**	We assumed that 75% of children experience an episode of clinical illness due to rotavirus by 5 years of age based on a published study (2). The person-time at risk for 2006 was estimated as the 0-4 year population (20,417,636) divided by 5 and rounded (4,084,000) (3).
**Number of hospitalizations**	Based on published studies (2).
**Number of deaths**	Very low: 20 to 40 deaths per year (2).
**Proportion travel-related**	Assumed to be almost 100% domestically acquired since international travel-associated illness among young children is likely small compared to the domestic burden.
**Proportion foodborne**	Assumed to be very low (<1% of illnesses) based on the number of foodborne outbreaks reported to CDC (6)

**Pathogen: *Salmonella enterica,* non-typhoidal serotypes**	
**Estimate**	**Data source(s)**
**Number of illnesses**	Annual incidence of salmonellosis reported to CDC's Foodborne Diseases Active Surveillance Network (FoodNet) sites (2005-2008) (11); adjusted for geographical coverage (FoodNet is in 10 sites around the United States) and under-diagnosis resulting from the following surveillance steps: medical care seeking, specimen submission, laboratory testing, and test sensitivity (see online Technical Appendix 2). In all analyses in this paper, serotype Paratyphi is grouped with non-typhoidal S*almonella*.
**Number of hospitalizations**	Proportion of FoodNet cases of non-typhoidal *Salmonella* infection hospitalized (2005–2008) applied the estimated number of reported cases (after adjusting for geographical coverage) and doubled to adjust for under-diagnosis.
**Number of deaths**	Proportion of FoodNet cases of non-typhoidal *Salmonella* infection who died (2005–2008) applied the estimated number of reported cases (after adjusting for geographical coverage) and doubled to adjust for under-diagnosis.
**Proportion travel-related**	11% based on surveillance data from FoodNet (2005–2008). Cases of non-typhoidal *Salmonella* infection in FoodNet were queried about international travel in the seven days before illness began. Estimates were based on cases with known travel history.
**Proportion foodborne**	94% based on FoodNet case-control study of sporadic illness (54) and outbreaks reported to CDC from 1996-2006 (CDC, unpublished data). In the FoodNet study, 6% of cases of non-typhoidal *Salmonella* infections were attributed to reptile exposure; questions were asked about other animals and water, but no illnesses were attributed to these exposures. Adding all of the outbreak-associated illnesses reported to CDC from 1996-2006, 96% were associated with foodborne transmission, 3% with animal contact, and 1% with water. Considering all of these sources, we chose 94% as the proportion foodborne.
**Comments**	Although also associated with exposure to reptiles, contaminated water, and other sources, non-typhoidal *Salmonella* is primarily a foodborne disease.

**Pathogen:** ***Salmonella enterica*** **serotype Typhi**	
**Estimate**	**Data source(s)**
**Number of illnesses**	Annual incidence of *Salmonella* serotype Typhi infection reported to CDC's Foodborne Diseases Active Surveillance Network (FoodNet) sites (2005-2008) (11); adjusted for geographical coverage (FoodNet is in 10 sites around the United States) and under-diagnosis resulting from the following surveillance steps: medical care seeking, specimen submission, laboratory testing, and test sensitivity (see online Technical Appendix 2).
**Number of hospitalizations**	Proportion of FoodNet cases of serotype Typhi infections hospitalized (2005–2008) applied the estimated number of reported cases (after adjusting for geographical coverage) and doubled to adjust for under-diagnosis.
**Number of deaths**	Proportion of FoodNet cases of serotype Typhi infections that died (2005–2008) applied the estimated number of reported cases (after adjusting for geographical coverage) and doubled to adjust for under-diagnosis.
**Proportion travel-related**	67% based on surveillance data from FoodNet (2005–2008). Cases of serotype Typhi infection in FoodNet were queried about international travel in the 30 days before illness began. Estimates were based on cases with known travel history.
**Proportion foodborne**	76% (13/17) of all domestically acquired outbreaks reported to the CDC between 1980 and 1999 were foodborne; 100% (13/13) of outbreaks with a known route of transmission (55).
**Comments**	Although waterborne and sexually transmitted outbreaks have been reported in the United States, foodborne transmission is believed to account for most cases.

Pathogen: Sapovirus	
**Estimate**	**Data source(s)**
**Number of illnesses**	We assumed that 75% of children experience an episode of clinical illness due to sapovirus by 5 years of age similar to other childhood gastroenteirits viruses such as rotavirus (2). The person-time at risk for 2006 was estimated as the 0-4 year population (20,417,636) divided by 5 and rounded (3).
**Number of hospitalizations**	Assumed to equal 25% of number of hospitalizations for rotavirus.
**Number of deaths**	Very low: 0-10 deaths per year.
**Proportion travel-related**	Assumed to be almost 100% domestically acquired since international travel-associated illness among children is likely small compared to the domestic burden.
**Proportion foodborne**	Significant illness assumed to occur only among children <5 years of age, although very occasionally foodborne; very few foodborne outbreaks reported (<1% of illnesses) (6).

**Pathogen: *Shigella* spp.**	
**Estimate**	**Data source(s)**
**Number of illnesses**	Annual incidence of shigellosis reported to CDC's Foodborne Diseases Active Surveillance Network (FoodNet) sites (2005-2008) (11); adjusted for geographical coverage (FoodNet is in 10 sites around the United States) and under-diagnosis resulting from the following surveillance steps: medical care seeking, specimen submission, laboratory testing, and test sensitivity (see online Technical Appendix 2).
**Number of hospitalizations**	Proportion of FoodNet cases of *Shigella* spp. infection hospitalized (2005–2008) applied the estimated number of reported cases (after adjusting for geographical coverage) and doubled to adjust for under-diagnosis.
**Number of deaths**	Proportion of FoodNet cases of *Shigella* spp. infection who died (2005–2008) applied the estimated number of reported cases (after adjusting for geographical coverage) and doubled to adjust for under-diagnosis.
**Proportion travel-related**	15% based on surveillance data from FoodNet (2005–2008). Cases of *Shigella* spp. infection in FoodNet were queried about international travel in the seven days before illness began. Estimates were based on cases with known travel history.
**Proportion foodborne**	31% based on 2005 FoodNet survey of risk factors for sporadic shigellosis. Persons who responded negatively to all risk-exposure questions were considered to represent an estimate of the proportion of sporadic shigellosis infections that may have been acquired through consumption of contaminated food in the United States (56).

**Pathogen:** ***Staphylococcus aureus***	
**Estimate**	**Data source(s)**
**Number of illnesses**	Annual number of *Staphylococcus aureus* outbreak-associated illnesses reported to CDC's Foodborne Disease Outbreak Surveillance System (2000–2007) (6) with an adjustment for trend (recent years were weighted more heavily) (see online Technical Appendix 3); adjusted for underreporting due to surveillance type (see outbreak surveillance underreporting multiplier described in online Technical Appendix 3 and 4) and under-diagnosis resulting from the following surveillance steps: medical care seeking, specimen submission, laboratory testing, and test sensitivity (see online Technical Appendix 2).
**Number of hospitalizations**	Proportion hospitalized in outbreaks reported to the Foodborne Disease Outbreak Surveillance System (2000–2007) applied to the estimated number of reported illnesses (after adjusting for underreporting) and doubled to adjust for under-diagnosis.
**Number of deaths**	Proportion who died in outbreaks reported to the Foodborne Disease Outbreak Surveillance System (2000–2007) applied to the estimated number of reported illnesses (after adjusting for underreporting) and doubled to adjust for under-diagnosis.
**Proportion travel-related**	Because of the rapid onset and short duration of illness, we assumed that 100% of *Staphylococcus aureus* illnesses occurring in the United States were domestically acquired.
**Proportion foodborne**	Our estimate of the number of illnesses was based on outbreak-associated *Staphylococcus aureus* illnesses reported to CDC through the Foodborne Disease Outbreak Surveillance System. Because all these outbreaks were foodborne, our estimate of the number of illnesses was based solely on foodborne outbreak-associated illnesses. Therefore, 100% of the estimated number of illnesses was considered foodborne.

**Pathogen: *Streptococcus* spp., Group A**	
**Estimate**	**Data source(s)**
**Number of illnesses**	Annual number of *Streptococcus* spp., Group A outbreak-associated illnesses reported to CDC's Foodborne Disease Outbreak Surveillance System (1996-2007) (6); adjusted for underreporting due to surveillance type (see outbreak surveillance underreporting multiplier described in online Technical Appendixes 2 and 4) and under-diagnosis resulting from the following surveillance steps: medical care seeking, specimen submission, laboratory testing, and test sensitivity (see online Technical Appendix 2). Data from 1996-2007 used because of a paucity of data.
**Number of hospitalizations**	Proportion hospitalized in *Streptococcus* spp., Group A outbreaks reported to the Foodborne Disease Outbreak Surveillance System (1981-2007) applied to the estimated number of reported illnesses (after adjusting for underreporting) and doubled to adjust for under-diagnosis. Data from 1981-2007, included 12 years when outbreaks occurred.
**Number of deaths**	Proportion who died in *Streptococcus* spp., Group A outbreaks reported to the Foodborne Disease Outbreak Surveillance System (1981-2007) applied to the estimated number of reported illnesses (after adjusting for underreporting) and doubled to adjust for under-diagnosis. Data from 1981-2007, included 12 years when outbreaks occurred.
**Proportion travel-related**	Because of the rapid onset and short duration of *Streptococcus* spp., Group A illnesses, we assumed that 100% of *Streptococcus* spp., Group A illnesses occurring in the United States are domestically acquired.
**Proportion foodborne**	Our estimate of the number of illnesses was based on outbreak-associated *Streptococcus* spp., Group A illnesses reported to CDC through the Foodborne Disease Outbreak Surveillance System. Because all these outbreaks were foodborne, our estimate of the number of illnesses was based solely on foodborne outbreak-associated illnesses. Therefore, 100% of the estimated number of illnesses was considered foodborne.

**Pathogen:** ***Toxoplasma gondii***	
**Estimate**	**Data source(s)**
**Number of illnesses**	Illnesses due to *Toxoplasma gondii* were estimated using nationally representative serologic data from the National Health and Nutrition Examination Survey (NHANES) (1999-2004) (57) and an estimate of seroconversion associated with clinical illness. The annual number of illnesses was modeled as the estimated symptomatic fraction of the estimated number of incident cases within the US population during a 1-year period. Incident cases were estimated using NHANES prevalence data. Specifically, the estimated prevalence for person aged 40-49 years reported in Jones *et al*. (57) was assumed to be the cumulative result of 45 years of constant incidence. The symptomatic fraction was estimated to be 15% (58).
**Number of hospitalizations**	Estimated based on national estimates of hospital discharge from the Healthcare Cost and Utilization Project (HCUP) Nationwide Inpatient Sample (NIS) (2000-2006) (25) using ICD-9-CM code 130 (Toxoplasmosis) and doubled to adjust for under-diagnosis.
**Number of deaths**	Estimated based on national estimates of inpatient deaths from NIS (2000-2006) using ICD-9-CM code 130 (Toxoplasmosis) and doubled to adjust for under-diagnosis.
**Proportion travel-related**	Assumed to be 100% domestically acquired.
**Proportion foodborne**	50% based on published studies (59, 60).
**Comments**	Although the proportion associated with eating contaminated food varies geographically, we assume an overall average of 50% (59, 60).

**Pathogen: *Trichinella* spp.**	
**Estimate**	**Data source(s)**
**Number of illnesses**	Annual number of cases of *Trichinella* spp. infection reported to CDC's National Notifiable Disease Surveillance System (NNDSS) (2000–2007) (7, 61); adjusted for underreporting due to surveillance type (see passive surveillance underreporting multiplier described in online Technical Appendixes 2 and 4) and under-diagnosis resulting from the following surveillance steps: medical care seeking, specimen submission, laboratory testing, and test sensitivity (see online Technical Appendix 2).
**Number of hospitalizations**	Proportion hospitalized in *Trichinella* spp. outbreaks reported to the Foodborne Disease Outbreak Surveillance System (2000–2007) (6) applied to the estimated number of reported illnesses (after adjusting for underreporting) and doubled to adjust for under-diagnosis.
**Number of deaths**	0.2% based on published study (62).
**Proportion travel-related**	4% based on surveillance data (61).
**Proportion foodborne**	Assumed to be 100% foodborne based on a published study (63).

**Pathogen: *Vibrio cholerae*, toxigenic**	
**Estimate**	**Data source(s)**
**Number of illnesses**	Annual number of toxigenic *Vibrio cholerae* illnesses reported to CDC's Cholera and Other *Vibrio* Illness Surveillance System (COVIS) (2000–2007) (64) with an adjustment for trend (recent years were weighted more heavily) (see online Technical Appendix 3; adjusted for underreporting due to surveillance type (see passive surveillance underreporting multiplier described in online Technical Appendixes 2 and 4) and under-diagnosis resulting from the following surveillance steps: medical care seeking, specimen submission, laboratory testing, and test sensitivity (see online Technical Appendix 2). Non-toxigenic *Vibrio cholerae* infections are included in "*Vibrio* spp., other".
**Number of hospitalizations**	Proportion of cases of toxigenic *Vibrio cholerae* infection reported to COVIS (2000-2007) who were hospitalized applied to the estimated number of reported illnesses (after adjusting for underreporting) and doubled to adjust for under-diagnosis.
**Number of deaths**	Proportion of cases of toxigenic *Vibrio cholerae* infection reported to COVIS (2000-2007) who died applied to the estimated number of reported illnesses (after adjusting for underreporting) and doubled to adjust for under-diagnosis.
**Proportion travel-related**	Proportion of travel-related cases reported to COVIS (2000–2007). Cases were queried about international travel before in the seven days before their illness began. Estimates were based on those with a known travel history.
**Proportion foodborne**	100% of these cases reported to COVIS (2000–2007) were classified as foodborne.
**Comments**	FoodNet conducts surveillance for *Vibrio* infections; however, because of the geographical clustering of cases of *Vibrio* infection in non-FoodNet states, CDC's passive Vibrio surveillance system, COVIS, was used.

**Pathogen:** ***Vibrio vulnificus***	
**Estimate**	**Data source(s)**
**Number of illnesses**	Annual number of *Vibrio vulnificus* illnesses reported to CDC's Cholera and Other *Vibrio* Illness Surveillance System (COVIS) (2000–2007) (64) with an adjustment for trend (recent years were weighted more heavily) (see online Technical Appendix 3); adjusted for underreporting due to surveillance type (see passive surveillance underreporting multiplier described in online Technical Appendixes 2 and 4) and under-diagnosis resulting from the following surveillance steps: medical care seeking, specimen submission, laboratory testing, and test sensitivity (see online Technical Appendix 2).
**Number of hospitalizations**	Proportion of cases of *Vibrio vulnificus* infection reported to COVIS (2000–2007) who were hospitalized applied to the estimated number of reported illnesses (after adjusting for underreporting) and doubled to adjust for under-diagnosis.
**Number of deaths**	Proportion of cases of *Vibrio vulnificus* infection reported to COVIS (2000–2007) who died applied to the estimated number of reported illnesses (after adjusting for underreporting) and doubled to adjust for under-diagnosis.
**Proportion travel-related**	Proportion of travel-related cases reported to COVIS (2000-2007). Cases were queried about international travel in the seven days before their illness began. Estimates were based on those with a known travel history.
**Proportion foodborne**	Proportion of cases of *Vibrio vulnificus* infection reported to COVIS (2000–2007) that were classified as foodborne.
**Comments**	FoodNet conducts surveillance for *Vibrio* infections; however, because of the geographical clustering of cases of *Vibrio* infection in non-FoodNet states, CDC's passive Vibrio surveillance system, COVIS, was used.

**Pathogen:** ***Vibrio parahaemolyticus***	
**Estimate**	**Data source(s)**
**Number of illnesses**	Annual number of *Vibrio parahaemolyticus* illnesses reported to CDC's Cholera and Other *Vibrio* Illness Surveillance System (COVIS) (2000–2007) (64) with an adjustment for trend (recent years were weighted more heavily) (see online Technical Appendix 3; adjusted for underreporting due to surveillance type (see passive surveillance underreporting multiplier described in online Technical Appendixes 2 and 4) and under-diagnosis resulting from the following surveillance steps: medical care seeking, specimen submission, laboratory testing, and test sensitivity (see online Technical Appendix 2).
**Number of hospitalizations**	Proportion of cases of *Vibrio parahaemolyticus* infection reported to COVIS (2000-2007) who were hospitalized applied to the estimated number of reported illnesses (after adjusting for underreporting) and doubled to adjust for under-diagnosis.
**Number of deaths**	Proportion of cases of *Vibrio parahaemolyticus* infection reported to COVIS (2000-2007) who died applied to the estimated number of reported illnesses (after adjusting for underreporting) and doubled to adjust for under-diagnosis.
**Proportion travel-related**	Proportion of travel-related cases reported to COVIS (2000–2007). Cases were queried about international travel before their illness began. Estimates were based on those with a known travel history.
**Proportion foodborne**	Proportion of *Vibrio parahaemolyticus* cases reported to COVIS (2000–2007) were classified as foodborne.
**Comments**	FoodNet conducts surveillance for *Vibrio* infections; however, because of the geographical clustering of cases of *Vibrio* infection in non-FoodNet states, CDC's passive Vibrio surveillance system, COVIS, was used.

**Pathogen: *Vibrio* spp., other**	
**Estimate**	**Data source(s)**
**Number of illnesses**	Annual number of *Vibrio* illnesses other than toxigenic *Vibrio cholerae, Vibrio vulnificus, and Vibrio parahaemolyticus* illnesses to CDC's Cholera and Other *Vibrio* Illness Surveillance System (COVIS) (2000-2007) (64) with an adjustment for trend (recent years were weighted more heavily) (see online Technical Appendix 3); adjusted for underreporting due to surveillance type (see passive surveillance underreporting multiplier described in online Technical Appendixes 2 and 4) and under-diagnosis resulting from the following surveillance steps: medical care seeking, specimen submission, laboratory testing, and test sensitivity (see online Technical Appendix 2).
**Number of hospitalizations**	Proportion of cases of *Vibrio* infection other than toxigenic *Vibrio cholerae, Vibrio vulnificus, and Vibrio parahaemolyticus* reported to COVIS (2000–2007) who were hospitalized applied to the estimated number of reported illnesses (after adjusting for underreporting) and doubled to adjust for under-diagnosis.
**Number of deaths**	Proportion of cases of *Vibrio* infection other than toxigenic *Vibrio cholerae, Vibrio vulnificus, and Vibrio parahaemolyticus* reported to COVIS (2000–2007) who died applied to the estimated number of reported illnesses (after adjusting for underreporting) and doubled to adjust for under-diagnosis.
**Proportion travel-related**	Proportion of travel-related cases reported to COVIS (2000-2007). Cases were queried about international travel before their illness began. Estimates were based on those with a known travel history.
**Proportion foodborne**	Proportion of *Vibrio* infection other than toxigenic *Vibrio cholerae, Vibrio vulnificus, and Vibrio parahaemolyticus* reported to COVIS (2000–2007) that were classified as foodborne.
**Comments**	FoodNet conducts surveillance for *Vibrio* infections; however, because of the geographical clustering of cases of *Vibrio* infection in non-FoodNet states, CDC's passive Vibrio surveillance system, COVIS, was used.

**Pathogen:** ***Yersinia enterocolitica***	
**Estimate**	**Data source(s)**
**Number of illnesses**	Annual incidence of *Yersinia enterocolitica* infection reported to CDC's Foodborne Diseases Active Surveillance Network (FoodNet) sites (2005-2008) (11); adjusted for geographical coverage (FoodNet is in 10 sites around the United States) and under-diagnosis resulting from the following surveillance steps: medical care seeking, specimen submission, laboratory testing, and test sensitivity (see online Technical Appendix 2.
**Number of hospitalizations**	Proportion of FoodNet cases of *Yersinia enterocolitica* infection hospitalized (2005-2008) applied the estimated number of reported cases (after adjusting for geographical coverage) and doubled to adjust for under-diagnosis.
**Number of deaths**	Proportion of FoodNet cases of *Yersinia enterocolitica* infection who died (2005-2008) applied the estimated number of reported cases (after adjusting for geographical coverage) and doubled to adjust for under-diagnosis.
**Proportion travel-related**	7% based on surveillance data from FoodNet (2005-2008). Cases of *Yersinia enterocolitica* infection in FoodNet were queried about international travel in the seven days before illness began. Estimates were based on cases with known travel history.
**Proportion foodborne**	We assumed that 90% of cases were foodborne based on limited data from published studies (65).
**Comments**	Nearly all reported outbreaks in United States have been linked to contaminated foods.

References

1. Kriston, S., Willcocks, M.M., Carter, M.J., and Cubitt, W.D. Seroprevalence of astrovirus types 1 and 6 in London, determined using recombinant virus antigen. Epidemiol Infect. 1996;117:159-64.
2. Widdowson, M.A., Meltzer, M.I., Zhang, X., Bresee, J.S., Parashar, U.D., and Glass, R.I. Cost-effectiveness and potential impact of rotavirus vaccination in the United States. Pediatrics. 2007;119:684-97.

3. US Census Bureau. Population Estimates. [cited 2010 March 9]; Available from: http://www.census.gov/popest/states/NST-ann-est.html.
4. Dennehy, P.H., Nelson, S.M., Spangenberger, S., Noel, J.S., Monroe, S.S., and Glass, R.I. A prospective case-control study of the role of astrovirus in acute diarrhea among hospitalized young children. J Infect Dis. 2001;184:10-5.
5. Glass, R.I., Noel, J., Mitchell, D., Herrmann, J.E., Blacklow, N.R., Pickering, L.K., et al. The changing epidemiology of astrovirus-associated gastroenteritis: a review. Arch Virol Suppl. 1996;12:287-300.
6. Centers for Disease Control and Prevention. Surveillance for foodborne disease outbreaks - United States, 2006. MMWR Morb Mortal Wkly Rep. 2009;58:609-15.
7. Nieves, E., Jajosky, R.A., Adams, D.A., Sharp, P., Anderson, W.J., Aponte, J.J., et al. Summary of notifiable diseases--United States, 2007. MMWR Morb Mortal Wkly Rep. 2009;56:1-94.
8. Taylor, J.P. and Perdue, J.N. The changing epidemiology of human brucellosis in Texas, 1977-1986. Am J Epidemiol. 1989;130:160-5.
9. Chomel, B.B., DeBess, E.E., Mangiamele, D.M., Reilly, K.F., Farver, T.B., Sun, R.K., et al. Changing trends in the epidemiology of human brucellosis in California from 1973 to 1992: a shift toward foodborne transmission. J Infect Dis. 1994;170:1216-23.
10. Mead, P.S., Slutsker, L., Dietz, V., McCaig, L.F., Bresee, J.S., Shapiro, C., et al. Food-related illness and death in the United States. Emerg Infect Dis. 1999;5:607-25.
11. Centers for Disease Control and Prevention. Preliminary FoodNet Data on the incidence of infection with pathogens transmitted commonly through food--10 States, 2008. MMWR Morb Mortal Wkly Rep. 2009;58:333-7.
12. Friedman, C.R., Hoekstra, R.M., Samuel, M., Marcus, R., Bender, J., Shiferaw, B., et al. Risk factors for sporadic *Campylobacter* infection in the United States: A case-control study in FoodNet sites. Clin Infect Dis. 2004;38 Suppl 3:S285-96.
13. St Louis, M.E., Peck, S.H., Bowering, D., Morgan, G.B., Blatherwick, J., Banerjee, S., et al. Botulism from chopped garlic: delayed recognition of a major outbreak. Ann Intern Med. 1988;108:363-8.
14. Sobel, J., Malavet, M., and John, S. Outbreak of clinically mild botulism type E illness from home-salted fish in patients presenting with predominantly gastrointestinal symptoms. Clin Infect Dis. 2007;45:e14-6.
15. Majowicz, S.E., Michel, P., Aramini, J.J., McEwen, S.A., and Wilson, J.B. Descriptive analysis of endemic cryptosporidiosis cases reported in Ontario, 1996-1997. Can J Public Health. 2001;92:62-6.
16. Herwaldt, B.L. *Cyclospora cayetanensis*: a review, focusing on the outbreaks of cyclosporiasis in the 1990s. Clin Infect Dis. 2000;31:1040-57.
17. Herwaldt, B. The ongoing saga of U.S. outbreaks of cyclosporiasis associated with imported fresh produce: what *Cyclospora cayetanensis* has taught us and what we have yet to learn, in Addressing foodborne threats to health: policies, practices, and global coordination. Coordination Workshop Summary, B.o.G.H. Forum on Microbial Threats, Institute of Medicine of the National Academies, Editor. 2006, The National Academies Press: Washington, DC. 85-115, 33-40
18. Rangel, J.M., Sparling, P.H., Crowe, C., Griffin, P.M., and Swerdlow, D.L. Epidemiology of *Escherichia coli* O157:H7 outbreaks, United States, 1982-2002. Emerg Infect Dis. 2005;11:603-9.

19. Centers for Disease Control and Prevention. Laboratory-confirmed non-O157 Shiga toxin-producing *Escherichia coli*--Connecticut, 2000-2005. MMWR Morb Mortal Wkly Rep. 2007;56:29-31.
20. Manning, S.D., Madera, R.T., Schneider, W., Dietrich, S.E., Khalife, W., Brown, W., et al. Surveillance for Shiga toxin-producing *Escherichia coli*, Michigan, 2001-2005. Emerg Infect Dis. 2007;13:318-21.
21. Hedican, E.B., Medus, C., Besser, J.M., Juni, B.A., Koziol, B., Taylor, C., et al. Characteristics of O157 versus non-O157 Shiga toxin-producing *Escherichia coli* infections in Minnesota, 2000-2006. Clin Infect Dis. 2009;49:358-64.
22. Centers for Disease Control and Prevention. Non-O157 Shiga toxin-producing E. coli (STEC) outbreaks, United States. Memorandum to the Record. 2010.
23. Cohen, M.B., Nataro, J.P., Bernstein, D.I., Hawkins, J., Roberts, N., and Staat, M.A. Prevalence of diarrheagenic *Escherichia coli* in acute childhood enteritis: a prospective controlled study. J Pediatr. 2005;146:54-61.
24. Nataro, J.P., Mai, V., Johnson, J., Blackwelder, W.C., Heimer, R., Tirrell, S., et al. Diarrheagenic *Escherichia coli* infection in Baltimore, Maryland, and New Haven, Connecticut. Clin Infect Dis. 2006;43:402-7.
25. Healthcare Cost and Utilization Project. National Inpatient Sample. [cited 2010 February 8]; Available from: http://www.hcup-us.ahrq.gov/nisoverview.jsp.
26. Chute, C.G., Smith, R.P., and Baron, J.A. Risk factors for endemic giardiasis. Am J Public Health. 1987;77:585-7.
27. Daniels, D., Grytdal, S., and Wasley, A. Surveillance for acute viral hepatitis - United States, 2007. MMWR Surveill Summ. 2009;58:1-27.
28. National Center for Health Statistics. Multiple Cause-of-Death Public-Use Data Files. [cited 2010 February 8]; Available from: http://www.cdc.gov/nchs/products/elec_prods/subject/mortmcd.htm.
29. Klevens, M., Miller, J., Iqbal, K., Thomas, A., Rocchio, E., Hanson, H., et al. The evolving epidemiology of Hepatitis A in the United States: Incidence and molecular epidemiology from population-based surveillance, 2005-2007. Arch Intern Med. In press.
30. Varma, J.K., Samuel, M.C., Marcus, R., Hoekstra, R.M., Medus, C., Segler, S., et al. *Listeria monocytogenes* infection from foods prepared in a commercial establishment: a case-control study of potential sources of sporadic illness in the United States. Clin Infect Dis. 2007;44:521-8.
31. Schwartz, B., Ciesielski, C.A., Broome, C.V., Gaventa, S., Brown, G.R., Gellin, B.G., et al. Association of sporadic listeriosis with consumption of uncooked hot dogs and undercooked chicken. Lancet. 1988;2:779-82.
32. Schuchat, A., Deaver, K.A., Wenger, J.D., Plikaytis, B.D., Mascola, L., Pinner, R.W., et al. Role of foods in sporadic listeriosis. I. Case-control study of dietary risk factors. The Listeria Study Group. JAMA. 1992;267:2041-5.
33. Pinner, R.W., Schuchat, A., Swaminathan, B., Hayes, P.S., Deaver, K.A., Weaver, R.E., et al. Role of foods in sporadic listeriosis. II. Microbiologic and epidemiologic investigation. The Listeria Study Group. JAMA. 1992;267:2046-50.
34. Riedo, F.X., Pinner, R.W., Tosca, M.L., Cartter, M.L., Graves, L.M., Reeves, M.W., et al. A point-source foodborne listeriosis outbreak: documented incubation period and possible mild illness. J Infect Dis. 1994;170:693-6.

35. Dalton, C.B., Austin, C.C., Sobel, J., Hayes, P.S., Bibb, W.F., Graves, L.M., et al. An outbreak of gastroenteritis and fever due to *Listeria monocytogenes* in milk. N Engl J Med. 1997;336:100-5.
36. Centers for Disease Control and Prevention. Reported Tuberculosis in the United States, 2007. 2008, Department of Health and Human Services, CDC: Atlanta, GA.
37. Hlavsa, M.C., Moonan, P.K., Cowan, L.S., Navin, T.R., Kammerer, J.S., Morlock, G.P., et al. Human tuberculosis due to *Mycobacterium bovis* in the United States, 1995-2005. Clin Infect Dis. 2008;47:168-75.
38. Taylor, Z., Marks, S.M., Rios Burrows, N.M., Weis, S.E., Stricof, R.L., and Miller, B. Causes and costs of hospitalization of tuberculosis patients in the United States. Int J Tuberc Lung Dis. 2000;4:931-9.
39. Centers for Disease Control and Prevention. Human tuberculosis caused by *Mycobacterium bovis*--New York City, 2001-2004. MMWR Morb Mortal Wkly Rep. 2005;54:605-8.
40. Rodwell, T.C., Moore, M., Moser, K.S., Brodine, S.K., and Strathdee, S.A. Tuberculosis from Mycobacterium bovis in binational communities, United States. Emerg Infect Dis. 2008;14:909-16.
41. de Wit, M.A., Koopmans, M.P., Kortbeek, L.M., Wannet, W.J., Vinje, J., van Leusden, F., et al. Sensor, a population-based cohort study on gastroenteritis in the Netherlands: incidence and etiology. Am J Epidemiol. 2001;154:666-74.
42. Wheeler, J.G., Sethi, D., Cowden, J.M., Wall, P.G., Rodrigues, L.C., Tompkins, D.S., et al. Study of infectious intestinal disease in England: rates in the community, presenting to general practice, and reported to national surveillance. The Infectious Intestinal Disease Study Executive. BMJ. 1999;318:1046-50.
43. Amar, C.F., East, C.L., Gray, J., Iturriza-Gomara, M., Maclure, E.A., and McLauchlin, J. Detection by PCR of eight groups of enteric pathogens in 4,627 faecal samples: re-examination of the English case-control Infectious Intestinal Disease Study (1993-1996). Eur J Clin Microbiol Infect Dis. 2007;26:311-23.
44. Marshall, J.A., Hellard, M.E., Sinclair, M.I., Fairley, C.K., Cox, B.J., Catton, M.G., et al. Incidence and characteristics of endemic Norwalk-like virus-associated gastroenteritis. J Med Virol. 2003;69:568-78.
45. Jones, T.F., McMillian, M.B., Scallan, E., Frenzen, P.D., Cronquist, A.B., Thomas, S., et al. A population-based estimate of the substantial burden of diarrhoeal disease in the United States; FoodNet, 1996-2003. Epidemiol Infect. 2007;135:293-301.
46. Patel, M.M., Widdowson, M.A., Glass, R.I., Akazawa, K., Vinje, J., and Parashar, U.D. Systematic literature review of role of noroviruses in sporadic gastroenteritis. Emerg Infect Dis. 2008;14:1224-31.
47. Jansen, A., Stark, K., Kunkel, J., Schreier, E., Ignatius, R., Liesenfeld, O., et al. Aetiology of community-acquired, acute gastroenteritis in hospitalised adults: a prospective cohort study. BMC Infect Dis. 2008;8:143.
48. National Center for Health Statistics. National Hospital Discharge Survey. [cited 2010 February 2]; Available from: http://www.cdc.gov/nchs/nhds.htm.
49. Dennison, C. and Pokras, R. Design and operation of the National Hospital Discharge Survey: 1988 redesign. Vital Health Stat 2000;1(39).
50. National Center for Health Statistics. Ambulatory Health Care Data. [cited 2010 February 8]; Available from: http://www.cdc.gov/nchs/ahcd/about_ahcd.htm.

51. Gangarosa, R.E., Glass, R.I., Lew, J.F., and Boring, J.R. Hospitalizations involving gastroenteritis in the United States, 1985: the special burden of the disease among the elderly. Am J Epidemiol. 1992;135:281-90.
52. Mounts, A.W., Holman, R.C., Clarke, M.J., Bresee, J.S., and Glass, R.I. Trends in hospitalizations associated with gastroenteritis among adults in the United States, 1979-1995. Epidemiol Infect. 1999;123:1-8.
53. Heron, M., Hoyert, D.L., Murphy, S.L., Xu, J., Kochanek, K.D., and Tejada-Vera, B. Deaths: Final Data for 2006. National Vital Statistics Reports; vol 57 no 14. National Center for Health Statistics. 2009: Hyattsville, Maryland.
54. Mermin, J., Hutwagner, L., Vugia, D., Shallow, S., Daily, P., Bender, J., et al. Reptiles, amphibians, and human *Salmonella* infection: a population-based, case-control study. Clin Infect Dis. 2004;38 Suppl 3:S253-61.
55. Olsen, S.J., Bleasdale, S.C., Magnano, A.R., Landrigan, C., Holland, B.H., Tauxe, R.V., et al. Outbreaks of typhoid fever in the United States, 1960-99. Epidemiol Infect. 2003;130:13-21.
56. Haley, C.C., Ong, K.L., Hedberg, K., Cieslak, P.R., Scallan, E., Marcus, R., et al. Risk factors for sporadic shigellosis, FoodNet 2005. Foodborne Pathog Dis. 2010;7:741-7.
57. Jones, J.L., Kruszon-Moran, D., Sanders-Lewis, K., and Wilson, M. *Toxoplasma gondii* infection in the United States, 1999-2004, decline from the prior decade. Am J Trop Med Hyg. 2007;77:405-10.
58. World Health Organization. Toxoplasmosis, in Technical Report Series, No. 431. 1969, World Health Organization: Geneva.
59. Lopez, A., Dietz, V.J., Wilson, M., Navin, T.R., and Jones, J.L. Preventing congenital toxoplasmosis. MMWR Recomm Rep. 2000;49:59-68.
60. Cook, A.J., Gilbert, R.E., Buffolano, W., Zufferey, J., Petersen, E., Jenum, P.A., et al. Sources of toxoplasma infection in pregnant women: European multicentre case-control study. European Research Network on Congenital Toxoplasmosis. BMJ. 2000;321:142-7.
61. Kennedy, E.D., Hall, R.L., Montgomery, S.P., Pyburn, D.G., and Jones, J.L. Trichinellosis surveillance - United States, 2002-2007. MMWR Surveill Summ. 2009;58:1-7.
62. Hennekeuser, H.H., Pabst, K., Poeplau, W., and Gerok, W. Zur Klinik und Therapie der Trichinose: Boebachtungen and 47 Patienten wahrend einer Epidemic. Disch Med Wochenschr 1968;93:867-73.
63. Capo, V. and Despommier, D.D. Clinical aspects of infection with *Trichinella* spp. Clin Microbiol Rev. 1996;9:47-54.
64. Centers for Disease Control and Prevention. Cholera and Other Vibrio Illness Surveillance System. [cited 2010 Jul 27]; Available from: http://www.cdc.gov/nationalsurveillance/cholera_vibrio_surveillance.html.
65. Ostroff, S. *Yersinia* as an emerging infection: epidemiologic aspects of yersiniosis. Contrib Microbiol Immunol. 1995;13:5-10.
