# Supplementary material for: Foodborne Illness Acquired in the United States—Major Pathogens
Source: Emerg Infect Dis. 2011 Jan;17(1):7–15. doi: 10.3201/eid1701.P11101 (PMC3375761; doi:10.3201/eid1701.P11101)
Supplement: Appendix — Model Structures Used to Make Estimates. [file 09-1101p1-Techapp2.pdf]

Article DOI: 10.3201/eid1701.P11101

Foodborne Illness Acquired in the United States—Major Pathogens

Technical Appendix 2

Model Structures Used to Make Estimates

Background

Our choices for model structures were derived from a viewpoint about how to combine basic data on counts of illness, hospitalizations, and deaths with objective and subjective knowledge of the processes that might link those data to the true burden of illnesses, hospitalizations, and deaths. The following explanation describes our general approach and philosophy, and some of the key choices we made in assigning distributions. We started with the basic observation that the process of estimating the burden of foodborne illness requires using many disparate data sources and making subjective decisions about how to combine them. Therefore, we considered it important to take account of both statistical and non-statistical uncertainty.

We chose the 4-parameter beta distribution as our basic descriptive distribution because it allowed us to specify a minimum, maximum, and modal value, as well a fourth parameter that controls the spread (variance) of the distribution within those limits. This family of distributions is widely used in problems of expert elicitation and risk assessment, particularly in the forms known as the PERT distribution and the Modified PERT distribution (*1*). (We use PERT to refer to both the PERT and Modified PERT distributions). Because of the intuitive nature of its inputs, it is an attractive choice for problems in which many estimates and sources of uncertainty need to be combined.

Naturally, much of the source data for our estimates was in the form of counts. We found that using standard parametric distributions, such as the Poisson and Negative Binomial families, to describe our count data generally masked important features, such as left-skewness and multimodality. We decided to use nonparametric descriptions instead, and extended that choice

to other data, such as observed proportions, as indicated in online Technical Appendix 3 (www.cdc.gov/EID/content/17/1/7-Techapp3.pdf). Typically, we chose to use empirical distributions in describing surveillance data and data that had features that we thought should not be smoothed via summary reduction; the 4-parameter beta was used in situations that incorporated multiple distinct subjective elements. For example, we often combined multiple values from published literature, and wanted to incorporate their reported and often unreported statistical uncertainties, and the non-statistical uncertainties associated with their differing data sources and methodologies. Our choice to preserve features of the data using empirical distributions is discussed further below.

Much of the data we used could have been treated as if they were derived from simple sampling models; in statistical terms, we could have assumed that observations were identically distributed and independent. For example, the FoodNet surveillance data for a given pathogen for 2005-2008 might be aggregated to annual counts and the resulting 4 counts treated as a random sample from the target population. The same approach might be applied to the annual outbreak-associated case counts, and the annual national surveillance case counts (online Technical Appendix 1, www.cdc.gov/EID/content/17/1/7-Techapp1.pdf). We could treat uncertainty in the usual statistical sense and operate, for example, with standard errors calculated as estimated population standard deviations divided by square roots of sample sizes.

We chose not to treat uncertainty in that way. The above approach makes two suspect assumptions. First, it assumes that the aggregated data represent a single sample of multiple counts from a homogeneous population (as opposed to a set of single count samples from distinct annual populations with different characteristics). This is the identically distributed part. Second, it assumes that the single sample is a random sample. This is the independently distributed part. While these assumptions might be approximately valid for some of our data, our historical experience with both our own and other surveillance and survey data, input from experts, and an examination of the data themselves, have convinced us that these assumptions are not likely to be valid in most cases. Moreover, we have no reliable way to distinguish those for which these assumptions are valid.

Therefore, we chose to treat, for example, an outbreak case count series of 8 years as representing 8 distinct population means which likely span the unknown mean of the target

population. This leads to an estimate that is still the mean of the observed data, but with an uncertainty described by the standard deviation of that data, and not a nominal standard error. In keeping with this idea that the data do not necessarily directly represent the characteristic being estimated, we chose to use empirical distributions as descriptions, thus preserving individual data points. We extended this approach beyond surveillance data to the population surveys and other data sources. Every data source indicated in Table 1 reflects multiple years of data collection, except for the Census data which identifies the target 2006 US resident population. [Even that has some visible uncertainty associated with it, in that one might argue to include or exclude non-resident or institutionalized or military populations, under specific circumstances.]

The outputs of our models are summarized by features of posterior distributions calculated by Monte Carlo simulation. While we were not able to perform a complete analysis of the uncertainty associated with the simulation itself (Monte Carlo error), the 100,000 replicate uniform basis for our distributions appears to generally achieve an error of less than 0.5% and frequently less than 0.1%, based on examining multiple simulations for non-typhoidal *Salmonella* and *Giardia*.

Model structures

We used different modeling structures, depending on data type, to estimate the total number of illnesses, hospitalizations, and deaths due to 31 known foodborne pathogens.

The model structures are of the following two broad types:

- Model type A: This model scales counts of laboratory-confirmed (reported) illnesses up to an estimated number of ill persons, accounting for underreporting and under-diagnosis factors that contribute to an illness not being reported to public health agencies (Box 1).
- Model type B: This model scales populations at risk down to an estimated number of ill persons (Box 2).

All models described here are multiplicative; successive factors are applied by multiplication to obtain proportional increases or decreases in the count. This tends to produce wider ranges in the final distribution estimates than additive models.

Each of these models has subtypes that reflect the available data. The figures describe mathematical multipliers in the key models. Figures 2, 2a, 3 and 5 consist of a series of histograms that describe the distributions of simulated individual multiplicative factors as they are successively applied to elements of the burden estimates. More details on the variations applied to these models are described in online Technical Appendix 3.

Multiplication of distributions is accomplished using Monte Carlo simulation. Simulation of the empirical distributions corresponds to simple nonparametric bootstrapping (*2*), which is the random re-sampling of observed data, with replacement, to obtain a series of new samples that simulate the variability in the original chance process that gave rise to the data. Bootstrapping provides the initial link to the approximate Bayesian interpretation of the model outputs (*3*,*4*).

Box 1: Pathogens for which laboratory-confirmed illnesses were scaled up to estimate the total number of illnesses

Active surveillance data	Passive surveillance data	Outbreak surveillance data
• *Campylobacter* spp.	• *Brucella* spp.	• *Bacillus cereus*
• *Cryptosporidium* spp.	• *Clostridium botulinum,* foodborne	• *Clostridium perfringens*
• *Cyclospora cayetanensis*	• *Giardia intestinalis*	• *E. coli*, enterotoxigenic (ETEC)*
• *Escherichia coli*, Shiga toxin–producing (STEC) O157	• Hepatitis A	• *Staphylococcus aureus*
• *E. coli*, Shiga toxin-producing (STEC), non-O157	• *Mycobacterium bovis*	• *Streptococcus* spp., Group A
• *Listeria monocytogenes*	• *Trichinella* spp.	
• *Salmonella*, non-typhoidal	• *Vibrio cholerae,* toxigenic	
• *Salmonella* serotype Typhi	• *Vibrio parahaemolyticus*	
• *Shigella* spp.	• *Vibrio vulnificus*	
• *Yersinia entercolitica*	• *Vibrio* spp., other	

***** *E. coli*, other than STEC or ETEC assumed to be equal to ETEC (online Technical Appendix 3)

Box 2: Pathogens for which populations were scaled down to estimate the total number of illnesses
- Astrovirus
- Norovirus
- Rotavirus
- Sapovirus
- *Toxoplasma gondii*

Figure 1 provides a schematic representation of the modeling process for pathogens for which reported counts of illness are scaled up. Some factors in the schematic are stochastic (*italic font*) and some factors are deterministic (**bold font**). Some factors are applied generally and some are applied as needed, depending on data source.

Note that the schematic shows 6 primary model outputs, as identified in the box at the right and obtained by inclusion of specific elements from the rightmost two model factors, column (1or H or D) and row (1 or F). For example, choice of D and F yields the output for

foodborne deaths. Each factor represents a probability distribution, either an empirical distribution based on observed or estimated data, or a parametric distribution. Details of the choices made to define these distributions are provided in online Technical Appendix 3. The model outputs are the resulting probability distributions from the multiplication of the component factor distributions. All factors for a given output are stochastically independent except for those making up the two-part mixture of mild and severe illness.

Figure 1: Schematic illustration of model type A, which scales case counts up

$$Count \times \begin{bmatrix} \textbf{Year} \\ \text{\& /or} \\ \textbf{Geo} \end{bmatrix} \times Dom \times Und \times Ob \times \begin{Bmatrix} CS(Severe) \times SS(Severe) \times PS \\ + \\ CS(Mild) \times SS(Mild) \times (1 - PS) \end{Bmatrix} \times LT \times LS \times \begin{bmatrix} \mathbf{1} \\ \text{or} \\ H \\ \text{or} \\ D \end{bmatrix} \times \overline{\underline{\mathbf{1} \ \text{ or } \ F}} \Rightarrow$$

Illnesses	*Foodborne Illnesses*
Hospitalizations	*Foodborne Hospitalizations*
Deaths	*Foodborne Deaths*

Where:

Count refers to data in the form of cases of reported illnesses.
Year is a deterministic factor to standardize non-2006 counts to 2006. Applied as needed
Geo is a deterministic expansive factor to scale FoodNet counts up to the entire US population. Applied as needed.
Dom is a contractive factor to scale total counts down to counts that are domestically acquired. Applied as needed.
Und is an expansive factor to scale passive surveillance case counts up to active surveillance counts. Applied as needed.
Ob is an expansive factor to scale outbreak case counts up to laboratory confirmed counts. Applied as needed.
CS is an expansive factor to scale care seekers up to all ill, with severe and mild illness versions.
SS is an expansive factor to scale submitted samples up to all ill visits, with severe and mild illness versions.
PS is the proportion of actual illness that is severe.
LT is an expansive factor to scale tests performed up to samples submitted.

LS is an expansive factor to scale positive tests up to true positive specimens.
H is a contractive factor to scale illnesses down to hospitalized illnesses.
D is a contractive factor to scale illnesses down to deaths.
F is a contractive factor to scale overall counts down to counts that are foodborne.

Figures 2, 2a, and 3 illustrate the stochastic model structures for *Campylobacter*, which provides an example of a pathogen for which reported cases are scaled up.

Figure 2: Model distributions for *Campylobacter* illnesses. The histogram of observed laboratory-confirmed illnesses reflects annual counts from each of the 10 sites in the FoodNet catchment from 2005 to 2008.

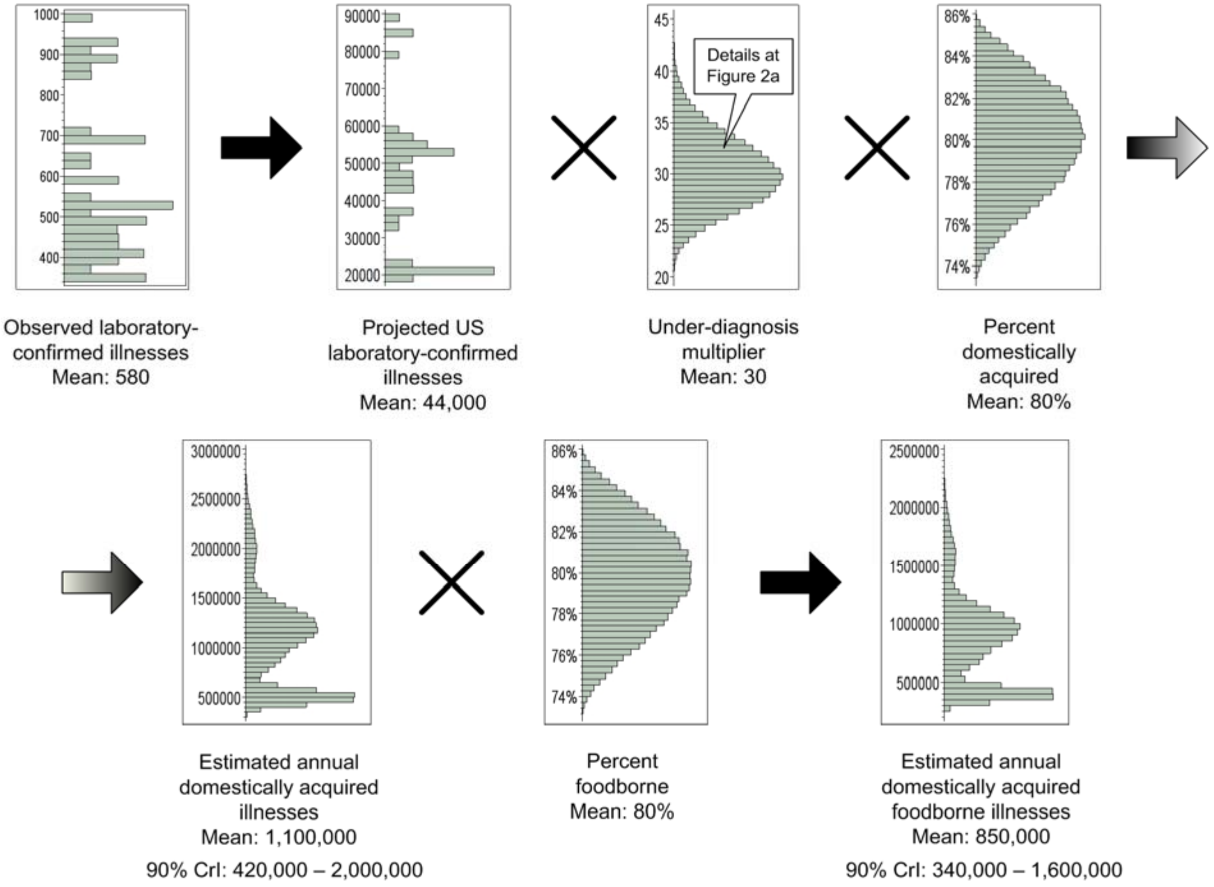

Figure 2a: Detailed structure of under-diagnosis multiplier for *Campylobacter* illnesses

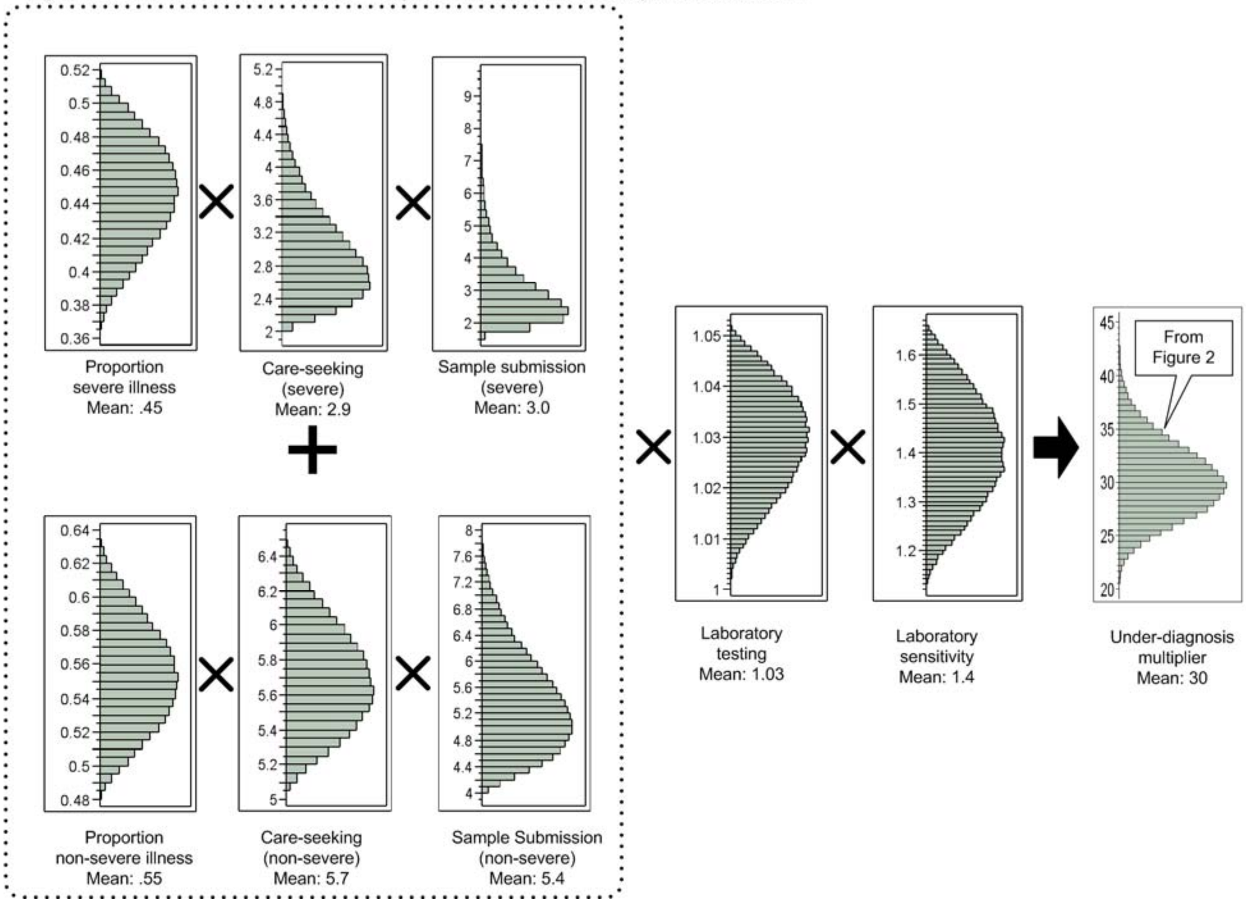

Figure 3: Model distributions for *Campylobacter* hospitalizations. Note that the first histogram shown is that of projected US laboratory-confirmed illnesses rather than observed laboratory-confirmed illnesses, as in Figure 2.

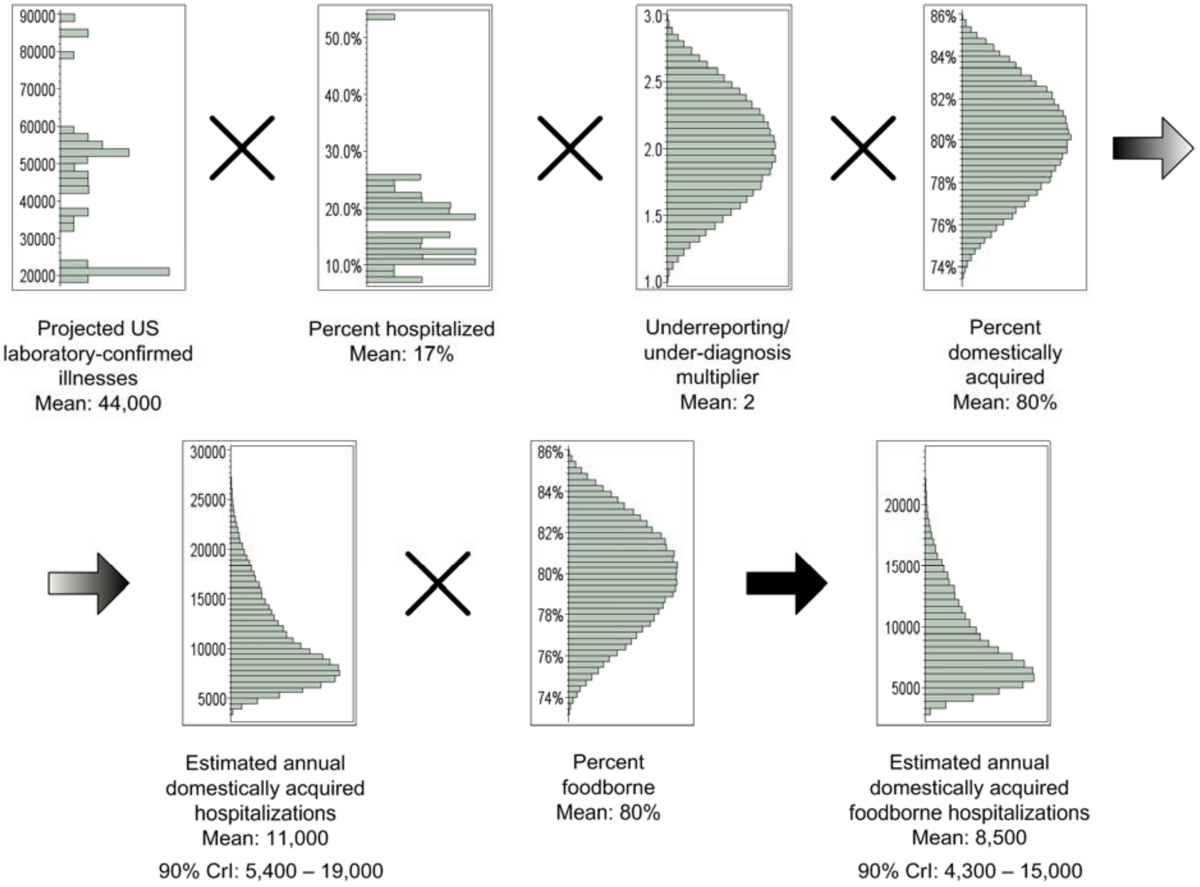

The empirical nature of the source data is apparent in the first panel of Figure 2, as is the parametric nature of the other factors. Note the complex multimodal nature of the output distribution of foodborne illnesses, a common feature among the pathogens whose burden is scaled up from reported cases. The best way to summarize the distribution is not obvious, e.g. mean, median, mode, or some more complex aggregating function of the data. We chose to summarize using the mean and the limits of a 90% credible interval (Tables 2 and 3 of the manuscript). The mode is obviously not possible. The mean is the most familiar measure and has the property that, under independence, the mean of the product is equal to the product of the means, which is not true of the median. That property makes the results of the analysis more transparent. We decided that by using both the mean and quantiles, we capture a broad picture of the distribution, applicable across a wide array of distributional shapes. Note that we treat the output distribution as a Bayesian posterior distribution. While our analysis is not fully Bayesian in that a full likelihood is not specified, bootstrapping of observed sample data has a

nonparametric Bayesian interpretation and many other elements of the models can be viewed as variously elicited prior distributions or even empirical Bayes posterior distributions. The other FoodNet pathogens from Box 1 were modeled in the same way. Each reported illness in the FoodNet data carries a field for whether the illness was outbreak-related, involved international travel, included hospitalization, and included death. Whether the illness occurred as part of a reported outbreak was determined for all FoodNet pathogens for 2004-2008. Travel status, hospitalization status, and death status were missing sufficiently often to warrant treatment, although some FoodNet sites had negligible missingness across most pathogens. Comparisons between FoodNet sites with differing levels of missingness suggested that an assumption of missingness at random (MAR) was reasonable, and so all three variables were treated so. That is, the status of each variable was predicted based on the relative proportions observed in cases for which that variable was not missing. Because missingness of travel status was high and variable, we used a PERT distribution based on overall known pathogen travel proportions and a generic uncertainty. For each pathogen's hospitalizations and deaths we predicted the value of missing status using the known proportions at the level of year and FoodNet site (i.e., the aggregation level of the FoodNet data chosen for all analyses), at each iteration of the bootstrap. (See Figure 3 panels for percent hospitalized and percent domestically acquired). We note two specific additional issues involving missingness. Six percent of *Salmonella* specimens were either not serotyped or only partially serotyped. We classified them as *Salmonella*, non-typhoidal because less than 1% of serotyped *Salmonella* were serotype Typhi. Relative to an MAR assumption, the potential bias from this decision would be expected to be on the order of 0.05%, negligible relative to the other sources of uncertainty in the *Salmonella* model. Eight percent of specimens of *Yersinia* were not speciated. We classified them as *enterocolitica* because only 9% of speciated *Yersinia* were other species. Relative to an MAR assumption, that potential bias would be expected to be on the order of 0.7%, negligible relative to the other sources of uncertainty in the *Yersinia* model.

Some different elements were used for passive surveillance and outbreak surveillance pathogens collectively and individually. Passive and outbreak surveillance pathogens were adjusted for underreporting, but in different ways (online Technical Appendix 4, www.cdc.gov/EID/content/17/1/7-Techapp4.pdf). Bootstrapping of the reported annual counts of Hepatitis A and each of the *Vibrio* categories was done on a weighted basis, where by weighted

we mean here that 2006 was treated as a distinct time point and bootstrapping was done ‘around’ this point. This was done because these pathogens showed apparent trends over the years 2000-2007. To account for this we fit simple linear regression lines to the data series and used the ordinate values of the fitted lines at year 2006 as the predicted mean counts. Bootstrapping was then performed on the regression residuals, scaled for the uncertainty of the linear fit, plus the constant predicted mean counts. This yielded a process that was very similar in terms of output distributions to the simple bootstrapping of the other pathogens. The data series were sufficiently short and patterns sufficiently simple that we did not consider more complicated trend models.

Figure 4 provides a schematic representation of the modeling process for pathogens for which populations at risk of illness were scaled down to estimate case counts. It is much simpler in basic form than the process for pathogens that are scaled up. Again, the schematic shows 6 primary model outputs, as identified in the box at the right and obtained by inclusion of specific elements from the rightmost two model factors, column (1 or H or D) and row (1 or F). Each stochastic factor represents a probability distribution. Details of the choices made to define these distributions are provided in online Technical Appendix 3. The model outputs are the resulting probability distributions from the multiplication of the component factor distributions. All factors for a given output are stochastically independent.

Figure 4: Schematic illustration of model structure for scaling populations down

$$Pop \times P_1 \times \cdots \times P_k \times Dom \times \begin{bmatrix} \mathbf{1} \\ \text{or} \\ H \\ \text{or} \\ D \end{bmatrix} \times \overline{\underline{\mathbf{1} \quad \text{or} \quad F}} \Rightarrow$$

Illnesses	*Foodborne Illnesses*
Hospitalizations	*Foodborne Hospitalizations*
Deaths	*Foodborne Deaths*

Where:

Pop refers to the particular population at risk of illness.

$P_1 – P_k$ is a generic set of contractive factors. (e.g., percent of episodes of AGI due to norovirus)

Dom is a contractive factor to scale total counts down to counts that are domestically acquired.

H is a contractive factor to scale illnesses down to hospitalized illnesses.

D is a contractive factor to scale illnesses down to deaths.
F is a contractive factor to scale overall counts down to counts that are foodborne.

Figure 5 (next page) illustrates model structures for norovirus, which provides an example of a pathogen for which populations at risk are scaled down. The only new element of the norovirus model relative to *Campylobacter* is reflected in the “Annual Incidence of AGI” panel. Multiple cycles of the FoodNet Population Survey were used to estimate monthly prevalence and in turn annual incidence of acute gastrointestinal illness (AGI). The survey data showed variation among the three surveys and by FoodNet site, with site being the largest source of variation. We computed estimates of site-level AGI incidence for the 10 sites contributing data across the three surveys. These estimates were bootstrapped and a normal error component was added, based on the standard errors of the site-level AGI estimates. The net effect was a “mixture of normal distributions” uncertainty model for AGI incidence. This is reflected in the colored segments of the Figure 5 panel. They combine to show a composition of densities. That is, at each value of incidence, the height of the density is partitioned into segments whose relative lengths reflect the conditional probability that a given site contributed the incidence value across the bootstrap replications. The ordering of the segments is from smallest site AGI incidence to largest site AGI incidence, and shows the ‘smearing’ of the distribution due to site-to-site variation.

Figure 5: Model distributions for norovirus illnesses. The size of the US population is modeled as a constant. "M" denotes millions. "AGI" denotes acute gastrointestinal illness.

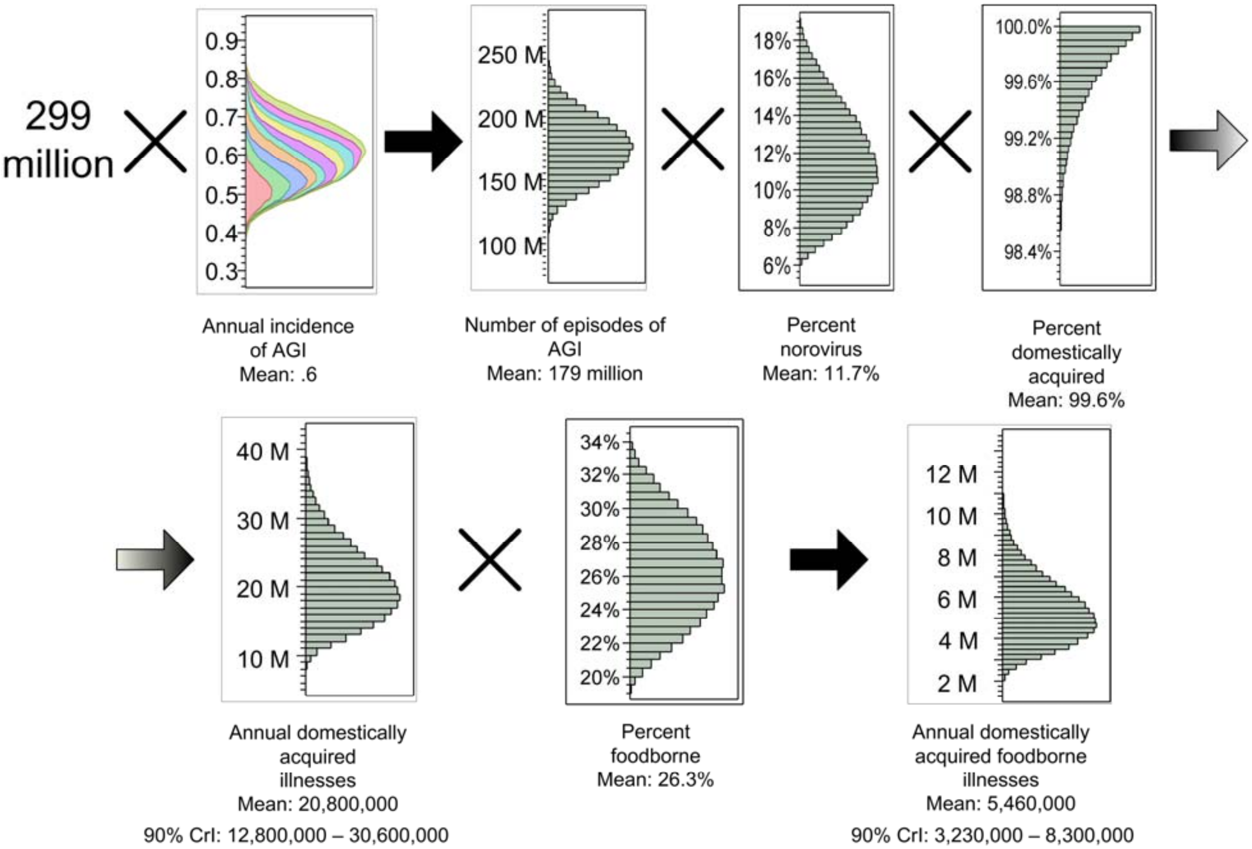

The remaining "scale down" pathogen models were constructed in a very similar fashion. Astrovirus, rotavirus, and sapovirus models were simpler than norovirus, with the chief distinction being that they all start from a population at risk defined to be the birth cohort for 2006. We simply applied distributions for the fractions that become infected, develop symptomatic illness, become hospitalized, and die. The *Toxoplasma gondii* model is based on a mathematical incidence model applied to the US population as a whole, and has complex uncertainties associated with applying such an incidence model across an entire population when the incidence dynamic has changed over time and the serology data that forms the basis of the model is cross-sectional. That said, the structure of the uncertainty distribution is not different from that of the other pathogens.

Final comments

We mentioned in the background section that we did not assume observed counts or ratios were identically distributed and independent. We did use means as best estimates, but retained the individual observations and their associated variability. It is worth noting that the retention of source data variability generally means that source data is the dominant component of the variance in the posterior distributions. This means that, in our models, widths of credible intervals are robust to the specification of *variability* for model elements such as underreporting and the components of under-diagnosis, percent domestically acquired, and percent foodborne. That robustness is particularly desirable because the total number of parameters to be specified is very large and the amount of pathogen-specific data is relatively small. Specifying a large number of distinct values, some based on subjective judgments and sparse data, is not desirable in the same sense that over-fitting of regression models is not desirable. The robustness of the model allowed us to use some common specifications. For example, we used laboratory test sensitivity inputs based on data for *Salmonella* to describe features relating to some other pathogens because pathogen-specific data were not available. This choice had little effect on the overall result for any pathogen.

Consider the last (lower right) panel of Figure 2. This panel shows a histogram that reflects our beliefs about the burden of annual domestically acquired foodborne illness from *Campylobacter* infection. It is distinctly multimodal and has a large coefficient of variation. An assumption that the FoodNet sites are a random sample of the United States as a whole would allow this distribution to be smoothed, and produce a unimodal posterior distribution, still with a mean of 850,000 but with a much smaller coefficient of variation. But because FoodNet is not a random sample and shows a very large degree of geographical variation in infection rates it is quite possible that the country as whole looks more like the three states that produced the lowest mode, Georgia, Maryland, and Tennessee, than the other seven sites under surveillance. We think that this is the most defensible presentation of burden uncertainty as derived from the available surveillance data. It explicitly acknowledges a substantial component of non-statistical uncertainty in the modeling process.

References

1. Vose, D., Risk analysis. A quantitative guide. 2nd ed. 2000: Chichester: John Wiley & Sons, LTD.
2. Efron, B. and Tibshirani, R.J., An Introduction to the Bootstrap. CRC Series: Monographs on Statistics & Applied Probability. 1993: Chapman & Hall/CRC Press
3. Rubin, D.B. The Bayesian Bootstrap. The Annals of Statistics. 1981;9:130-4.
4. Dalal, S. R. Fowlkes, E. B., Hoadley B., Risk Analysis of the Space Shuttle: Pre-Challenger Prediction of Failure. Journal of the American Statistical Association,1989;84:945- 957
