# Supplementary material for: Foodborne Illness Acquired in the United States—Major Pathogens
Source: Emerg Infect Dis. 2011 Jan;17(1):7–15. doi: 10.3201/eid1701.P11101 (PMC3375761; doi:10.3201/eid1701.P11101)
Supplement: Appendix — Estimation and Uncertainty Model Inputs for 31 Major Known Pathogens Transmitted Through Food. [file 09-1101p1-Techapp3.pdf]

Article DOI: 10.3201/eid1701.P11101

Foodborne Illness Acquired in the United States—Major Pathogens

Technical Appendix 3

Estimation and Uncertainty Model Inputs for 31 Major Known Pathogens Transmitted Through Food

**Pathogen: Astrovirus**			
**Model input**	**Data source(s)**	**Distribution**	**Parameters**
Person-time at risk	The person-time at risk for 2006 was estimated as the 0-4 year population (20,417,636) divided by 5 and rounded (1).	Constant	4,123,000
Proportion ill	75% of children assumed to experience an episode of clinical illness due to astrovirus by 5 years of age based on studies of rotavirus (2). Uncertainty with this proportion was based on a 50% relative increase/decrease from 0.75 on an odds scale.	Uniform	Low, high values: 0.55, 0.95
Proportion hospitalized	Hospitalization rate estimated as 25% of rotavirus using data from a published study (3).	PERT	Low, modal, high values: 0.003, 0.004, 0.006
Proportion who died	Very low: <10 deaths per year.	Uniform	Low, high values: 0.000, 0.0000024
Proportion travel-related	Assumed to be 100% domestically acquired.	PERT	Low, modal, high values: 0.000, 0.000, 0.001
Proportion foodborne	Very low (<1%) based on published review (4).	PERT	Low, modal, high values: 0.000, 0.005, 0.010

Pathogen: *Bacillus cereus*

**Model input**	**Data source(s)**	**Distribution**	**Parameters**
Reported illnesses	Number of *Bacillus cereus* outbreak-associated illnesses reported to CDC's Foodborne Disease Outbreak Surveillance System (2000-2007) (5).	Empirical	By year (2000-2007):<br>64, 76, 104, 85, 131, 69, 35, 100
Population adjustment (year)	Population ratios applied to each year from 2000-2007 based on US Census population estimates (1).	Degenerate	Ratio by year (2000-2007):<br>1.058, 1.047, 1.038, 1.029, 1.019, 1.010, 1.000, 0.990
Underreporting	Outbreak surveillance multiplier used to adjust for underreporting (see online Technical Appendix 4, www.cdc.gov/EID/content/17/1/7-Techapp4.pdf).	PERT	Low, modal, high, [precision] values:<br>5,16, 237, [20]
Proportion severe	Non-typhoidal *Salmonella* under-diagnosis multiplier used because of a lack of data on under-diagnosis factors. See Table 3.5 in this online Technical Appendix.		
Medical care seeking (severe)			
Medical care seeking (mild)			
Specimen submission (severe)			
Specimen submission (mild)			
Laboratory testing			
Test sensitivity			
Proportion hospitalized	Proportion of cases hospitalized in *Bacillus cereus* outbreaks reported to the Foodborne Disease Outbreak Surveillance System (2000-2007).	Empirical	By year (2000-2007):<br>0.016, 0.000, 0.000, 0.000, 0.080, 0.000, 0.000, 0.010
Proportion who died	Proportion of cases who died in *Bacillus cereus* outbreaks reported to the Foodborne Disease Outbreak Surveillance System (2000-2007).	Empirical	By year (2000-2007):<br>0.000, 0.000, 0.000, 0.000, 0.000, 0.000, 0.000, 0.000
Under-diagnosis (hospitalizations, deaths)	Number of hospitalizations and deaths doubled to account for under-diagnosis.	PERT	Low, modal, high values:<br>1, 2, 3

Proportion travel-related	Because of the rapid onset and short duration of *Bacillus cereus* illnesses, we assumed that almost 100% of *Bacillus cereus* illnesses occurring in the United States are domestically acquired.	PERT	Low, modal, high values: 0.00, 0.00, 0.02
Proportion foodborne	Estimates based on outbreak-associated illnesses from foodborne outbreaks reported to the Foodborne Disease Outbreak Surveillance System, therefore, estimated illnesses assumed to be 100% foodborne.	PERT	Low, modal, high values: 0.999, 1.000, 1.000

**Pathogen: *Brucella* spp.**			
**Model input**	**Data source(s)**	**Distribution**	**Parameters**
Reported illnesses	Number of illnesses caused by *Brucella* spp. reported to CDC's National Notifiable Disease Surveillance System (NNDSS) (2000-2007) (6).	Empirical	By year (2000-2007):<br>87, 136, 125, 104, 114, 120, 121, 131
Population adjustment (year)	Population ratios applied to each year from 2000-2007 based on US Census population estimates (1).	Degenerate	Ratio by year (2000-2007):<br>1.058, 1.047, 1.038, 1.029, 1.019, 1.010, 1.000, 0.990
Underreporting	Passive surveillance multiplier used to adjust for underreporting (see online Technical Appendix 4).	PERT	Low, modal, high values:<br>0.9, 1.1, 1.3
Proportion severe	Assumed to be 80% severe. Uncertainty with this proportion was based on a 50% relative increase/decrease from 0.80 on an odds scale.	PERT	Low, modal, high values:<br>0.73, 0.80, 0.86
Medical care seeking (severe)	Proportion (and 95% confidence interval (CI)) of survey respondents with bloody diarrhea who sought medical care from FoodNet Population Surveys (2000-2001, 2002-2003, 2006-2007) used as a proxy for severe illness (CDC, unpublished data).	PERT	Low, modal, high values:<br>0.19, 0.35, 0.51
Medical care seeking (mild)	Proportion (and 95% CI) of survey respondents with a non-bloody diarrhea who sought medical care from FoodNet Population Surveys (2000-2001, 2002-2003, 2006-2007) used as proxy for mild illness (CDC, unpublished data).	PERT	Low, modal, high values:<br>0.15, 0.18, 0.20

Specimen submission (severe)	Proportion (and 95% CI) of survey respondents who submitted a stool specimen among persons with bloody diarrhea who sought medical care from FoodNet Population Surveys (2000-2001, 2002-2003, 2006-2007) used as proxy for severe illness (CDC, unpublished data).	PERT	Low, modal, high values: 0.11, 0.36, 0.62
Specimen submission (mild)	Proportion (and 95% CI) of survey respondents who submitted a stool specimen among persons with a non-bloody diarrhea who sought medical care from FoodNet Population Surveys (2000-2001, 2002-2003, 2006-2007) used as a proxy for mild illness (CDC, unpublished data).	PERT	Low, modal, high values: 0.12, 0.19, 0.25
Laboratory testing	We assumed that most persons with brucellosis who submitted a specimen for testing would be tested for brucellosis.	PERT	Low, modal, high values: 0.94, 0.97, 1
Test sensitivity	Laboratory test sensitivity estimated to be between 85-95% based on blood culture (7).	PERT	Low, modal, high values: 0.85, 0.90, 0.95
Proportion hospitalized	55% of cases hospitalized in outbreaks reported to the CDC (CDC, unpublished data). Uncertainty with this proportion was based on a 50% relative increase/decrease from 0.55 on an odds scale.	PERT	Low, modal, high values: 0.45, 0.55, 0.65
Proportion who died	0.9% based on studies in Texas and California (8, 9). Uncertainty with this proportion was based on a 50% relative increase/decrease from 0.009 on an odds scale.	PERT	Low, modal, high values: , 0.006, 0.009, 0.013
Under-diagnosis (hospitalizations, deaths)	Number of hospitalizations and deaths doubled to account for under-diagnosis.	PERT	Low, modal, high values: 1, 2, 3
Proportion travel-related	16% of cases estimated to have acquired their infection outside the United States from NNDSS (2000-2007). Uncertainty with this proportion was based on a 50% relative increase/decrease from 0.16 on an odds scale.	PERT	Low, modal, high values: 0.11, 0.16, 0.22
Proportion foodborne	50% estimated based on published studies (9). Uncertainty with this proportion was based on a 50% relative increase/decrease from 0.50 on an odds scale.	PERT	Low, modal, high values: 0.40, 0.50, 0.60

**Pathogen: *Campylobacter* spp.**			
**Model input**	**Data source(s)**	**Distribution**	**Parameters**
Reported/projected US illnesses	Number of illnesses caused by *Campylobacter* spp. infection reported to CDC's Foodborne Diseases Active Surveillance Network (FoodNet) by FoodNet site (n=10) and year (2005-2008) (10).	Empirical	By site and year (2005-2008) see Tables 3.1 and 3.2 in this online Technical Appendix
Population adjustment (year)	Incidence of *Campylobacter* infection in each FoodNet site by year applied to 2006 US Census population estimates (1).	Degenerate	Adjustment by year (2005-2008): 1.010, 1.000, 0.990, 0.981
Underreporting	No underreporting multiplier; we assumed that all laboratory-confirmed *Campylobacter* illnesses were enumerated by FoodNet active surveillance.	-	-
Proportion severe	Proportion of cases by site reporting bloody diarrhea from FoodNet case-control study of sporadic laboratory-confirmed *Campylobacter* infections (11). We used uniform minimum variance unbiased (UMVU) estimators for lower and upper endpoints.	PERT	Low, modal, high values 0.36, 0.45, 0.52
Medical care seeking (severe)	Proportion (and 95% confidence interval (CI)) of survey respondents with bloody diarrhea who sought medical care from FoodNet Population Surveys (2000-2001, 2002-2003, 2006-2007) (CDC, unpublished data).	PERT	Low, modal, high values: 0.19, 0.35, 0.51
Medical care seeking (mild)	Proportion (and 95% CI) of survey respondents with a non-bloody diarrhea who sought medical care from FoodNet Population Surveys (2000-2001, 2002-2003, 2006-2007) (CDC, unpublished data).	PERT	Low, modal, high values: 0.15, 0.18, 0.20
Specimen submission (severe)	Proportion (and 95% CI) of survey respondents who submitted a stool specimen among persons with bloody diarrhea who sought medical care from FoodNet Population Surveys (2000-2001, 2002-2003, 2006-2007) (CDC, unpublished data).	PERT	Low, modal, high values: 0.11, 0.36, 0.62
Specimen submission (mild)	Proportion (and 95% CI) of survey respondents who submitted a stool specimen among persons with a non-bloody diarrhea who sought medical care from FoodNet Population Surveys (2000-2001, 2002-2003, 2006-2007) (CDC, unpublished data).	PERT	Low, modal, high values: 0.12, 0.19, 0.25

Laboratory testing	Proportion of clinical laboratories routinely testing stool samples for *Campylobacter* from the FoodNet Laboratory Survey (12). Uncertainty with this proportion (97%) was based on a 50% relative increase/decrease from 0.97 on an odds scale.	PERT	Low, modal, high values: 0.94, 0.97, 1.00
Test sensitivity	We used a laboratory test sensitivity rate of 70% based on studies of *Salmonella* (13, 14). We assumed a lower bound of 60% and an upper bound of 90%.	PERT	Low, modal, high values: 0.60, 0.70, 0.90
Proportion hospitalized	Proportion of FoodNet cases of *Campylobacter* infection who were hospitalized (2005-2008).	Empirical	By site and year (2005-2008) see Table 3.3 in this online Technical Appendix
Proportion who died	Proportion of FoodNet cases of *Campylobacter* infection who died (2005-2008).	Empirical	By site and year (2005-2008) see Table 3.4 in this online Technical Appendix
Under-diagnosis (hospitalizations, deaths)	Number of hospitalizations and deaths doubled to account for under-diagnosis.	PERT	Low, modal, high values: 1, 2, 3
Proportion travel-related	Proportion of FoodNet cases of *Campylobacter* infection who reported travel outside the United States within 7 days of illness onset (2005-2008). Uncertainty with this proportion (20%) was based on a 50% relative increase/decrease from 0.20 on an odds scale.	PERT	Low, modal, high values: 0.14, 0.20, 0.27
Proportion foodborne	1 – total non-foodborne population attributable fractions from FoodNet case-control study (11). Uncertainty with this proportion (80%) was based on a 50% relative increase/decrease from 0.80 on an odds scale.	PERT	Low, modal, high values: 0.73, 0.80, 0.86

**Pathogen:** ***Clostridium botulinum***			
**Model input**	**Data source(s)**	**Distribution**	**Parameters**
Reported illnesses	Number of foodborne botulism illnesses reported to CDC's National Notifiable Diseases Surveillance System (NNDSS) (2000-2007) (6).	Empirical	By year (2000-2007):<br>23, 39, 28, 20, 16, 19, 20, 32
Population adjustment (year)	Population ratios applied to each year from 2000-2007 based on US Census population estimates (1).	Degenerate	Ratios by year (2000-2007):<br>1.058, 1.047, 1.038, 1.029, 1.019, 1.010, 1.000, 0.990
Underreporting	Passive surveillance multiplier used to adjust for underreporting (see online Technical Appendix 4).	PERT	Low, modal, high values:<br>0.9, 1.1, 1.3
Proportion severe	Almost all cases of foodborne botulism assumed to be severe. Most cases of foodborne botulism reported to CDC's botulism surveillance are persons hospitalized for life-threatening manifestations.	PERT	Low, modal, high values:<br>0.95, 1.00, 1.00
Medical care seeking (severe)	Assumed to have a high rate of medical care seeking.	PERT	Low, modal, high values:<br>0.80, 0.90, 1.00
Medical care seeking (mild)	Assumed to have a high rate of medical care seeking.	PERT	Low, modal, high values:<br>0.80, 0.90, 1.00
Specimen submission (severe)	Assumed to have a high rate of specimen submission.	PERT	Low, modal, high values:<br>0.70, 0.80, 0.90
Specimen submission (mild)	Assumed to have a high rate of specimen submission.	PERT	Low, modal, high values:<br>0.70, 0.80, 0.90
Laboratory testing	Because persons hospitalized with botulism are often misdiagnosed with other serious illnesses, including Guillain-Barre syndrome and stroke, we assumed that only 70% would be tested appropriately for botulism (15).	PERT	Low, modal, high values:<br>0.61, 0.70, 0.78
Test sensitivity	Test sensitivity is 67% based on a published study (16); however, our estimates are based on counts that include epidemiologically linked cases that were not confirmed by a laboratory test but were part of recognized outbreaks.	PERT	Low, modal, high values:<br>0.999, 1.000, 1.000
Proportion hospitalized	Proportion of cases hospitalized in foodborne botulism outbreaks reported to the Foodborne Disease Outbreak Surveillance System (2000-2007) (5).	Empirical	By year (2000-2007):<br>1.000, 0.591, 0.643, 1.000, 1.000, 0.600, 0.769, 1.000

Proportion who died	Proportion of cases who died in foodborne botulism outbreaks reported to the Foodborne Disease Outbreak Surveillance System (2000-2007).	Empirical	By year (2000-2007): 0.200, 0.000, 0.000, 1.000, 0.000, 0.100, 0.077, 0.000
Under-diagnosis (hospitalizations, deaths)	Number of hospitalizations and deaths doubled to account for under-diagnosis.	PERT	Low, modal, high values: 1, 2, 3
Proportion travel-related	Almost all cases reported to CDC's botulism surveillance were domestically acquired, proportion of travel-related cases assumed to be very low.	PERT	Low, modal, high values: 0.00, 0.00, 0.02
Proportion foodborne	Illnesses reported to NNDSS as foodborne botulism, therefore, assumed to be 100% foodborne.	PERT	Low, modal, high values: 0.999, 1.000, 1.000

**Pathogen: *Clostridium perfingens***			
**Model input**	**Data source(s)**	**Distribution**	**Parameters**
Reported illnesses	Number of *Clostridium perfingens* outbreak-associated illnesses reported to CDC's Foodborne Disease Outbreak Surveillance System (2000-2007) (5).	Empirical	By year (2000-2007): 802, 1235, 2243, 2070, 1276, 416, 732, 1334
Population adjustment (year)	Population ratios applied to each year from 2000-2007 based on US Census population estimates (1).	Degenerate	Ratios by year (2000-2007): 1.058, 1.047, 1.038, 1.029, 1.019, 1.010, 1.000, 0.990
Underreporting	Outbreak surveillance multiplier used to adjust for underreporting (see online Technical Appendix 4)	PERT	Low, modal, high, [precision] values: 5,16, 237, [20]

Proportion severe	Non-typhoidal *Salmonella* under-diagnosis multiplier used because of a lack of data on under-diagnosis factors. See Table 3.5 in this online Technical Appendix.		
Medical care seeking (severe)			
Medical care seeking (mild)			
Specimen submission (severe)			
Specimen submission (mild)			
Laboratory testing			
Test sensitivity			
Proportion hospitalized	Proportion of cases hospitalized in *Clostridium perfingens* outbreaks reported to the Foodborne Disease Outbreak Surveillance System (2000-2007).	PERT	By year (2000-2007): 0.002, 0.006, 0.001, 0.018, 0.004, 0.007, 0.003, 0.005
Proportion who died	Proportion of cases who died in foodborne *Clostridium perfingens* outbreaks reported to the Foodborne Disease Outbreak Surveillance System (2000-2007).	PERT	By year (2000-2007): 0.000, 0.002, 0.000, 0.000, 0.001, 0.000, 0.000, 0.000
Under-diagnosis (hospitalizations, deaths)	Number of hospitalizations and deaths doubled to account for under-diagnosis.	PERT	Low, modal, high values: 1, 2, 3
Proportion travel-related	Because of the rapid onset and short duration of illness caused by *Clostridium perfingens*, we assumed that almost 100% of illnesses occurring in the United States are domestically acquired.	PERT	Low, modal, high values: 0.00, 0.00, 0.02
Proportion foodborne	Estimates based on outbreak-associated illnesses from foodborne outbreaks reported to the Foodborne Disease Outbreak Surveillance System, therefore, estimated illnesses assumed to be 100% foodborne.	PERT	Low, modal, high values: 0.999, 1.000, 1.000

**Pathogen: *Cryptosporidium* spp.**			
**Model input**	**Data source(s)**	**Distribution**	**Parameters**
Reported illnesses	Incidence of illnesses due to *Cryptosporidium* spp. infection reported to CDC's Foodborne Diseases Active Surveillance Network (FoodNet) by FoodNet site (n=10) and year (2005-2008) (10).	Empirical	By site and year (2005-2008) see Tables 3.1 and 3.2 in this online Technical Appendix
Population adjustment (year)	Incidence of *Cryptosporidium* spp. in each FoodNet site by year applied to 2006 US Census population estimates (1).	Degenerate	Adjustment by year (2005-2008): 1.010, 1.000, 0.990, 0.981
Underreporting	No underreporting multiplier, we assumed that all laboratory-confirmed *Cryptosporidium* spp. illnesses were enumerated by FoodNet active surveillance.	-	-
Percent severe	Assumed to be mostly mild (17).	PERT	Low, modal, high values: 0.0, 0.0, 0.05
Medical care seeking (severe)	Proportion (and 95% confidence interval (CI)) of survey respondents with bloody diarrhea who sought medical care from FoodNet Population Surveys (2000-2001, 2002-2003, 2006-2007) (CDC, unpublished data).	PERT	Low, modal, high values: 0.19, 0.35, 0.51
Medical care seeking (mild)	Proportion (and 95% CI) of survey respondents with a non-bloody diarrhea who sought medical care from FoodNet Population Surveys (2000-2001, 2002-2003, 2006-2007) (CDC, unpublished data).	PERT	Low, modal, high values: 0.15, 0.18, 0.20
Specimen submission (severe)	Proportion (and 95% CI) of survey respondents who submitted a stool specimen among persons with bloody diarrhea who sought medical care from FoodNet Population Surveys (2000-2001, 2002-2003, 2006-2007) (CDC, unpublished data).	PERT	Low, modal, high values: 0.11, 0.36, 0.62
Specimen submission (mild)	Proportion (and 95% CI) of survey respondents who submitted a stool specimen among persons with a non-bloody diarrhea who sought medical care from FoodNet Population Surveys (2000-2001, 2002-2003, 2006-2007) (CDC, unpublished data).	PERT	Low, modal, high values: 0.12, 0.19, 0.25
Laboratory testing	Proportion of clinical laboratories routinely testing stool samples for *Cryptosporidium* spp. from the FoodNet Laboratory Survey (18). Uncertainty with this proportion (36%) was based on a 50% relative increase/decrease from 0.36 on an odds scale.	PERT	Low, modal, high values: 0.27, 0.36, 0.46

Test sensitivity	Average from published studies (18-22). Uncertainty with this proportion (87%) was based on a 50% relative increase/decrease from 0.87 on an odds scale.	PERT	Low, modal, high values: 0.81, 0.87, 0.91
Proportion hospitalized	Proportion of FoodNet cases of *Cryptosporidium* spp. infection who were hospitalized (2005-2008).	Empirical	By site and year (2005-2008) see Table 3.3 in this online Technical Appendix
Proportion who died	Proportion of FoodNet cases of *Cryptosporidium* spp. infection who died (2005-2008).	Empirical	By site and year (2005-2008) see Table 3.4 in this online Technical Appendix
Under-diagnosis (hospitalizations, deaths)	Number of hospitalizations and deaths doubled to account for under-diagnosis.	PERT	Low, modal, high values: 1, 2, 3
Proportion travel-related	Proportion of FoodNet cases of *Cryptosporidium* spp. infection who reported travel outside the United States within 15 days of illness onset (2005-2008). Uncertainty with this proportion (9%) was based on a 50% relative increase/decrease from 0.09 on an odds scale.	PERT	Low, modal, high values: 0.06, 0.09, 0.13
Proportion foodborne	Estimated based on data from a Canadian study (23). Uncertainty with this proportion (8%) was based on a 50% relative increase/decrease from 0.08 on an odds scale.	PERT	Low, modal, high values: 0.06, 0.08, 0.12

**Pathogen: *Cyclospora cayetanensis***			
**Model input**	**Data source(s)**	**Distribution**	**Parameters**
Reported illnesses	Incidence of *Cyclospora cayetanensis* infection reported to CDC's Foodborne Diseases Active Surveillance Network (FoodNet) by FoodNet site (n=10) and year (2005-2008) (10).	Empirical	By site and year (2005-2008) see Tables 3.1 and 3.2 in this online Technical Appendix
Population adjustment (year)	Incidence of *Cyclospora cayetanensis* in each FoodNet site by year applied to 2006 US Census population estimates (1).	Degenerate	Adjustment by year (2005-2008): 1.010, 1.000, 0.990, 0.981
Underreporting	No underreporting multiplier, we assumed all laboratory-confirmed *Cyclospora cayetanensis* illnesses were enumerated by FoodNet active surveillance.	-	-
Proportion severe	*Cyclospora cayetanensis* can cause severe diarrhea, though bloody diarrhea is rare. Proportion severe assumed to be 65% severe.	PERT	Low, modal, high values: 0.55, 0.65, 0.75

Medical care seeking (severe)	Proportion (and 95% confidence interval (CI)) of survey respondents with bloody diarrhea who sought medical care from FoodNet Population Surveys (2000-2001, 2002-2003, 2006-2007) as a proxy for severe illness (CDC, unpublished data).	PERT	Low, modal, high values: 0.19, 0.35, 0.51
Medical care seeking (mild)	Proportion (and 95% CI) of survey respondents with a non-bloody diarrhea who sought medical care from FoodNet Population Surveys (2000-2001, 2002-2003, 2006-2007) (CDC, unpublished data).	PERT	Low, modal, high values: 0.15, 0.18, 0.20
Specimen submission (severe)	Proportion (and 95% CI) of survey respondents who submitted a stool specimen among persons with bloody diarrhea who sought medical care from FoodNet Population Surveys (2000-2001, 2002-2003, 2006-2007) as a proxy for severe illness (CDC, unpublished data).	PERT	Low, modal, high values: 0.11, 0.36, 0.62
Specimen submission (mild)	Proportion (and 95% CI) of survey respondents who submitted a stool specimen among persons with a non-bloody diarrhea who sought medical care from FoodNet Population Surveys (2000-2001, 2002-2003, 2006-2007) (CDC, unpublished data).	PERT	Low, modal, high values: 0.12, 0.19, 0.25
Laboratory testing	Published studies (18, 24-28).	PERT	Low, modal, high values: 0.18, 0.25, 0.33
Test sensitivity	Published studies (18, 24-28).	PERT	Low, modal, high values: 0.73, 0.80, 0.86
Proportion hospitalized	Proportion of FoodNet cases of *Cyclospora cayetanensis* infection hospitalized (2005-2008).	Empirical	By site and year (2005-2008) see Table 3.3 in this online Technical Appendix
Proportion who died	Proportion of FoodNet cases of *Cyclospora cayetanensis* infection who died (2005-2008).	Empirical	By site and year (2005-2008) see Table 3.4 in this online Technical Appendix
Under-diagnosis (hospitalizations, deaths)	Number of hospitalizations and deaths doubled to account for under-diagnosis.	PERT	Low, modal, high values: 1, 2, 3
Proportion travel-related	Proportion of FoodNet cases of *Cyclospora cayetanensis* infection who reported travel outside the United States within 15 days of illness onset (2005-2008). Uncertainty with this proportion (42%) based on 50% relative increase/decrease from 0.42 on odds scale.	PERT	Low, modal, high values ; 0.32, 0.42, 0.52
Proportion foodborne	Based on outbreaks reported to CDC (29, 30)	PERT	Low, modal, high values: 0.98, 0.99, 1.00

**Pathogen: *Escherichia coli*, enterotoxigenic (ETEC)**			
**Model input**	**Data source(s)**	**Distribution**	**Parameters**
Reported illnesses	Number of ETEC outbreak-associated illnesses reported to CDC's Foodborne Disease Outbreak Surveillance System (2000-2007) (5).	Empirical	By year (2000-2007): 100, 42, 49, 55, 62, 39, 0, 66
Population adjustment (year)	Population ratios applied to each year from 2000-2007 based on US Census population estimates (1).	Degenerate	Ratios by year (2000-2007): 1.058, 1.047, 1.038, 1.029, 1.019, 1.010, 1.000, 0.990
Underreporting	Outbreak surveillance multiplier used to adjust for underreporting (see online Technical Appendix 4).	PERT	Low, modal, high, [precision] values: 5,16, 237, [20]
Proportion severe	Non-typhoidal *Salmonella* under-diagnosis multiplier used because of a lack of data on under-diagnosis factors. See Table 3.5 in this online Technical Appendix.		
Medical care seeking (severe)			
Medical care seeking (mild)			
Specimen submission (severe)			
Specimen submission (mild)			
Laboratory testing			
Test sensitivity			
Proportion hospitalized	Proportion of cases hospitalized in ETEC outbreaks reported to the Foodborne Disease Outbreak Surveillance System (2000-2007).	Empirical	By year (2000-2007): 0.000, 0.000, 0.000, 0.036, 0.016, 0.000, 0.000, 0.015
Proportion who died	Proportion of cases who died in foodborne ETEC outbreaks reported to the Foodborne Disease Outbreak Surveillance System (2000-2007).	Empirical	By year (2000-2007): 0.000, 0.000, 0.000, 0.000, 0.000, 0.000, 0.000, 0.000
Under-diagnosis (hospitalizations, deaths)	Number of hospitalizations and deaths doubled to account for under-diagnosis.	PERT	Low, modal, high values: 1, 2, 3

Proportion travel-related	55% based on surveillance data from MN FoodNet site (Minnesota Department of Health, unpublished data). Uncertainty with this proportion was based on a 50% relative increase/decrease from 0.55 on an odds scale.	PERT	Low, modal, high values: 0.45, 0.55, 0.65
Proportion foodborne	Estimates based on outbreak-associated illnesses from foodborne outbreaks reported to the Foodborne Disease Outbreak Surveillance System, therefore, estimated illnesses assumed to be 100% foodborne.	PERT	Low, modal, high values: 0.999, 1.000, 1.000

**Pathogen: *Escherichia coli*, Shiga toxin–producing (STEC) O157**			
**Model input**	**Data source(s)**	**Distribution**	**Paramters**
Reported illnesses	Incidence of STEC O157 infection reported to CDC's Foodborne Diseases Active Surveillance Network (FoodNet) by FoodNet site (n=10) and year (2005-2008) (10).	Empirical	By site and year (2005-2008) see Tables 3.1 and 3.2 in this online Technical Appendix
Population adjustment (year)	Incidence of STEC O157 in each FoodNet site by year applied to 2006 US Census population estimates (1).	Degenerate	Adjustment by year (2005-2008): 1.010, 1.000, 0.990, 0.981
Underreporting	No underreporting multiplier; we assumed that all laboratory-confirmed STEC O157 illnesses were enumerated by FoodNet active surveillance.	-	-
Percent severe	Proportion of cases by site reporting bloody diarrhea from FoodNet case-control study of sporadic laboratory-confirmed STEC O157 infections (31). We used uniform minimum variance unbiased (UMVU) estimators for lower and upper endpoints.	PERT	Low, modal, high values: 0.85, 0.90, 1.00
Medical care seeking (severe)	Proportion (and 95% confidence interval (CI)) of survey respondents with bloody diarrhea who sought medical care from FoodNet Population Surveys (2000-2001, 2002-2003, 2006-2007) (CDC, unpublished data).	PERT	Low, modal, high values: 0.19, 0.35, 0.51

Medical care seeking (mild)	Proportion (and 95% CI) of survey respondents with a non-bloody diarrhea who sought medical care from FoodNet Population Surveys (2000-2001, 2002-2003, 2006-2007) (CDC, unpublished data)	PERT	Low, modal, high values: 0.15, 0.18, 0.20
Specimen submission (severe)	Proportion (and 95% CI) of survey respondents who submitted a stool specimen among persons with bloody diarrhea who sought medical care from FoodNet Population Surveys (2000-2001, 2002-2003, 2006-2007) (CDC, unpublished data).	PERT	Low, modal, high values: 0.11, 0.36, 0.62
Specimen submission (mild)	Proportion (and 95% CI) of survey respondents who submitted a stool specimen among persons with a non-bloody diarrhea who sought medical care from FoodNet Population Surveys (2000-2001, 2002-2003, 2006-7) (CDC, unpublished data).	PERT	Low, modal, high values: 0.12, 0.19, 0.25
Laboratory testing	Proportion of clinical laboratories routinely testing stool samples for STEC O157 from the FoodNet Laboratory Survey (12).	PERT	Low, modal, high values: 0.48, 0.58, 0.67
Test sensitivity	We used a laboratory test sensitivity rate of 70% based on studies of *Salmonella* (13, 14). We assumed a lower bound of 60% and an upper bound of 90%.	PERT	Low, modal, high values: 0.60, 0.70, 0.90
Proportion hospitalized	Proportion of FoodNet cases of STEC O157 infection who were hospitalized (2005-2008).	Empirical	By site and year (2005-2008) see Table 3.3 in this online Technical Appendix
Proportion who died	Proportion of FoodNet cases of STEC O157 infection who died (2005-2008)	Empirical	By site and year (2005-2008) see Table 3.4 in this online Technical Appendix
Under-diagnosis (hospitalizations, deaths)	Number of hospitalizations and deaths doubled to account for under-diagnosis.	PERT	Low, modal, high values: 1, 2, 3
Proportion travel-related	Proportion of FoodNet cases of STEC O157 infection who reported travel outside the United States within 7 days of illness onset (2005-2008). Uncertainty with this proportion (3.5%) was based on a 50% relative increase/decrease from 0.035 on an odds scale.	PERT	Low, modal, high values: 0.02, 0.035, 0.05

Proportion foodborne	Proportion of STEC O157 outbreak-associated illnesses due to foodborne transmission from outbreaks reported to CDC (32). Uncertainty with this proportion (68%) was based on a 50% relative increase/decrease from 0.68 on an odds scale.	PERT	Low, modal, high values: 0.59, 0.68, 0.76

**Pathogen: *Escherichia coli*, Shiga-toxin-producing (STEC), non-O157**			
**Model input**	**Data source(s)**	**Distribution**	**Parameters**
Reported illnesses	Incidence of non-O157 STEC infection reported to CDC's Foodborne Diseases Active Surveillance Network (FoodNet) by FoodNet site (n=10) and year (2005-2008) (10).	Empirical	By site and year (2005-2008) see Tables 3.1 and 3.2 in this online Technical Appendix
Population adjustment (year)	Incidence of non-O157 STEC in each FoodNet site by year applied to 2006 US Census population estimates (1).	Degenerate	Adjustment by year (2005-2008): 1.010, 1.000, 0.990, 0.981
Underreporting	No underreporting multiplier; we assumed that all laboratory-confirmed non-O157 STEC illnesses were enumerated by FoodNet active surveillance.	-	-
Percent severe	Proportion of non-O157 STEC cases of infection with bloody diarrhea from study published study in Minnesota FoodNet site (33). Uncertainty with this proportion (54%) was based on a 50% relative increase/decrease from 0.54 on an odds scale.	PERT	Low, modal, high values: 0.44, 0.54, 0.64
Medical care seeking (severe)	Proportion (and 95% confidence interval (CI)) of survey respondents with bloody diarrhea who sought medical care from FoodNet Population Surveys (2000-2001, 2002-2003, 2006-2007) (CDC, unpublished data).	PERT	Low, modal, high values: 0.19, 0.35, 0.51
Medical care seeking (mild)	Proportion (and 95% CI) of survey respondents with a non-bloody diarrhea who sought medical care from FoodNet Population Surveys (2000-2001, 2002-2003, 2006-2007) (CDC, unpublished data).	PERT	Low, modal, high values: 0.15, 0.18, 0.20

Specimen submission (severe)	Proportion (and 95% CI) of survey respondents who submitted a stool specimen among persons with bloody diarrhea who sought medical care from FoodNet Population Surveys (2000-2001, 2002-2003, 2006-2007) (CDC, unpublished data).	PERT	Low, modal, high values: 0.11, 0.36, 0.62
Specimen submission (mild)	Proportion (and 95% CI) of survey respondents who submitted a stool specimen among persons with a non-bloody diarrhea who sought medical care from FoodNet Population Surveys (2000-2001, 2002-2003, 2006-7) (CDC, unpublished data).	PERT	Low, modal, high values: 0.12, 0.19, 0.25
Laboratory testing	Laboratory-confirmed non-O157 STEC illnesses assumed to be at least as common as STEC O157 (34, 35). Laboratory testing proportion estimated based on this assumption.	PERT	Low, modal, high values: 0.18, 0.25, 0.33
Test sensitivity	We used a laboratory test sensitivity rate of 70% based on studies of *Salmonella* (13, 14). We assumed a lower bound of 60% and an upper bound of 90%.	PERT	Low, modal, high values: 0.60, 0.70, 0.90
Proportion hospitalized	Proportion of FoodNet cases of non-O157 STEC infection hospitalized (2005-2008).	Empirical	By site and year (2005-2008): see Table 3.3 in this online Technical Appendix
Proportion who died	Proportion of FoodNet cases of non-O157 STEC infection who died (2005-2008).	Empirical	By site and year (2005-2008): see Table 3.4 in this online Technical Appendix
Under-diagnosis (hospitalizations, deaths)	Number of hospitalizations and deaths doubled to account for under-diagnosis.	PERT	Low, modal, high values: 1, 2, 3
Proportion travel-related	Proportion of FoodNet cases of non-O157 STEC infection who reported travel outside the United States within 7 days of illness onset (2005-2008). Uncertainty with this proportion (18%) was based on a 50% relative increase/decrease from 0.18 on an odds scale.	PERT	Low, modal, high values: 0.13, 0.18, 0.25
Proportion foodborne	Proportion of non-O157 STEC outbreak-associated illnesses due to foodborne transmission from outbreaks reported to CDC (1990-2008) (36). Uncertainty with this proportion (82%) was based on a 50% relative increase/decrease from 0.82 on an odds scale.	PERT	Low, modal, high values: 0.75, 0.82, 0.87

**Pathogen: *Escherichia coli*, diarrheagenic other than STEC and ETEC**			
**Model input**	**Data source(s)**	**Distribution**	**Parameters**
Reported illnesses	Assumed to be as common as enterotoxigenic *E. coli* because of a lack of available surveillance data or data on under-diagnosis factors.		
Population adjustment (year)			
Underreporting			
Percent severe			
Medical care seeking (severe)			
Medical care seeking (mild)			
Specimen submission (severe)			
Specimen submission (mild)			
Laboratory testing			
Laboratory test sensitivity			
Proportion hospitalized	Assumed to be the same as ETEC	Empirical	By year (2000-2007): 0.000, 0.000, 0.000, 0.036, 0.016, 0.000, 0.000, 0.015
Proportion who died	Assumed to be the same as ETEC	Empirical	By year (2000-2007): 0.000, 0.000, 0.000, 0.000, 0.000, 0.000, 0.000, 0.000
Under-diagnosis (hospitalizations, deaths)	Number of hospitalizations and deaths doubled to account for under-diagnosis.	PERT	Low, modal, high values: 1, 2, 3
Proportion travel-related	Assumed to be almost 100% domestically acquired.	PERT	Low, modal, high values: 0, 0, 0.02
Proportion foodborne	Very little data available, a few foodborne outbreaks have been reported. Assumed to be 30% foodborne (37). Uncertainty with this proportion was based on a 50% relative increase/decrease from 0.30 on an odds scale.	PERT	Low, modal, high values: 0.22, 0.30, 0.39

**Pathogen:** ***Giardia intestinalis***			
**Model input**	**Data source(s)**	**Distribution**	**Parameters**
Reported illnesses	Number of illnesses due to *Giardia intestinalis* reported to CDC's National Notifiable Diseases Surveillance System (NNDSS) (2002-2007) (6).	Empirical	By year (2002-2007): 21300, 19709, 20965, 19733, 18953, 19417
Population adjustment (year)	Population ratios applied to each year from 2002-2007 based on US Census population estimates (1).	Degenerate	Ratios by year (2002-2007): 1.038, 1.029, 1.019, 1.010, 1.000, 0.990
Underreporting	Passive surveillance multiplier used to adjust for underreporting (see online Technical Appendix 4).	PERT	Low, modal, high values: 1.0, 1.3, 1.6
Percent severe	Assumed to be mostly mild (17).	PERT	Low, modal, high values: 0.0, 0.0, 0.05
Medical care seeking (severe)	Proportion (and 95% confidence interval (CI)) of survey respondents with bloody diarrhea who sought medical care from FoodNet Population Surveys (2000-2001, 2002-2003, 2006-2007) (CDC, unpublished data).	PERT	Low, modal, high values: 0.19, 0.35, 0.51
Medical care seeking (mild)	Proportion (and 95% CI) of survey respondents with a non-bloody diarrhea who sought medical care from FoodNet Population Surveys (2000-2001, 2002-2003, 2006-2007) (CDC, unpublished data)	PERT	Low, modal, high values: 0.15, 0.18, 0.20
Specimen submission (severe)	Proportion (and 95% CI) of survey respondents who submitted a stool specimen among persons with bloody diarrhea who sought medical care from FoodNet Population Surveys (2000-2001, 2002-2003, 2006-2007) (CDC, unpublished data).	PERT	Low, modal, high values: 0.11, 0.36, 0.62
Specimen submission (mild)	Proportion (and 95% CI) of survey respondents who submitted a stool specimen among persons with a non-bloody diarrhea who sought medical care from FoodNet Population Surveys (2000-2001, 2002-2003, 2006-7) (CDC, unpublished data).	PERT	Low, modal, high values: 0.12, 0.19, 0.25
Laboratory testing	Based on consultations with clinical and billing code experts at CDC, in academia, and laboratories across the United States. Uncertainty with this proportion (80%) was based on a 50% relative increase/decrease from 0.80 on an odds scale.	PERT	Low, modal, high values: 0.73, 0.80, 0.86

Test sensitivity	Average from 10 published studies (19, 21, 38-45). We used uniform minimum variance unbiased (UMVU) estimators for lower and upper endpoints.	PERT	Low, modal, high values: 0.72, 0.83, 0.93
Proportion hospitalized	Proportion of cases hospitalized estimated using annual national estimates from the Nationwide Inpatient Sample (NIS) (2002-2006) using ICD-9-CM code 007.1 (Giardiasis) (46).	Empirical	By year (2002-2006): 0.085, 0.092, 0.083, 0.086, 0.095
Proportion who died	Proportion of cases who died estimated using annual national estimates from the NIS (2002-2006) using ICD-9-CM code 007.1 (Giardiasis).	Empirical	By year (2002-2006): 0.010, 0.0005, 0.0010, 0.0008, 0.0010
Under-diagnosis (hospitalizations, deaths)	Number of hospitalizations and deaths doubled to account for under-diagnosis.	PERT	Low, modal, high values: 1, 2, 3
Proportion travel-related	8% based on a published study (47). Uncertainty with this proportion was based on a 50% relative increase/decrease from 0.08 on an odds scale.	PERT	Low, modal, high values: 0.06, 0.08, 0.12
Proportion foodborne	7% based on outbreaks reported to CDC (CDC, unpublished data). Uncertainty with this proportion was based on a 50% relative increase/decrease from 0.07 on an odds scale.	PERT	Low, modal, high values: 0.05, 0.07, 0.10

**Pathogen: Hepatitis A**			
**Model input**	**Data source(s)**	**Distribution**	**Parameters**
Reported illnesses	Number of illnesses due to hepatitis A reported to CDC's National Notifiable Diseases Surveillance System (NNDSS) (2000-2007) (6, 48). Because of an apparent trend over time, the empirical distribution was based on the predicted count for 2006 plus empirical residuals derived from a linear regression of the number of illnesses on year (online Technical Appendix 2, www.cdc.gov/EID/content/17/1/7-Techapp2.pdf).	Empirical	By year (2000-2007): 13397, 10616, 8795, 7653, 5683, 4488, 3579, 2979

Population adjustment (year)	Population ratios applied to each year from 2000-2007 based on US Census population estimates (1).	Degenerate	Ratios by year (2000-2007): 1.058, 1.047, 1.038, 1.029, 1.019, 1.010, 1.000, 0.990
Underreporting	Passive surveillance multiplier used to adjust for underreporting (see online Technical Appendix 4)	PERT	Low, modal, high values: 0.9, 1.1, 1.3
Proportion severe	Approximately 70% of infected persons have jaundice. Therefore, assumed to be 70% severe (48).	PERT	0.61, 0.70, 0.78
Medical care seeking (severe)	Assumed to have a high rate of medical care seeking.	PERT	Low, modal, high values: 0.80, 0.90, 1.00
Medical care seeking (mild)	Proportion (and 95% CI) of survey respondents with a non-bloody diarrhea who sought medical care from FoodNet Population Surveys (2000-2001, 2002-2003, 2006-2007) used as proxy for mild illness (CDC, unpublished data).	PERT	Low, modal, high values: 0.15, 0.18, 0.20
Specimen submission (severe)	Assumed to be 100%.	PERT	Low, modal, high values: 0.99, 1.00, 1.00
Specimen submission (mild)	Proportion (and 95% CI) of survey respondents who submitted a stool specimen among persons with a non-bloody diarrhea who sought medical care from FoodNet Population Surveys (2000-2001, 2002-2003, 2006-2007) used as a proxy for mild illness (CDC, unpublished data).	PERT	Low, modal, high values: 0.12, 0.19, 0.25
Laboratory testing	We assumed that most persons with hepatitis A who submitted a specimen for testing would be tested for hepatitis A.	PERT	Low, modal, high values: 0.94, 0.97, 1
Laboratory test sensitivity	Assumed to be almost 100%.	PERT	Low, modal, high values: 0.94, 0.97, 1
Proportion hospitalized	NNDSS data on proportion of cases of hepatitis A infection hospitalized (2001-2007). Data from 2001 were used because of hospitalizations were more carefully evaluated since 2001.	Empirical	By year (2001-2007): 0.288, 0.261, 0.318, 0.328, 0.330, 0.330, 0.350
Proportion who died	Estimated using multiple cause-of-death mortality data from the national vital statistics system (49, 50) and doubled to adjust for under-diagnosis.	Empirical	By year (2004-2007): 0.023, 0.022, 0.022, 0.029
Under-diagnosis (hospitalizations)	Number of hospitalizations doubled to account for under-diagnosis.	PERT	Low, modal, high values: 1, 2, 3

Proportion travel-related	41% based on enhanced surveillance in 6 US states (2005-2007) (51). Uncertainty with this proportion was based on a 50% relative increase/decrease from 0.41 on an odds scale.	PERT	Low, modal, high values: 0.32, 0.41, 0.51
Proportion foodborne	6% based on exposure data from NNDSS (2000-2007).	PERT	Low, modal, high values: 0.035, 0.063, 0.16

**Pathogen: *Listeria monocytogenes***			
**Model input**	**Data source(s)**	**Distribution**	**Distribution values**
Reported illnesses	Incidence of invasive *Listeria monocytogenes* infection reported to CDC's Foodborne Diseases Active Surveillance Network (FoodNet) by FoodNet site (n=10) and year (2005-2008) (10).	Empirical	By site and year (2005-2008) see Table 3.1 and 3.2 in this online Technical Appendix
Population adjustment (year)	Incidence of *Listeria monocytogenes* infection in each FoodNet site by year applied to 2006 US Census population estimates (1).	Degenerate	Adjustment by year (2005-2008): 1.010, 1.000, 0.990, 0.981
Underreporting	No underreporting multiplier, we assumed that all laboratory-confirmed *Listeria monocytogenes* illnesses were enumerated by FoodNet active surveillance.	-	-
Percent severe	Almost all cases of infection assumed to be severe. Only invasive infections included here.	PERT	Low, modal, high values: 0.95, 1.00, 1.00
Medical care seeking (severe)	Assumed to have a high rate of medical care seeking.	PERT	Low, modal, high values: 0.80, 0.90, 1.00
Medical care seeking (mild)	Assumed to have a high rate of medical care seeking.	PERT	Low, modal, high values: 0.80, 0.90, 1.00
Specimen submission (severe)	Assumed to have a high rate of specimen submission.	PERT	Low, modal, high values: 0.70, 0.80, 0.90
Specimen submission (mild)	Assumed to have a high rate of specimen submission.	PERT	Low, modal, high values: 0.70, 0.80, 0.90
Laboratory testing	We assumed that most persons with listeriosis who submitted a specimen for testing would be tested for listeriosis.	PERT	Low, modal, high values: 0.94, 0.97, 1.00
Laboratory test sensitivity	71% based on published study of blood culture sensitivity (52).	PERT	Low, modal, high values: 0.55, 0.71, 0.83

Proportion hospitalized	Proportion of FoodNet cases of *Listeria monocytogenes* infection who were hospitalized (2005-2008).	Empirical	By site and year (2005-2008) see Table 3.3 in this online Technical Appendix
Proportion who died	Proportion of FoodNet cases of *Listeria monocytogenes* infection who died (2005-2008).	Empirical	By site and year (2005-2008) see Table 3.4 in this online Technical Appendix
Under-diagnosis (hospitalizations, deaths)	Number of hospitalizations and deaths doubled to account for under-diagnosis.	PERT	Low, modal, high values: 1, 2, 3
Proportion travel-related	Proportion of FoodNet cases of *Listeria monocytogenes* infection who reported travel outside the United States within 30 days of illness onset (2005-2008). Uncertainty with this proportion (3%) was based on a 50% relative increase/decrease from 0.03 on an odds scale.	PERT	Low, modal, high values: 0.02, 0.03, 0.05
Proportion foodborne	Assumed to be almost 100% foodborne (53-57).	PERT	Low, modal, high values: 0.999, 1.000, 1.000

**Pathogen: *Mycobacterium bovis***			
**Model input**	**Data source(s)**	**Distribution**	**Parameters**
Reported TB illnesses	Number of tuberculosis (58) illnesses reported to CDC's National Tuberculosis Surveillance System (NTSS) (2004-2007) (59).	Empirical	By year (2004-2007): 14500, 14067, 13727, 13288
M. bovis fraction	Fraction of TB attributed to *Mycobacterium bovis* (60). Uncertainty with this proportion was based on a 50% relative increase/decrease from 0.014 on an odds scale.	PERT	Low, modal, high values: 0.011, 0.014, 0.017
Population adjustment (year)	Population ratios applied to each year from 2004-2007 based on US Census population estimates (1).	Degenerate	Ratio by year (2004-2007): 1.019, 1.010, 1.000, 0.990
Underreporting	No underreporting multiplier. We assumed that all cases of *Mycobacterium bovis* infection were reported to NTSS.	-	-
Proportion severe	Almost all cases assumed to be severe.	PERT	Low, modal, high values: 95, 1.00, 1.00

Medical care seeking (severe)	Assumed to be 100%	PERT	Low, modal, high values: 0.999, 1.00, 1.00
Medical care seeking (mild)	Assumed to be 100%	PERT	Low, modal, high values: 0.999, 1.00, 1.00
Specimen submission (severe)	Assumed to be 100%	PERT	Low, modal, high values: 0.999, 1.00, 1.00
Specimen submission (mild)	Assumed to be 100%	PERT	Low, modal, high values: 0.999, 1.00, 1.00
Laboratory testing	Assumed to be almost 100%	PERT	Low, modal, high values: 0.94, 0.97, 1.00
Test sensitivity	Assumed to be almost 100%	PERT	Low, modal, high values: 0.94, 0.97, 1.00
Proportion hospitalized	Limited data available on *Mycobacterium bovis* . Assumed to be 55% based on a study of hospitalizations among persons with TB (61). Uncertainty with this proportion was based on a 50% relative increase/decrease from 0.55 on an odds scale.	Empirical	Values: 0.45, 0.55, 0.65
Proportion who died	Limited data available on *Mycobacterium bovis*. Assumed to be equal to the proportion of TB cases who died in NTSS (2004-2007).	Empirical	By year (2004-2007): 0.050, 0.048, 0.046, 0.044
Proportion travel-related	70% of cases assumed to be travel-related. Uncertainty with this proportion (70%) was based on a 50% relative increase/decrease from 0.70 on an odds scale.	PERT	Low, modal, high values: 0.61, 0.70, 0.78
Proportion foodborne	Assumed to be 95% based on published study (62) Uncertainty with this proportion was based on a 50% relative increase/decrease from 0.95 on an odds scale.	PERT	Low, modal, high values: 0.93, 0.95, 0.97

Pathogen: Norovirus			
**Model input**	**Data source(s)**	**Distribution**	**Parameters**
Population at risk	Estimated using 2006 US Census population estimate.	Constant	299,000,000
Norovirus fraction	The proportion of all acute gastroenteritis illnesses, hospitalizations, and deaths was estimated from published studies of the proportion of acute gastroenteritis illnesses due to norovirus in the Netherlands (58), England and Wales (63, 64), and Australia (65). The proportions from these studies, .06, .11, .11, .20, were used to define low (0.06), modal (0.11) and high (0.20) values. The decision to apply this distribution to estimates of the number of acute gastroenteritis hospitalizations and deaths was supported by published studies of hospitalizations (66, 67).	PERT	Low, modal, high values: 0.06, 0.11, 0.2
Norovirus illnesses	Norovirus fraction (above) applied to estimated number of acute gastroenteritis illness (below)		
Acute gastroenteritis illnesses	Estimated rate per person per year by site using combined data from FoodNet Population Surveys in 2000–2001 (0.49 per person per year), 2002–2003 (0.54 per person per year), and 2006–2007 (0.73 per person per year) (CDC, unpublished data). Uncertainty from the site-specific survey estimates was added by assuming that site estimates were normally distributed with standard deviations equal to survey standard errors.	Mixture of Normals	By FoodNet site: 0.61, 0.63, 0.51, 0.68, 0.51, 0.56, 0.63, 0.63, 0.56, 0.65
Norovirus hospitalizations	Norovirus fraction (above) applied to estimated number of acute gastroenteritis hospitalizations (below).		
Proportion hospitalized	Estimated rate per 100,000 using annual national estimates from the 2000-2006 National Hospital Discharge System (186.3, 205.0, 211.1, 203.8, 203.0, 204.0, and 206.6 per 100,000) (68), the 2000-2006 Nationwide Inpatient Sample (177.1, 181.4, 183.4, 189.3, 183.9, 190.6, and 203.9 per 100,000) (46), and combined data from the 2000-2006 National Ambulatory Medical Care and National Hospital Ambulatory Medical Care Surveys (92.1, 94.7, 138.8, 110.1, 111.7, 90.4, and 126.3 per 100,000) (69). Low, modal, and high values were determined using the lowest (90), mean (166), and highest (211) annual rate per 100,000.	PERT	Low, modal, high values: 0.0015, 0.0028, 0.0035
Norovirus deaths	Norovirus fraction (above) applied to estimated number of acute gastroenteritis deaths (below).		

Proportion who died	Estimated annual rate per 100,000 persons using data from 2000–2006 multiple cause-of-death data from the national vital statistics system (49, 50) (2.4, 1.2, 1.3, 1.3, 1.3, 1.6, and 1.7 per 100,000) (50). Low, modal, and high values were determined using the lowest (1.2), mean (1.5), and highest (2.4) annual rate per 100,000.	PERT	Low, modal, high values: 0.00002, 0.000026, 0.00004
Proportion travel-related	Assumed to be low.	PERT	Low, modal, high values: 0.00, 0.00, 0.02
Proportion foodborne	Based on 179 norovirus outbreaks examined by CDC from 2000-2005. Of 13,944 persons ill, 3,628 (26%) were in foodborne outbreaks (CDC, unpublished data).	PERT	Low, modal, high values: 0.19, 0.26, 0.35

**Pathogen: Rotavirus**			
**Model input**	**Data source(s)**	**Distribution**	**Parameters**
Person-time at risk	The person-time at risk for 2006 was estimated as the 0-4 year population (20,417,636) divided by 5 and rounded (1).	Constant	4,123,000
Proportion ill	75% of children assumed to experience an episode of clinical illness due to rotavirus by 5 years of age based on published studies (2). Uncertainty with this proportion was based on a 50% relative increase/decrease from 0.75 on an odds scale.	Uniform	Low, high values: 0.55, 0.95
Proportion hospitalized	Based on published study (2).	PERT	Low, modal, high values: 0.012, 0.017, 0.023
Proportion who died	Very low: 20 to 40 deaths per year (2).	Uniform	Low, high values: 0.0000054, 0.00001
Proportion travel-related	Assumed to be 100% domestically acquired.	PERT	Low, modal, high values: 0.000, 0.000, 0.001
Proportion foodborne	Very few foodborne outbreaks reported (0.5% of illnesses).	PERT	Low, modal, high values: 0.000, 0.005, 0.010

**Pathogen: *Salmonella enterica,* non-typhoidal serotypes**			
**Model input**	**Data source(s)**	**Distribution**	**Distribution values**
Reported illnesses	Incidence of *Salmonella enterica* infections excluding serotype Typhi reported to CDC's Foodborne Diseases Active Surveillance Network (FoodNet) by FoodNet site (n=10) and year (2005-2008) (10).	Empirical	By site and year (2005-2008): See Table 3.1 and 3.2 in this online Technical Appendix
Population adjustment (year)	Incidence of non-typhoidal *Salmonella* in each FoodNet site by year applied to 2006 US Census population estimates (1).	Degenerate	Adjustment by year (2005-2008): 1.010, 1.000, 0.990, 0.981
Underreporting	No underreporting multiplier, we assumed that all laboratory-confirmed non-typhoidal *Salmonella* illnesses were enumerated by FoodNet active surveillance.	-	-
Percent severe	Proportion of cases reporting bloody diarrhea in FoodNet case-control studies of sporadic laboratory-confirmed *Salmonella* infections (70-73). We used uniform minimum variance unbiased (UMVU) estimators for lower and upper endpoints.	PERT	Low, modal, high values: 0.35, 0.45, 0.71
Medical care seeking (severe)	Proportion (and 95% confidence interval (CI)) of survey respondents with bloody diarrhea who sought medical care from FoodNet Population Surveys (2000-2001, 2002-2003, 2006-2007) (CDC, unpublished data).	PERT	Low, modal, high values: 0.19, 0.35, 0.51
Medical care seeking (mild)	Proportion (and 95% CI) of survey respondents with a non-bloody diarrhea who sought medical care from FoodNet Population Surveys (2000-2001, 2002-2003, 2006-2007) (CDC, unpublished data)	PERT	Low, modal, high values: 0.15, 0.18, 0.20
Specimen submission (severe)	Proportion (and 95% CI) of survey respondents who submitted a stool specimen among persons with bloody diarrhea who sought medical care from FoodNet Population Surveys (2000-2001, 2002-2003, 2006-2007) (CDC, unpublished data).	PERT	Low, modal, high values: 0.11, 0.36, 0.62
Specimen submission (mild)	Proportion (and 95% CI) of survey respondents who submitted a stool specimen among persons with a non-bloody diarrhea who sought medical care from FoodNet Population Surveys (2000-2001, 2002-2003, 2006-2007) (CDC, unpublished data).	PERT	Low, modal, high values: 0.12, 0.19, 0.25

Laboratory testing	100% of clinical laboratories reported routinely testing stool samples for *Salmonella* in the FoodNet Laboratory Survey (12).We assumed a slightly lower rate of 97%; uncertainty with this proportion was based on a 50% relative increase/decrease from 0.97 on an odds scale.	PERT	Low, modal, high values: 0.94, 0.97, 1.00
Laboratory test sensitivity	We assumed a laboratory test sensitivity rate of 70% based on studies of *Salmonella*. (13, 14). We assumed a lower bound of 60% and an upper bound of 90%.	PERT	Low, modal, high values: 0.60, 0.70, 0.90
Proportion hospitalized	Proportion of FoodNet cases of non-typhoidal *Salmonella* infection who were hospitalized (2005-2008).	Empirical	By site and year (2005-2008): See Table 3.3 in this online Technical Appendix
Proportion who died	Proportion of FoodNet cases of non-typhoidal *Salmonella* infection who died (2005-2008).	Empirical	By site and year (2005-2008): See Table 3.4 in this online Technical Appendix
Under-diagnosis (hospitalizations, deaths)	Number of hospitalizations and deaths doubled to account for under-diagnosis.	PERT	Low, modal, high values: 1, 2, 3
Proportion travel-related	Proportion of FoodNet cases of non-typhoidal *Salmonella* infection who reported travel outside the United States within 7 days of illness onset (2005-2008). Uncertainty with this proportion (11%) was based on a 50% relative increase/decrease from 0.11 on an odds scale.	PERT	Low, modal, high values: 0.07, 0.11, 0.15
Proportion foodborne	94% based on FoodNet case-control study of sporadic illness (72) and on outbreaks reported to the CDC from 1996-2006 (CDC, unpublished data) (see online Technical Appendix 1). Uncertainty with this proportion was based on a 50% relative increase/decrease from 0.94 on an odds scale.	PERT	Low, modal, high values: 0.91, 0.94, 0.96

Pathogen: *Salmonella* enterica, serotype Typhi			
**Model input**	**Data source(s)**	**Distribution**	**Distribution values**
Reported illnesses	Incidence of *Salmonella* serotype Typhi infection reported to CDC's Foodborne Diseases Active Surveillance Network (FoodNet) by FoodNet site (n=10) and year (2005-2008) (10).	Empirical	By site and year (2005-2008): See Table 3.1 and 3.2 in this online Technical Appendix
Population adjustment (year)	Incidence of serotype Typhi in each FoodNet site by year applied to 2006 US Census population estimates (1).	Degenerate	Adjustment by year (2005-2008): 1.010, 1.000, 0.990, 0.981
Underreporting multiplier	No underreporting multiplier; we assumed that all laboratory-confirmed serotype *Typhi* illnesses were enumerated by FoodNet active surveillance.	-	-
Percent severe	Almost all cases of serotype Typhi infections assumed to be severe.	PERT	Low, modal, high values: 0.95, 1.00, 1.00
Medical care seeking (severe)	Proportion (and 95% confidence interval (CI)) of survey respondents with bloody diarrhea who sought medical care from FoodNet Population Surveys (2000-2001, 2002-2003, 2006-2007) used as a proxy for severe illness (CDC, unpublished data).	PERT	Low, modal, high values: 0.19, 0.35, 0.51
Medical care seeking (mild)	Proportion (and 95% CI) of survey respondents with a non-bloody diarrhea who sought medical care from FoodNet Population Surveys (2000-2001, 2002-2003, 2006-2007) used as proxy for mild illness (CDC, unpublished data).	PERT	Low, modal, high values: 0.15, 0.18, 0.20
Specimen submission (severe)	Proportion (and 95% CI) of survey respondents who submitted a stool specimen among persons with bloody diarrhea who sought medical care from FoodNet Population Surveys (2000-2001, 2002-2003, 2006-2007) used as proxy for severe illness (CDC, unpublished data).	PERT	Low, modal, high values: 0.11, 0.36, 0.62
Specimen submission (mild)	Proportion (and 95% CI) of survey respondents who submitted a stool specimen among persons with a non-bloody diarrhea who sought medical care from FoodNet Population Surveys (2000-2001, 2002-2003, 2006-2007) used as a proxy for mild illness (CDC, unpublished data).	PERT	Low, modal, high values: 0.12, 0.19, 0.25
Laboratory testing	We assumed that almost all persons with serotype Typhi would be tested for serotype Typhi.	PERT	Low, modal, high values: 0.94, 0.97, 1.00
Test sensitivity	60-80% based on published review (74).	PERT	Low, modal, high values: 0.60, 0.70, 0.80

Proportion hospitalized	Proportion of FoodNet cases of serotype Typhi infection who were hospitalized (2005-2008).	Empirical	By site and year (2005-2008): See Table 3.3 in this online Technical Appendix
Proportion who died	Proportion of Foodnet cases of serotype Typhi infection who died (2005-2008).	Empirical	By site and year (2005-2008): See Table 3.4 in this online Technical Appendix
Under-diagnosis (hospitalizations, deaths)	Number of hospitalizations and deaths double to account for under-diagnosis.	PERT	Low, modal, high values: 1, 2, 3
Proportion travel-related	Proportion of FoodNet cases of serotype Typhi infection who reported travel outside the United States within 30 days of illness onset (2005-2008). Uncertainty with this proportion (67%) was based on a 50% relative increase/decrease from 0.67 on an odds scale.	PERT	Low, modal, high values: 0.58, 0.67, 0.76
Proportion foodborne	100% of domestically acquired outbreaks reported to the CDC between 1980 and 1999 were foodborne (100% of 13 [out of 17] outbreaks with a known route of transmission) (75). Lower bound set at 76% (13 of 17 outbreaks).	PERT	Low, modal, high values: 0.76, 1, 1

**Pathogen: Sapovirus**			
**Model input**	**Data source(s)**	**Distribution**	**Parameters**
Person-time at risk	The person-time at risk for 2006 was estimated as the 0-4 year population (20,417,636) divided by 5 and rounded (1).	Constant	4,123,000
Proportion ill	75% of children assumed to experience an episode of clinical illness due to sapovirus by five years of age based on studies of rotavirus (2). Uncertainty with this proportion was based on a 50% relative increase/decrease from 0.75 on an odds scale.	Uniform	Low, high values: 0.55, 0.95
Proportion hospitalized	Hospitalization rate derived as 25% of rotavirus.	PERT	Low, modal, high values: 0.003, 0.004, 0.006
Proportion who died	Assumed to be very low: 0–10 deaths per year	Uniform	Low, high values: 0.000, 0.0000024
Proportion travel-related	Assumed to be 100% domestically acquired.	PERT	Low, modal, high values: 0.000, 0.000, 0.001

Proportion foodborne	Very few foodborne outbreaks reported (<1% of all sapovirus illnesses)	PERT	Low, modal, high values: 0.000, 0.005, 0.010

**Pathogen: *Shigella* spp.**			
**Model input**	**Data source(s)**	**Distribution**	**Distribution values**
Reported illnesses	Incidence of *Shigella* infection reported to CDC's Foodborne Diseases Active Surveillance Network (FoodNet) by FoodNet site (n=10) and year (2005-2008) (10)	Empirical	By site and year (2005-2008): See Table 3.1 and 3.2 in this online Technical Appendix
Population adjustment (year)	Incidence of *Shigella* spp. in each FoodNet site by year applied to 2006 US Census population estimates (1).	Degenerate	Adjustment by year (2005-2008): 1.010, 1.000, 0.990, 0.981
Underreporting	No underreporting multiplier; we assumed that all laboratory-confirmed *Shigella* spp. illnesses were enumerated by FoodNet active surveillance.	-	-
Percent severe	Percent of laboratory-confirmed cases of *Shigella* spp. infection with bloody diarrhea reported to FoodNet surveillance in Minnesota and New York (Minnesota Department of Health and New York Department of Health, unpublished data). We used uniform minimum variance unbiased (UMVU) estimators for lower and upper endpoints.	PERT	Low, modal, high values: 0.17, 0.35, 0.53
Medical care seeking (severe)	Proportion (and 95% confidence interval (CI)) of survey respondents with bloody diarrhea who sought medical care from FoodNet Population Surveys (2000-2001, 2002-2003, 2006-2007) (CDC, unpublished data).	PERT	Low, modal, high values: 0.19, 0.35, 0.51
Medical care seeking (mild)	Proportion (and 95% CI) of survey respondents with a non-bloody diarrhea who sought medical care from FoodNet Population Surveys (2000-2001, 2002-2003, 2006-2007) (CDC, unpublished data)	PERT	Low, modal, high values: 0.15, 0.18, 0.20

Specimen submission (severe)	Proportion (and 95% CI) of survey respondents who submitted a stool specimen among persons with bloody diarrhea who sought medical care from FoodNet Population Surveys (2000-2001, 2002-2003, 2006-2007) (CDC, unpublished data).	PERT	Low, modal, high values: 0.11 0.36, 0.62
Specimen submission (mild)	Proportion (and 95% CI) of survey respondents who submitted a stool specimen among persons with a non-bloody diarrhea who sought medical care from FoodNet Population Surveys (2000-2001, 2002-2003, 2006-2007) (CDC, unpublished data).	PERT	Low, modal, high values: 0.12, 0.19, 0.25
Laboratory testing	Proportion of clinical laboratories routinely testing stool samples for *Shigella spp.* from the FoodNet Laboratory Survey (12). We assumed a slightly lower rate of 97%, uncertainty with this proportion was based on a 50% relative increase/decrease from 0.97 on an odds scale.	PERT	Low, modal, high values: 0.94, 0.97, 1.00
Test sensitivity	We used a laboratory test sensitivity rate of 70% based on studies of *Salmonella* (13, 14). We assumed a lower bound of 60% and an upper bound of 90%.	PERT	Low, modal, high values: 0.60, 0.70, 0.90
Proportion hospitalized	Proportion of FoodNet cases of *Shigella* spp. infection who were hospitalized (2005-2008).	Empirical	By site and year (2005-2008): See Table 3.3 in this online Technical Appendix
Proportion who died	Proportion of FoodNet cases of *Shigella* spp. infection who died (2005-2008).	Empirical	By site and year (2005-2008): See Table 3.4 in this online Technical Appendix
Under-diagnosis (hospitalizations, deaths)	Number of hospitalizations and deaths doubled to account for under-diagnosis.	PERT	Low, modal, high values: 1, 2, 3
Proportion travel-related	Proportion of FoodNet cases of *Shigella* spp. infection who reported travel outside the United States within 7 days of illness onset (2005-2008). Uncertainty with this proportion (15%) was based on a 50% relative increase/decrease from 0.15 on an odds scale.	PERT	Low, modal, high values: 0.10, 0.15, 0.21
Proportion foodborne	31% based on FoodNet enhanced surveillance (76) with this proportion was based on a 50% relative increase/decrease from 0.31 on an odds scale.	PERT	Low, modal, high values: 0.23, 0.31, 0.40

Pathogen: ***Staphylococcus aureus***			
**Model input**	**Data source(s)**	**Distribution**	**Distribution values**
Reported illnesses	Number of *Staphylococcus aureus* outbreak-associated illnesses reported to CDC's Foodborne Disease Outbreak Surveillance System (2000-2007) (5). Because of an apparent trend over time, the empirical distribution was based on the predicted count for 2006 plus empirical residuals derived from a linear regression of the number of illnesses on year (see online Technical Appendix 2).	Empirical	By year (2000-2007):<br>650, 679, 551, 393, 450, 376, 380, 245
Population adjustment (year)	Population ratios applied to each year from 1998-2006 based on US Census population estimates (1).	Degenerate	Ratios by year (2000-2007):<br>1.058, 1.047, 1.038, 1.029, 1.019, 1.010, 1.000, 0.990
Underreporting multiplier	Outbreak surveillance multiplier used to adjust for underreporting (see online Technical Appendix 4)	PERT	Low, modal, high, [precision] values:<br>5,16, 237, [20]
Proportion severe	Non-typhoidal *Salmonella* under-diagnosis multiplier used because of a lack of data on under-diagnosis factors. See Table 3.5 in this online Technical Appendix.		
Medical care seeking (severe)			
Medical care seeking (mild)			
Specimen submission (severe)			
Specimen submission (mild)			
Laboratory testing			
Test sensitivity			
Proportion hospitalized	Proportion of cases hospitalized in *Staphylococcus aureus* outbreaks from the Foodborne Disease Outbreak Surveillance System (2000-2007).	Empirical	By year (2000-2007):<br>0.087, 0.115, 0.080, 0.059, 0.044, 0.082, 0.021, 0.020
Proportion who died	Proportion of cases who died in *Staphylococcus aureus* outbreaks from the Foodborne Disease Outbreak Surveillance System (2000-2007).	Empirical	By year (2000-2007):<br>0.003, 0.000, 0.000, 0.000, 0.000, 0.000, 0.000, 0.000

Under-diagnosis (hospitalizations, deaths)	Number of hospitalizations and deaths doubled to account for under-diagnosis.	PERT	Low, modal, high values: 1, 2, 3
Proportion travel-related	Because of the rapid onset and short duration of *Staphylococcus aureus* illnesses, we assumed that almost 100% of *Staphylococcus aureus* illnesses occurring in the United States would be domestically acquired.	PERT	Low, modal, high values: 0.00, 0.00, 0.02
Proportion foodborne	Estimates based on outbreak-associated illnesses from foodborne outbreaks reported to the Foodborne Disease Outbreak Surveillance System, therefore, assumed to be 100% foodborne.	PERT	Low, modal, high values: 0.999, 1.000, 1.000

**Pathogen: *Streptococcus* spp., Group A**			
**Model input**	**Data source(s)**	**Distribution**	**Distribution values**
Reported illnesses	Number of *Streptococcus* spp.,Group A outbreak-associated illnesses reported to CDC's Foodborne Disease Outbreak Surveillance System (1996-2007) (5).	Empirical	By year (1996-2007): 0, 122, 4, 0, 0, 0, 0, 0, 37, 0, 0, 0
Population adjustment (year)	Population ratios applied to each year from 1996-2007 based on US Census population estimates (1).	Degenerate	Ratios by year (1996-2007): 1.126, 1.115, 1.105, 1.095, 1.058, 1.047, 1.038, 1.029, 1.019, 1.010, 1.000, 0.990
Underreporting	Outbreak surveillance multiplier used to adjust for underreporting (see online Technical Appendix 4)	PERT	Low, modal, high, [precision] values: 5,16, 237, [20]

Proportion severe	Non-typhoidal *Salmonella* under-diagnosis multiplier used because of a lack of data on under-diagnosis factors. See Table 3.5 in this online Technical Appendix.		
Medical care seeking (severe)			
Medical care seeking (mild)			
Specimen submission (severe)			
Specimen submission (mild)			
Laboratory testing			
Test sensitivity			
Proportion hospitalized	Proportion of *Streptococcus* spp., Group A cases hospitalized from the Foodborne Disease Outbreak Surveillance System (1981-2007, 12 years when outbreaks occurred). Note, the outlier value of 4/4=100 hospitalization in 1998 was shrunk to 0.012, the next highest value in the ordered list of 12 rates.	Empirical	Outbreak years (1981-2007): 0.000, 0.000, 0.012, 0.000, 0.000, 0.000, 0.000, 0.000, 0.004, 0.000, 1.000, 0.000
Proportion who died	Proportion of *Streptococcus* spp., Group A cases who died from the Foodborne Disease Outbreak Surveillance System (1981-2007, 12 years when outbreaks occurred).	Empirical	Outbreak years (1981-2007): 0.000, 0.000, 0.000, 0.000, 0.000, 0.000, 0.000, 0.000, 0.000, 0.000, 0.000, 0.000
Under-diagnosis (hospitalizations, deaths)	Number of hospitalizations and deaths doubled to account for under-diagnosis.	PERT	Low, modal, high values: 1, 2, 3
Proportion travel-related	Because of the rapid onset and short duration of *Streptococcus* spp., Group A illnesses, we assumed that almost 100% of *Streptococcus* spp., Group A illnesses occurring in the United States are domestically acquired.	PERT	Low, modal, high values: 0.00, 0.00, 0.02
Proportion foodborne	Estimates based on outbreak-associated illnesses from foodborne outbreaks reported to CDC, therefore, assumed to be 100% foodborne.	PERT	Low, modal, high values: 0.999, 1.00, 1.00

Pathogen: ***Toxoplasma gondii***			
**Model input**	**Data source(s)**	**Distribution**	**Parameters**
Prevalence	Prevalence of *Toxoplasma gondii* infection estimated using nationally representative serologic data from the National Health and Nutrition Examination Survey (NHANES) (1999-2004). Specifically, the estimated prevalence for persons aged 40-49 years reported in Jones *et al.* (77) was assumed to be the cumulative result of 45 years of constant incidence. Upper and lower limits were based on the published 95% confidence interval.	Constant	Low, modal, high values: 0.137, 0.157, 0.177
Incidence	Prevalence for persons aged 40-49 years reported in Jones *et al.* (77) was converted to annual incidences using the following formula: 1-(1-Prev%/100)^(1/45). Incidence was applied to 2006 US Census population estimates (299 million persons). Upper and lower limits of the incidence distribution were obtained by direct conversion of the 95% confidence interval.	Degenerate	0.00327, 0.00379, 0.00432
Seroconverison rate	The symptomatic fraction was estimated to be 15% (78). Uncertainty with this proportion was based on a 50% relative increase/decrease from 0.15 on an odds scale.	PERT	Low, modal, high values: 0.11, 0.15, 0.21
Proportion hospitalized	Low, modal, and high values estimated from the annual national estimates of the number of toxoplasmosis hospitalizations from the 2000-2006 Nationwide Inpatient Sample (NIS) (46) using ICD-9-CM code 130 (Toxoplasmosis).	PERT	Low, modal, high values: 0.017, 0.026, 0.033
Proportion who died	Low, modal, and high values estimated from the annual national estimates of the number of toxoplasmosis inpatient deaths from the 2000-2006 NIS using ICD-9-CM code 130 (Toxoplasmosis).	PERT	Low, modal, high values 0.0014, 0.0019 , 0.0022
Under-diagnosis (hospitalizations, deaths)	Number of hospitalizations and deaths doubled to account for under-diagnosis.	PERT	Low, modal, high values: 1, 2, 3
Proportion travel-related	Assumed to be very low.	PERT	Low, modal, high values: 0, 0, 0.2
Proportion foodborne	50% based on published studies (79, 80). Uncertainty with this proportion was based on a 50% relative increase/decrease from 0.50 on an odds scale.	PERT	Low, modal, high values: 0.40, 0.50, .060

**Pathogen: *Trichinella* spp.**			
**Model input**	**Data source(s)**	**Distribution**	**Parameters**
Reported illnesses	Number of illnesses due to *Trichinella* spp. reported to CDC's National Notifiable Diseases Surveillance System (NNDSS) (2000-2007) (6, 81).	Empirical	By year (2000-2007): 16, 22, 14, 6, 5, 16, 15, 5
Population adjustment (year)	Population ratios applied to each year from 2000-2007 based on US Census population estimates (1).	Degenerate	Ratios by year (2000-2007): 1.058, 1.047, 1.038, 1.029, 1.019, 1.010, 1.000, 0.990
Underreporting	Passive surveillance multiplier used to adjust for underreporting (see online Technical Appendix 4).	PERT	Low, modal, high values: 1.0, 1.3, 1.6
Percent severe	Assumed to be severe (82).	PERT	0.95, 1.00, 1.00
Medical care seeking (severe)	Proportion (and 95% confidence interval (CI)) of survey respondents with bloody diarrhea who sought medical care from FoodNet Population Surveys (2000-2001, 2002-2003, 2006-2007) used as a proxy for severe illness (CDC, unpublished data).	PERT	Low, modal, high values: 0.19, 0.35, 0.51
Medical care seeking (mild)	Proportion (and 95% CI) of survey respondents with a non-bloody diarrhea who sought medical care from FoodNet Population Surveys (2000-2001, 2002-2003, 2006-2007) used as proxy for mild illness (CDC, unpublished data).	PERT	Low, modal, high values: 0.15, 0.18, 0.20
Specimen submission (severe)	Proportion (and 95% CI) of survey respondents who submitted a stool specimen among persons with bloody diarrhea who sought medical care from FoodNet Population Surveys (2000-2001, 2002-2003, 2006-2007) used as proxy for severe illness (CDC, unpublished data).	PERT	Low, modal, high values: 0.11, 0.36, 0.62
Specimen submission (mild)	Proportion (and 95% CI) of survey respondents who submitted a stool specimen among persons with a non-bloody diarrhea who sought medical care from FoodNet Population Surveys (2000-2001, 2002-2003, 2006-2007) used as a proxy for mild illness (CDC, unpublished data).	PERT	Low, modal, high values: 0.12, 0.19, 0.25
Laboratory testing	We assumed that most persons with *Trichinella* who submitted a specimen for testing would be tested for *Trichinella*.	PERT	Low, modal, high values: 0.95, 0.97, 1
Test sensitivity	Assumed to be 95% based on discussion with CDC experts.	PERT	Low, modal, high values: 0.93, 0.95, 0.97

Proportion hospitalized	Proportion from the CDC's Foodborne Disease Outbreak Surveillance System (2000–2007) (5). ¶No outbreaks reported in 2004 and 2007.	Empirical	Available years (2000-2007): 0.333, 0.286, 0.000, 0.000, ¶, 0.333, 0.500, ¶
Proportion who died	0.2% based on published study (83). Uncertainty with this proportion was based on a 50% relative increase/decrease from 0.0020 on an odds scale.	PERT	Low, modal, high values: 0.0013, 0.0020, 0.0030
Under-diagnosis (hospitalizations, deaths)	Number of hospitalizations and deaths doubled to account for under-diagnosis.	PERT	Low, modal, high values: 1, 2, 3
Proportion travel-related	3.7% based on surveillance data (81). Uncertainty with this proportion was based on a 50% relative increase/decrease from 0.037 on an odds scale.	PERT	Low, modal, high values: 0.025, 0.037, 0.054
Proportion foodborne	100% foodborne (84).	PERT	Low, modal, high values: 0.999, 1.000, 1.000

**Pathogen: *Vibrio cholerae,* toxigenic**			
**Model input**	**Data source(s)**	**Distribution**	**Distribution values**
Reported illnesses	Number of illnesses due to toxigenic *Vibrio cholerae* infection reported to CDC's Cholera and Other Vibrio Illness Surveillance (COVIS) System (2000-2007) (85). Because of an apparent trend over time, the empirical distribution was based on the predicted count for 2006 plus empirical residuals derived from a linear regression of the number of illnesses on year (see online Technical Appendix 2).	Empirical	By year (2000-2007): 7, 3, 2, 2, 5, 12, 8, 7
Population adjustment (year)	Population ratios applied to each year from 2000-2007 based on US Census population estimates (1).	Degenerate	Ratios by year (2000-2007): 1.058, 1.047, 1.038, 1.029, 1.019, 1.010, 1.000, 0.990
Underreporting	Passive surveillance multiplier used to adjust for underreporting (see online Technical Appendix 4).	PERT	Low, modal, high values: 0.9, 1.1, 1.3
Percent severe	Almost all cases assumed to be severe.	PERT	Low, modal, high values: 0.95, 1.00, 1.00
Medical care seeking (severe)	Proportion (and 95% confidence interval (CI)) of survey respondents with bloody diarrhea who sought medical care from FoodNet Population Surveys (2000-2001, 2002-2003, 2006-2007) used as a proxy for severe illness (CDC, unpublished data)	PERT	Low, modal, high values: 0.19, 0.35, 0.51

Medical care seeking (mild)	Proportion (and 95% CI) of survey respondents with a non-bloody diarrhea who sought medical care from FoodNet Population Surveys (2000-2001, 2002-2003, 2006-2007) used as proxy for mild illness (CDC, unpublished data).	PERT	Low, modal, high values: 0.15, 0.18, 0.20
Specimen submission (severe)	Proportion (and 95% CI) of survey respondents who submitted a stool specimen among persons with bloody diarrhea who sought medical care from FoodNet Population Surveys (2000-2001, 2002-2003, 2006-2007) used as proxy for severe illness (CDC, unpublished data).	PERT	Low, modal, high values: 0.11, 0.36, 0.62
Specimen submission (mild)	Proportion (and 95% CI) of survey respondents who submitted a stool specimen among persons with a non-bloody diarrhea who sought medical care from FoodNet Population Surveys (2000-2001, 2002-2003, 2006-2007) used as a proxy for mild illness (CDC, unpublished data).	PERT	Low, modal, high values: 0.12, 0.19, 0.25
Laboratory testing	We assumed that most persons with toxigenic *Vibrio cholerae* who submitted a specimen for testing would be tested.	PERT	Low, modal, high values: 0.95, 0.97, 1
Test sensitivity	Proportion of clinical laboratories using appropriate diagnostic tests to test stool samples for *Vibrio* spp. the FoodNet Laboratory Survey (12).	PERT	Low, modal, high values: 0.21, 0.28, 0.37
Proportion hospitalized	Proportion of cases of toxigenic *Vibrio cholerae* infection reported to COVIS who were hospitalized (2000-2007).	Empirical	By year (2000-2007): 0.571, 0.333, 0.000, 0.500, 0.400, 0.417, 0.500, 0.714
Proportion who died	Proportion of cases of toxigenic *Vibrio cholerae* infection reported to COVIS who died (2000-2007).	Empirical	By year (2000-2007): 0.000, 0.000, 0.000, 0.000, 0.000, 0.000, 0.000, 0.000
Under-diagnosis (hospitalizations, deaths)	Number of hospitalizations and deaths doubled to account for under-diagnosis.	PERT	Low, modal, high values: 1, 2, 3
Proportion travel-related	Based on proportion of cases of toxigenic *Vibrio cholerae* infection reported to COVIS who acquired the infection while traveling outside the United States (2000-2007).	PERT	Low, modal, high values: 0.42, 0.69, 1.00
Proportion foodborne	Based on proportion of cases of toxigenic *Vibrio cholerae* infection reported to COVIS that were classified as foodborne (2000-2007).	PERT	Low, modal, high values: 0.999, 1.000, 1.000

**Pathogen:** ***Vibrio vulnificus***			
**Model input**	**Data source(s)**	**Distribution**	**Distribution values**
Reported illnesses	Number of illnesses due to *Vibrio vulnificus* infection reported to CDC's Cholera and Other Vibrio Illness Surveillance (COVIS) System (2000-2007) (85). Because of an apparent trend over time, the empirical distribution was based on the predicted count for 2006 plus empirical residuals derived from a linear regression of the number of illnesses on year (see online Technical Appendix 2).	Empirical	By year (2000-2007):<br>67, 89, 89, 119, 125 123, 100, 98
Population adjustment (year)	Population ratios applied to each year from 2000-2007 based on US Census population estimates (1).	Degenerate	Ratios by year (2000-2007):<br>1.058, 1.047, 1.038, 1.029, 1.019, 1.010, 1.000, 0.990
Underreporting	Passive surveillance multiplier used to adjust for underreporting (see online Technical Appendix 4).	PERT	Low, modal, high values:<br>0.9, 1.1, 1.3
Percent severe	Almost all cases assumed to be severe.	PERT	Low, modal, high values:<br>0.95, 1, 1
Medical care seeking (severe)	Assumed to have a high rate of medical care seeking.	PERT	Low, modal, high values:<br>0.80, 0.90, 1.00
Medical care seeking (mild)	Assumed to have a high rate of medical care seeking.	PERT	Low, modal, high values:<br>0.80, 0.90, 1.00
Specimen submission (severe)	Assumed to have a high rate of specimen submission.	PERT	Low, modal, high values:<br>0.70, 0.80, 0.90
Specimen submission (mild)	Assumed to have a high rate of specimen submission.	PERT	Low, modal, high values:<br>0.70, 0.80, 0.90
Laboratory testing	We assumed that most persons with *Vibrio vulnificus* who submitted a specimen for testing would be tested.	PERT	Low, modal, high values:<br>0.94, 0.97, 1.00
Test sensitivity	Based on sensitivity of blood cultures (86, 87).	PERT	Low, modal, high values:<br>0.70, 0.85, 1.00
Proportion hospitalized	Proportion of cases of *Vibrio vulnificus* infection reported to COVIS who were hospitalized (2000-2007).	Empirical	By year (2000-2007):<br>0.983, 0.905, 0.907, 0.936, 0.886, 0.895, 0.862, 0.926
Proportion who died	Proportion of cases of *Vibrio vulnificus* infection reported to COVIS who died (2000-2007).	Empirical	By year (2000-2007):<br>0.377, 0.360, 0.402, 0.308, 0.360, 0.253, 0.360, 0.369

Under-diagnosis (hospitalizations, deaths)	Number of hospitalizations and deaths double to account for under-diagnosis.	PERT	Low, modal, high values: 1, 2, 3
Proportion travel-related	Based on proportion of cases of *Vibrio vulnificus* infection reported to COVIS who acquired the infection while traveling outside the United States (2000-2007).	PERT	Low, modal, high values: 0, 0.02, 0.03
Proportion foodborne	Based on proportion of cases of *Vibrio vulnificus* infection reported to COVIS that were classified as foodborne (2000-2007).	PERT	Low, modal, high values: 0.31, 0.48, 0.60

**Pathogen: *Vibrio parahaemolyticus***			
**Model input**	**Data source(s)**	**Distribution**	**Distribution values**
Reported illnesses	Number of illnesses due to *Vibrio parahaemolyticus* infection reported to CDC's Cholera and Other Vibrio Illness Surveillance (COVIS) System (2000-2007) (85). Because of an apparent trend over time, the empirical distribution was based on the predicted count for 2006 plus empirical residuals derived from a linear regression of the number of illnesses on year (see online Technical Appendix 2).	Empirical	By year (2000-2007): 139, 155, 156, 170, 276, 219, 408, 239
Population adjustment (year)	Population ratios applied to each year from 2000-2007 based on US Census population estimates (1).	Degenerate	Ratios by year (2000-2007): 1.058, 1.047, 1.038, 1.029, 1.019, 1.010, 1.000, 0.990
Underreporting	Passive surveillance multiplier used to adjust for underreporting (see online Technical Appendix 4).	PERT	Low, modal, high values: 0.9, 1.1, 1.3
Percent severe	Assumed to be a similar illness to non-typhoidal *Salmonella* infection.	PERT	Low, modal, high values: 0.35, 0.45, 0.71
Medical care seeking (severe)	Proportion (and 95% confidence interval (CI)) of survey respondents with bloody diarrhea who sought medical care from FoodNet Population Surveys (2000-2001, 2002-2003, 2006-2007) used as a proxy for severe illness (CDC, unpublished data).	PERT	Low, modal, high values: 0.19, 0.35, 0.51
Medical care seeking (mild)	Proportion (and 95% CI) of survey respondents with a non-bloody diarrhea who sought medical care from FoodNet Population Surveys (2000-2001, 2002-2003, 2006-2007) used as proxy for mild illness (CDC, unpublished data).	PERT	Low, modal, high values: 0.15, 0.18, 0.20

Specimen submission (severe)	Proportion (and 95% CI) of survey respondents who submitted a stool specimen among persons with bloody diarrhea who sought medical care from FoodNet Population Surveys (2000-2001, 2002-2003, 2006-2007) used as proxy for severe illness (CDC, unpublished data).	PERT	Low, modal, high values: 0.11, 0.36, 0.62
Specimen submission (mild)	Proportion (and 95% CI) of survey respondents who submitted a stool specimen among persons with a non-bloody diarrhea who sought medical care from FoodNet Population Surveys (2000-2001, 2002-2003, 2006-2007) used as a proxy for mild illness (CDC, unpublished data).	PERT	Low, modal, high values: 0.12, 0.19, 0.25
Laboratory testing	Proportion of clinical laboratories routinely testing stool samples for *Vibrio* spp. from the FoodNet Laboratory Survey (12).	PERT	Low, modal, high values: 0.41, 0.51, 0.61
Test sensitivity	Proportion of clinical laboratories using appropriate diagnostic tests to test stool samples for *Vibrio* spp. the FoodNet Laboratory Survey (12).	PERT	Low, modal, high values: 0.21, 0.28, 0.37
Proportion hospitalized	Proportion of cases of *Vibrio parahaemolyticus* infection reported to COVIS who were hospitalized (2000-2007).	Empirical	By year (2000-2007): 0.205, 0.254, 0.275, 0.182, 0.241, 0.230, 0.178, 0.238
Proportion who died	Proportion of cases of *Vibrio parahaemolyticus* infection reported to COVIS who died (2000-2007).	Empirical	By year (2000-2007): 0.007, 0.000, 0.037, 0.007, 0.012, 0.010, 0.003, 0.000
Under-diagnosis (hospitalizations, deaths)	Number of hospitalizations and deaths double to account for under-diagnosis.	PERT	Low, modal, high values: 1, 2, 3
Proportion travel-related	Based on proportion of cases of *Vibrio. parahaemolyticus* infection reported to COVIS who acquired the infection while traveling outside the United States (2000-2007).	PERT	Low, modal, high values: 0.08, 0.10, 0.14
Proportion foodborne	Based on proportion of cases of *Vibrio parahaemolyticus* infection reported to COVIS that were classified as foodborne (2000-2007).	PERT	Low, modal, high values: 0.76, 0.87, 0.92

**Pathogen: *Vibrio* spp., other**			
**Model input**	**Data source(s)**	**Distribution**	**Distribution values**
Reported illnesses	Number of illnesses due to *Vibrio spp.* other than toxigenic *V. cholerae*, *V. vulnificus*, and *V. parahaemolyticus* reported to CDC's Cholera and Other Vibrio Illness Surveillance (COVIS) System (2000-2007) (85). Because of an apparent trend over time, the empirical distribution was based on the predicted count for 2006 plus empirical residuals derived from a linear regression of the number of illnesses on year (see online Technical Appendix 2).	Empirical	By year (2000-2007): 98, 132, 208, 201, 179, 209, 227, 218
Population adjustment (year)	Population ratios applied to each year from 2000-2007 based on US Census population estimates (1).	Degenerate	Ratios by year (2000-2007): 1.058, 1.047, 1.038, 1.029, 1.019, 1.010, 1.000, 0.990
Underreporting	Passive surveillance multiplier used to adjust for underreporting (see online Technical Appendix 4).	PERT	Low, modal, high values: 0.9, 1.1, 1.3
Percent severe	Assumed to be a similar illness to non-typhoidal *Salmonella* infection.	PERT	Low, modal, high values: 0.35, 0.45, 0.71
Medical care seeking (severe)	Proportion (and 95% confidence interval (CI)) of survey respondents with bloody diarrhea who sought medical care from FoodNet Population Surveys (2000-2001, 2002-2003, 2006-2007) used as a proxy for severe illness (CDC, unpublished data).	PERT	Low, modal, high values: 0.19, 0.35, 0.51
Medical care seeking (mild)	Proportion (and 95% CI) of survey respondents with a non-bloody diarrhea who sought medical care from FoodNet Population Surveys (2000-2001, 2002-2003, 2006-2007) used as proxy for mild illness (CDC, unpublished data).	PERT	Low, modal, high values: 0.15, 0.18, 0.20
Specimen submission (severe)	Proportion (and 95% CI) of survey respondents who submitted a stool specimen among persons with bloody diarrhea who sought medical care from FoodNet Population Surveys (2000-2001, 2002-2003, 2006-2007) used as proxy for severe illness (CDC, unpublished data).	PERT	Low, modal, high values: 0.10, 0.36, 0.62

Specimen submission (mild)	Proportion (and 95% CI) of survey respondents who submitted a stool specimen among persons with a non-bloody diarrhea who sought medical care from FoodNet Population Surveys (2000-2001, 2002-2003, 2006-2007) used as a proxy for mild illness (CDC, unpublished data).	PERT	Low, modal, high values: 0.12, 0.19, 0.25
Laboratory testing	Proportion of clinical laboratories routinely testing stool samples for *Vibrio spp.* from the FoodNet Laboratory Survey (12).	PERT	Low, modal, high values: 0.41, 0.51, 0.61
Test sensitivity	Proportion of clinical laboratories using appropriate diagnostic tests to test stool samples for *Vibrio* spp. the FoodNet Laboratory Survey (12).	PERT	Low, modal, high values: 0.21, 0.28, 0.37
Proportion hospitalized	Proportion of cases of *Vibrio*, other infection reported to COVIS who were hospitalized (2000-2007).	Empirical	By year (2000-2007): 0.375, 0.359, 0.369, 0.396, 0.353, 0.437, 0.361, 0.317
Proportion who died	Proportion of cases of *Vibrio*, other infection reported to COVIS who died (2000-2007).	Empirical	By year (2000-2007): 0.021, 0.015, 0.037, 0.068, 0.035, 0.069, 0.020, 0.032
Under-diagnosis (hospitalizations, deaths)	Number of hospitalizations and deaths double to account for under-diagnosis.	PERT	Low, modal, high values: 1, 2, 3
Proportion travel-related	Based on proportion of cases of *Vibrio*, other infection reported to COVIS who acquired the infection while traveling outside the United States (2000-2007).	PERT	Low, modal, high values: 0.06, 0.11, 0.17
Proportion foodborne	Based on proportion of cases of *Vibrio*, other infection reported to COVIS that were classified as foodborne (2000-2007).	PERT	Low, modal, high values: 0.48, 0.57, 0.67

**Pathogen: *Yersinia enterocolitica***			
**Model input**	**Data source(s)**	**Distribution**	**Parameters**
Reported illnesses	Incidence of Y*ersinia enterocolitica* infection reported to CDC's Foodborne Diseases Active Surveillance Network (FoodNet) by FoodNet site (n=10) and year (2005-2008) (10).	Empirical	By site and year (2005-2008) see Tables 3.1 and 3.2 in this online Technical Appendix
Population adjustment (year)	Incidence of *Yersinia enterocolitica* in each FoodNet site by year applied to 2006 US Census population estimates (1).	Degenerate	Adjustment by year (2005-2008): 1.010, 1.000, 0.990, 0.981
Underreporting	No underreporting multiplier, we assumed all laboratory-confirmed *Yersinia enterocolitica* illnesses were enumerated by FoodNet active surveillance.	-	-
Proportion severe	Proportion of cases of *Yersinia enterocolitica* infection with bloody diarrhea from FoodNet study in two sites (88). Uncertainty with this proportion (9%) was based on a 50% relative increase/decrease from 0.09 on an odds scale.	PERT	Low, modal, high values: 0.062, 0.09, 0.129
Medical care seeking (severe)	Proportion (and 95% confidence interval (CI)) of survey respondents with bloody diarrhea who sought medical care from FoodNet Population Surveys (2000-2001, 2002-2003, 2006-2007) (CDC, unpublished data).	PERT	Low, modal, high values: 0.19, 0.35, 0.51
Medical care seeking (mild)	Proportion (and 95% CI) of survey respondents with a non-bloody diarrhea who sought medical care from FoodNet Population Surveys (2000-2001, 2002-2003, 2006-2007) (CDC, unpublished data)	PERT	Low, modal, high values: 0.15, 0.18, 0.20
Specimen submission (severe)	Proportion (and 95% CI) of survey respondents who submitted a stool specimen among persons with bloody diarrhea who sought medical care from FoodNet Population Surveys (2000-2001, 2002-2003, 2006-2007) (CDC, unpublished data).	PERT	Low, modal, high values: 0.11, 0.36, 0.62
Specimen submission (mild)	Proportion (and 95% CI) of survey respondents who submitted a stool specimen among persons with a non-bloody diarrhea who sought medical care from FoodNet Population Surveys (2000-1, 2002-3, 2006-7) (CDC, unpublished data).	PERT	Low, modal, high values: 0.12, 0.19, 0.25
Laboratory testing	Proportion of clinical laboratories routinely testing stool samples for *Yersinia enterocolitica* from the FoodNet Laboratory Survey (12).	PERT	Low, modal, high values: 0.31, 0.40, 0.50

Test sensitivity	Proportion of clinical laboratories using appropriate diagnostic tests to test stool samples for *Yersinia enterocolitica* the FoodNet Laboratory Survey (12).	PERT	Low, modal, high values: 0.49, 0.59, 0.68
Proportion hospitalized	Proportion of FoodNet cases of *Yersinia enterocolitica* infection hospitalized (2005-2008).	Empirical	By site and year (2005-2008) see Table 3.3 in this online Technical Appendix
Proportion who died	Proportion of FoodNet cases of *Yersinia enterocolitica* infection who died (2005-2008).	Empirical	By site and year (2005-2008) see Table 3.4 in this online Technical Appendix
Under-diagnosis (hospitalizations, deaths)	Number of hospitalizations and deaths doubled to account for under-diagnosis.	PERT	Low, modal, high values: 1, 2, 3
Proportion travel-related	Proportion of FoodNet cases of *Yersinia enterocolitica* infection who reported travel outside the United States within 7 days of illness onset (2005-2008). Uncertainty with this proportion (7%) was based on a 50% relative increase/decrease from 0.07 on an odds scale.	PERT	Low, modal, high values: 0.05, 0.07, 0.10
Proportion foodborne	From a published review (89). Uncertainty with this proportion (90%) was based on a 50% relative increase/decrease from 0.90 on an odds scale.	PERT	Low, modal, high values: 0.80, 0.90, 1.00

Table 3.1: Number of cases of illness reported to CDC's Foodborne Diseases Active Surveillance Network (FoodNet) by pathogen, year, and FoodNet site

Pathogen	Year	FoodNet site									
		**CA**	**CO**	**CT**	**GA**	**MD**	**MN**	**NM**	**NY**	**OR**	**TN**
Campylobacter spp.	2005	920	495	543	585	403	843	352	507	641	403
Campylobacter spp.	2006	866	479	532	581	432	899	383	522	634	443
Campylobacter spp.	2007	923	421	493	689	414	907	350	522	705	448
Campylobacter spp.	2008	985	388	530	683	378	884	357	479	690	480
Cryptosporidium spp.	2005	48	25	84	154	32	166	17	708	48	45
Cryptosporidium spp.	2006	47	37	38	276	20	242	41	54	77	47
Cryptosporidium spp.	2007	40	102	42	231	33	302	120	89	129	137
Cryptosporidium spp.	2008	43	27	41	258	55	235	174	114	58	47
Cyclospora cayetanensis	2005	2	0	35	13	3	0	4	1	4	3
Cyclospora cayetanensis	2006	0	0	11	19	2	4	1	0	2	4
Cyclospora cayetanensis	2007	1	0	3	3	1	0	2	2	0	1
Cyclospora cayetanensis	2008	0	0	4	2	3	3	2	0	0	3
E. coli O157 STEC	2005	28	26	43	33	27	121	10	74	66	45
E. coli O157 STEC	2006	42	35	41	41	40	147	20	53	83	88
E. coli O157 STEC	2007	39	32	45	47	22	165	10	58	73	56
E. coli O157 STEC	2008	37	82	26	44	33	120	15	51	56	54
E. coli non-O157 STEC	2005	5	4	20	8	24	35	11	11	8	2
E. coli non-O157 STEC	2006	6	16	34	18	47	44	23	19	9	11
E. coli non-O157 STEC	2007	9	55	26	40	35	41	23	12	5	24
E. coli non-O157 STEC	2008	1	23	17	27	49	53	27	16	5	10
Listeria monocytogenes	2005	10	2	20	22	19	15	4	17	11	10
Listeria monocytogenes	2006	8	5	18	17	28	5	5	19	11	9
Listeria monocytogenes	2007	7	9	11	31	14	6	4	10	8	14
Listeria monocytogenes	2008	19	4	14	21	14	5	5	16	5	12
Salmonella, non-typhoidal*	2005	453	336	460	1920	779	573	251	488	372	813
Salmonella, non-typhoidal*	2006	469	353	502	1836	768	720	258	493	397	841
Salmonella, non-typhoidal*	2007	469	311	423	2030	854	702	283	520	317	850
Salmonella, non-typhoidal*	2008	460	335	491	2276	836	748	516	431	395	905

Salmonella serotype Typhi	2005	14	5	8	8	12	6	1	0	4	3
Salmonella serotype Typhi	2006	17	6	4	5	8	5	1	2	4	1
Salmonella serotype Typhi	2007	9	6	8	17	16	9	0	1	3	1
Salmonella serotype Typhi	2008	17	2	3	9	17	7	2	2	2	4
Shigella spp.	2005	283	101	58	668	99	96	133	66	85	506
Shigella spp.	2006	244	180	67	1375	128	259	172	48	94	198
Shigella spp.	2007	188	79	44	1638	109	237	107	38	66	363
Shigella spp.	2008	159	85	40	1103	117	311	154	33	74	968
Yersinia enterocolitica	2005	28	7	15	28	4	17	2	20	9	18
Yersinia enterocolitica	2006	10	5	16	32	9	22	5	12	12	29
Yersinia enterocolitica	2007	14	4	17	42	8	19	3	14	16	13
Yersinia enterocolitica	2008	10	7	14	42	14	14	2	15	12	21

*In all analyses in this paper, serotype Paratyphi is grouped with non-typhoidal S*almonella*.

Table 3.2: US cases of illness projected from CDC's Foodborne Diseases Active Surveillance Network (FoodNet) by pathogen, year, and FoodNet site

Pathogen	Year	FoodNet site									
		**CA**	**CO**	**CT**	**GA**	**MD**	**MN**	**NM**	**NY**	**OR**	**TN**
Campylobacter spp.	2005	85289	57079	46572	19193	21566	49270	54903	35342	52803	20096
Campylobacter spp.	2006	79857	54239	45509	18602	23007	52153	58967	36470	51389	21781
Campylobacter spp.	2007	84281	46720	42149	21586	21983	52219	53160	36526	56309	21738
Campylobacter spp.	2008	88706	42161	45250	21039	20019	50581	53678	33519	54319	23092
Cryptosporidium spp.	2005	4450	2883	7205	5053	1712	9702	2652	49353	3954	2244
Cryptosporidium spp.	2006	4334	4190	3251	8837	1065	14039	6312	3773	6241	2311
Cryptosporidium spp.	2007	3652	11319	3591	7237	1752	17387	18226	6228	10303	6647
Cryptosporidium spp.	2008	3872	2934	3494	7948	2913	13431	26162	7977	4566	2256
Cyclospora cayetanensis	2005	185	0	3002	427	161	0	624	70	330	150
Cyclospora cayetanensis	2006	0	0	941	608	107	232	154	0	162	197
Cyclospora cayetanensis	2007	91	0	256	94	53	0	304	140	0	49
Cyclospora cayetanensis	2008	0	0	341	62	159	171	301	0	0	144

E. coli O157 STEC	2005	2596	2998	3688	1083	1445	7072	1560	5158	5437	2244
E. coli O157 STEC	2006	3873	3963	3507	1313	2130	8528	3079	3703	6728	4327
E. coli O157 STEC	2007	3561	3551	3847	1473	1168	9500	1519	4058	5831	2717
E. coli O157 STEC	2008	3332	8910	2216	1355	1748	6858	2255	3569	4408	2592
E. coli non-O157 STEC	2005	464	461	1715	262	1284	2046	1716	767	659	100
E. coli non-O157 STEC	2006	553	1812	2908	576	2503	2553	3541	1327	730	541
E. coli non-O157 STEC	2007	822	6104	2223	1253	1858	2360	3493	840	399	1165
E. coli non-O157 STEC	2008	90	2499	1449	832	2595	3029	4060	1120	394	480
Listeria monocytogenes	2005	927	231	1715	722	1017	877	624	1185	906	499
Listeria monocytogenes	2006	738	566	1540	544	1491	290	770	1327	892	443
Listeria monocytogenes	2007	639	999	940	971	743	345	608	700	639	679
Listeria monocytogenes	2008	1711	435	1193	647	741	286	752	1120	394	576
Salmonella, non-typhoidal	2005	41995	38744	39453	62993	41686	33490	39150	34017	30644	40542
Salmonella, non-typhoidal	2006	43248	39972	42942	58784	40902	41769	39722	34444	32179	41350
Salmonella, non-typhoidal	2007	42825	34513	36164	63599	45347	40416	42983	36386	25319	41243
Salmonella, non-typhoidal	2008	41426	36402	41841	70111	44276	42751	77585	30160	31095	43447
Salmonella serotype Typhi	2005	1298	577	686	262	642	351	156	0	330	150
Salmonella serotype Typhi	2006	1568	679	342	160	426	290	154	140	324	49
Salmonella serotype Typhi	2007	822	666	684	533	850	518	0	70	240	49
Salmonella serotype Typhi	2008	1531	217	256	277	900	400	301	140	157	192
Shigella spp.	2005	26236	11646	4975	21916	5298	5611	20745	4601	7002	25233
Shigella spp.	2006	22500	20382	5731	44024	6817	15025	26481	3354	7619	9735
Shigella spp.	2007	17167	8767	3762	51318	5788	13645	16252	2659	5272	17613
Shigella spp.	2008	14319	9236	3409	33977	6196	17775	23155	2309	5825	46472
Yersinia enterocolitica	2005	2596	807	1287	919	214	994	312	1394	741	898
Yersinia enterocolitica	2006	922	566	1369	1025	479	1276	770	838	973	1426
Yersinia enterocolitica	2007	1278	444	1453	1316	425	1094	456	980	1278	631
Yersinia enterocolitica	2008	901	761	1193	1294	741	800	301	1050	945	1008

Table 3.3: Proportion of case-patients hospitalized from CDC's Foodborne Diseases Active Surveillance Network (FoodNet) by pathogen, year, and FoodNet site

Pathogen	Year	FoodNet site									
		CA	CO	CT	GA	MD	MN	NM	NY	OR	TN
Campylobacter spp.	2005	0.1060	0.1087	0.2016	0.2363	0.2141	0.1295	0.1502	0.1584	0.0939	0.2575
Campylobacter spp.	2006	0.1256	0.1006	0.1909	0.2226	0.5370	0.1316	0.1485	0.1862	0.0738	0.2126
Campylobacter spp.	2007	0.1292	0.1041	0.2056	0.1872	0.1959	0.1287	0.2060	0.1437	0.0893	0.2426
Campylobacter spp.	2008	0.1196	0.1111	0.1589	0.1969	0.2235	0.1357	0.1813	0.1809	0.0762	0.2500
Cryptosporidium spp.	2005	0.2444	0.1600	0.1449	0.3650	0.5667	0.2108	0.1875	0.0395	0.1042	0.2750
Cryptosporidium spp.	2006	0.3810	0.1081	0.2143	0.2918	0.8462	0.1777	0.2500	0.1667	0.1739	0.3721
Cryptosporidium spp.	2007	0.2105	0.1237	0.2059	0.3258	0.5161	0.1739	0.1875	0.1591	0.0569	0.1308
Cryptosporidium spp.	2008	0.3030	0.4231	0.1622	0.3347	0.6038	0.1923	0.1557	0.1150	0.0714	0.2750
Cyclospora cayetanensis	2005	0.5000		0.0286	0.0000	0.0000		0.0000	0.0000	0.0000	0.0000
Cyclospora cayetanensis	2006			0.1000	0.0000	1.0000	0.0000	0.0000		0.0000	0.0000
Cyclospora cayetanensis	2007	0.0000		0.0000	0.0000			0.0000	0.0000		0.0000
Cyclospora cayetanensis	2008			0.0000	0.0000	0.0000	0.0000	0.0000			0.0000
E. coli O157 STEC	2005	0.4444	0.3846	0.4419	0.4848	0.2800	0.3802	0.1000	0.4384	0.3636	0.6364
E. coli O157 STEC	2006	0.4524	0.4000	0.4390	0.5610	0.9500	0.4150	0.5500	0.4717	0.5060	0.6023
E. coli O157 STEC	2007	0.4872	0.3438	0.3778	0.6170	0.5909	0.4242	0.3333	0.5000	0.3836	0.5636
E. coli O157 STEC	2008	0.3784	0.3537	0.4231	0.6364	0.4545	0.2750	0.5333	0.5400	0.4107	0.5660
E. coli non-O157 STEC	2005	0.0000	0.0000	0.0500	0.2500	0.1818	0.0286	0.5000	0.2727	0.0000	0.0000
E. coli non-O157 STEC	2006	0.0000	0.0000	0.2941	0.1111	0.2000	0.0909	0.1667	0.2105	0.0000	0.2727
E. coli non-O157 STEC	2007	0.0000	0.0727	0.1667	0.0256	0.1176	0.2500	0.1364	0.3333	0.2000	0.1739
E. coli non-O157 STEC	2008	0.0000	0.0000	0.1176	0.0417	0.0208	0.2075	0.0000	0.1250	0.2000	0.3000
Listeria monocytogenes	2005	1.0000	1.0000	0.9500	0.9091	1.0000	1.0000	0.7500	0.8824	0.9091	0.8000
Listeria monocytogenes	2006	0.8750	1.0000	1.0000	0.8824	1.0000	1.0000	1.0000	0.9474	0.7273	0.8889
Listeria monocytogenes	2007	1.0000	0.8889	1.0000	0.9355	0.8571	1.0000	1.0000	1.0000	0.8750	0.8571
Listeria monocytogenes	2008	0.8947	1.0000	0.9286	0.9048	1.0000	1.0000	1.0000	1.0000	1.0000	0.9167
Salmonella, non-typhoidal	2005	0.2151	0.1918	0.2857	0.3440	0.2993	0.2483	0.2712	0.2531	0.1425	0.3980
Salmonella, non-typhoidal	2006	0.2299	0.2087	0.2612	0.3037	0.8221	0.1986	0.2705	0.3063	0.1924	0.3729
Salmonella, non-typhoidal	2007	0.2153	0.1836	0.2597	0.2877	0.2973	0.2325	0.2481	0.2521	0.2050	0.3297
Salmonella, non-typhoidal	2008	0.2342	0.2018	0.2112	0.2797	0.3125	0.2735	0.2414	0.2430	0.2127	0.3360

Salmonella serotype Typhi	2005	0.7692	0.8000	0.7143	0.5000	0.8182	0.8333	0.0000		0.5000	1.0000
Salmonella serotype Typhi	2006	0.6875	0.6667	0.7500	1.0000	0.8750	1.0000	1.0000	1.0000	0.5000	1.0000
Salmonella serotype Typhi	2007	0.6250	0.6667	0.7500	0.3529	0.7500	0.6667			0.6667	1.0000
Salmonella serotype Typhi	2008	0.6429	1.0000	0.3333	1.0000	0.7647	1.0000	0.5000	1.0000	1.0000	1.0000
Shigella spp.	2005	0.1526	0.2222	0.1429	0.2496	0.2143	0.1771	0.2177	0.1667	0.1882	0.1982
Shigella spp.	2006	0.1391	0.1173	0.3019	0.1883	0.6042	0.1550	0.2160	0.2292	0.1915	0.2515
Shigella spp.	2007	0.1453	0.1429	0.2069	0.1214	0.2233	0.1838	0.1837	0.1842	0.1667	0.1753
Shigella spp.	2008	0.1644	0.1786	0.2105	0.1668	0.2818	0.2058	0.2414	0.2121	0.1918	0.1448
Yersinia enterocolitica	2005	0.2500	0.4286	0.4615	0.4074	0.6667	0.2941	0.5000	0.4500	0.2222	0.5625
Yersinia enterocolitica	2006	0.5556	0.4000	0.3077	0.6250	0.4000	0.1818	0.5000	0.3333	0.1667	0.5185
Yersinia enterocolitica	2007	0.1667	0.0000	0.1667	0.4048	0.5000	0.2105	0.5000	0.1429	0.2667	0.3077
Yersinia enterocolitica	2008	0.6000	0.1429	0.4286	0.3846	0.3846	0.2143	0.0000	0.2667	0.2727	0.1500

Table 3.4: Proportion of case-patients who died from CDC's Foodborne Diseases Active Surveillance Network (FoodNet) by pathogen, year, and FoodNet site

**Pathogen**	**Year**	**FoodNet site**									
		**CA**	**CO**	**CT**	**GA**	**MD**	**MN**	**NM**	**NY**	**OR**	**TN**
Campylobacter spp.	2005	0.0000	0.0000	0.0000	0.0000	0.0000	0.0000	0.0000	0.0000	0.0000	0.0000
Campylobacter spp.	2006	0.0000	0.0000	0.0027	0.0046	0.0000	0.0011	0.0028	0.0000	0.0000	0.0000
Campylobacter spp.	2007	0.0041	0.0000	0.0026	0.0035	0.0000	0.0011	0.0000	0.0000	0.0014	0.0022
Campylobacter spp.	2008	0.0065	0.0000	0.0038	0.0018	0.0027	0.0011	0.0000	0.0063	0.0014	0.0021
Cryptosporidium spp.	2005	0.0000	0.0000	0.0000	0.0088	0.0000	0.0060	0.0000	0.0000	0.0000	0.0000
Cryptosporidium spp.	2006	0.0250	0.0000	0.0000	0.0093	0.0000	0.0000	0.0000	0.0000	0.0000	0.0238
Cryptosporidium spp.	2007	0.0000	0.0000	0.0000	0.0152	0.0000	0.0066	0.0000	0.0000	0.0000	0.0000
Cryptosporidium spp.	2008	0.0000	0.0000	0.0000	0.0372	0.0000	0.0043	0.0000	0.0000	0.0000	0.0000
Cyclospora cayetanensis	2005	0.0000		0.0000	0.0000	0.0000		0.0000	0.0000	0.0000	0.0000
Cyclospora cayetanensis	2006			0.0000	0.0000	0.0000	0.0000	0.0000		0.0000	0.0000
Cyclospora cayetanensis	2007	0.0000		0.0000	0.0000			0.0000	0.0000		0.0000
Cyclospora cayetanensis	2008			0.0000	0.0000	0.0000	0.0000	0.0000			0.0000

E. coli O157 STEC	2005	0.0370	0.0000	0.0000	0.0000	0.0000	0.0000	0.0000	0.0000	0.0000	0.0270
E. coli O157 STEC	2006	0.0000	0.0000	0.0000	0.0000	0.0250	0.0000	0.0000	0.0000	0.0000	0.0000
E. coli O157 STEC	2007	0.0000	0.0000	0.0000	0.0000	0.0000	0.0000	0.0000	0.0172	0.0000	0.0000
E. coli O157 STEC	2008	0.0000	0.0122	0.0000	0.0256	0.0000	0.0000	0.0000	0.0196	0.0179	0.0189
E. coli non-O157 STEC	2005	0.0000	0.0000	0.0000	0.1250	0.0000	0.0000	0.0000	0.0000	0.0000	0.0000
E. coli non-O157 STEC	2006	0.0000	0.0000	0.0000	0.0000	0.0000	0.0000	0.0000	0.0000	0.0000	0.0000
E. coli non-O157 STEC	2007	0.0000	0.0000	0.0000	0.0000	0.0000	0.0000	0.0000	0.0000	0.0000	0.0000
E. coli non-O157 STEC	2008	0.0000	0.0000	0.0000	0.0000	0.0000	0.0000	0.0000	0.0000	0.0000	0.0000
Listeria monocytogenes	2005	0.4000	0.0000	0.1500	0.0000	0.1111	0.1333	0.0000	0.1765	0.1818	0.0000
Listeria monocytogenes	2006	0.0000	0.0000	0.0556	0.0588	0.0741	0.2000	0.4000	0.1579	0.3636	0.0000
Listeria monocytogenes	2007	0.1429	0.0000	0.3636	0.0968	0.1538	0.3333	0.2500	0.1000	0.1250	0.2857
Listeria monocytogenes	2008	0.1579	0.0000	0.4286	0.1429	0.1429	0.2000	0.4000	0.1875	0.2000	0.1818
Salmonella, non-typhoidal	2005	0.0074	0.0030	0.0055	0.0053	0.0039	0.0017	0.0130	0.0061	0.0000	0.0081
Salmonella, non-typhoidal	2006	0.0024	0.0028	0.0071	0.0092	0.0028	0.0014	0.0285	0.0061	0.0000	0.0026
Salmonella, non-typhoidal	2007	0.0141	0.0000	0.0116	0.0041	0.0024	0.0014	0.0074	0.0038	0.0032	0.0012
Salmonella, non-typhoidal	2008	0.0026	0.0030	0.0021	0.0050	0.0086	0.0107	0.0040	0.0000	0.0076	0.0011
Salmonella serotype Typhi	2005	0.0000	0.0000	0.0000	0.0000	0.0000	0.0000	0.0000		0.0000	0.0000
Salmonella serotype Typhi	2006	0.0000	0.0000	0.0000	0.0000	0.0000	0.0000	0.0000	0.0000	0.0000	0.0000
Salmonella serotype Typhi	2007	0.0000	0.0000	0.0000	0.0000	0.0000	0.0000		0.0000	0.0000	0.0000
Salmonella serotype Typhi	2008	0.0000	0.0000	0.0000	0.0000	0.0000	0.0000	0.0000	0.0000	0.0000	0.0000
Shigella spp.	2005	0.0000	0.0000	0.0000	0.0024	0.0103	0.0000	0.0085	0.0000	0.0000	0.0000
Shigella spp.	2006	0.0067	0.0056	0.0000	0.0011	0.0000	0.0000	0.0000	0.0000	0.0000	0.0000
Shigella spp.	2007	0.0000	0.0000	0.0000	0.0008	0.0000	0.0000	0.0000	0.0000	0.0152	0.0000
Shigella spp.	2008	0.0000	0.0000	0.0000	0.0011	0.0000	0.0000	0.0000	0.0000	0.0000	0.0000
Yersinia enterocolitica	2005	0.0000	0.0000	0.0000	0.0000	0.0000	0.0000	0.0000	0.0000	0.0000	0.0714
Yersinia enterocolitica	2006	0.1111	0.0000	0.0000	0.0870	0.0000	0.0000	0.2500	0.0833	0.0833	0.0000
Yersinia enterocolitica	2007	0.0000	0.0000	0.0000	0.0000	0.0000	0.0000	0.0000	0.0000	0.0000	0.0000
Yersinia enterocolitica	2008	0.1000	0.0000	0.0000	0.0000	0.0000	0.0000	0.0000	0.0000	0.0000	0.0000

Table 3.5 Underreporting and under-diagnosis multipliers for the 25 known pathogens with surveillance data available

**Pathogen**	**Underreporting multiplier†**<br>**Mean (90% credible interval [CrI])**	**Under-diagnosis multiplier‡**<br>**Mean (90% CrI)**
Bacillus cereus	25.5 (8.7-52.3)	29.3 (21.8-38.5)
Brucella spp.	1.1 (1.0-1.2)	15.1 (10.7-21.4)
Campylobacter spp.	-	30.3 (23.2-39.0)
Clostridium botulinum, foodborne	1.1 (1.0-1.2)	2.0 (1.8-2.3)
Clostridium perfringens	25.5 (8.7-52.3)	29.3 (21.8-38.5)
Cryptosporidium spp.	-	98.6 (73.5-130.3)
Cyclospora cayetanensis	-	83.1 (59.1-114.5)
E. coli, enterotoxigenic (ETEC)	25.5 (8.7-52.3)	29.3 (21.8-38.4)
E. coli, Shiga toxin–producing (STEC) non-O157	-	106.8 (75.4-146.3)
E. coli, Shiga toxin–producing (STEC) O157	-	26.1 (16.1-41.3)
Giardia intestinalis	1.3 (1.1-1.5)	46.3 (36.0-59.3)
Hepatitis A	1.1 (1.0-1.2)	9.1 (6.9-11.8)
Listeria monocytogenes	-	2.3 (2.0-2.6)
Mycobacterium bovis	-	1.1 (1.0-1.1)
Salmonella serotype Typhi	-	13.3 (7.6-22.8)
Salmonella, non-typhoidal	-	29.3 (21.8-38.4)
Shigella spp.	-	33.3 (25.1-43.4)
Staphylococcus aureus, foodborne	25.5 (8.7-52.3)	29.3 (21.8-38.5)
Streptococcus spp., Group A, foodborne	25.5 (8.7-52.3)	29.3 (21.8-38.5)
Trichinella spp.	1.3 (1.1-1.5)	9.8 (5.6-16.7)
Vibrio cholerae, toxigenic	1.1 (1.0-1.2)	33.1 (18.4-57.5)
Vibrio parahaemolyticus	1.1 (1.0-1.2)	142.4 (100.1-195.1)
Vibrio spp., other	1.1 (1.0-1.2)	142.7 (100.4-195.2)
Vibrio vulnificus	1.1 (1.0-1.2)	1.7 (1.5-2.0)
Yersinia enterocolitica	-	122.8 (91.2-163.0)

†Adjustment for underreporting due to surveillance method; underreporting multiplier for passive surveillance systems (COVIS and NNDSS) derived by comparing the incidence of laboratory-confirmed illnesses for *Listeria*, non-typhoidal *Salmonella*, *Shigella*, and STEC O157 (for bacteria) and *Cryptosporidium* spp. and *Cyclospora cayetanensis* (for parasites) ascertained in FoodNet to the incidence of laboratory-confirmed illnesses for the same pathogens reportable to NNDSS; underreporting multiplier for outbreak-associated illness reported through the Foodborne Disease Outbreak Surveillance System derived by comparing the incidence of laboratory-confirmed illnesses caused by *Listeria*, non-typhoidal *Salmonella*, *Shigella*, and STEC O157 ascertained in FoodNet to the incidence of laboratory-confirmed illnesses of these bacterial infections reported to FDOSS. More detail on the data used to estimate underreporting multipliers is given in online Technical Appendix 4.
‡ Adjustment for under-diagnosis due to variations in medical care seeking, specimen submission, laboratory testing, and test sensitivity.

References

1. US Census Bureau. Population Estimates. [cited 2010 March 9]; Available from: http://www.census.gov/popest/states/NST-ann-est.html.
2. Widdowson, M.A., Meltzer, M.I., Zhang, X., Bresee, J.S., Parashar, U.D., and Glass, R.I. Cost-effectiveness and potential impact of rotavirus vaccination in the United States. Pediatrics. 2007;119:684-97.
3. Dennehy, P.H., Nelson, S.M., Spangenberger, S., Noel, J.S., Monroe, S.S., and Glass, R.I. A prospective case-control study of the role of astrovirus in acute diarrhea among hospitalized young children. J Infect Dis. 2001;184:10-5.
4. Glass, R.I., Noel, J., Mitchell, D., Herrmann, J.E., Blacklow, N.R., Pickering, L.K., et al. The changing epidemiology of astrovirus-associated gastroenteritis: a review. Arch Virol Suppl. 1996;12:287-300.
5. Centers for Disease Control and Prevention. Surveillance for foodborne disease outbreaks - United States, 2006. MMWR Morb Mortal Wkly Rep. 2009;58:609-15.
6. Nieves, E., Jajosky, R.A., Adams, D.A., Sharp, P., Anderson, W.J., Aponte, J.J., et al. Summary of notifiable diseases--United States, 2007. MMWR Morb Mortal Wkly Rep. 2009;56:1-94.
7. American Society for Microbiology. in Manual for Clinical Microbiology, vol 1. 2003, ASM Press: Washington, DC.
8. Taylor, J.P. and Perdue, J.N. The changing epidemiology of human brucellosis in Texas, 1977-1986. Am J Epidemiol. 1989;130:160-5.
9. Chomel, B.B., DeBess, E.E., Mangiamele, D.M., Reilly, K.F., Farver, T.B., Sun, R.K., et al. Changing trends in the epidemiology of human brucellosis in California from 1973 to 1992: a shift toward foodborne transmission. J Infect Dis. 1994;170:1216-23.
10. Centers for Disease Control and Prevention. Preliminary FoodNet Data on the incidence of infection with pathogens transmitted commonly through food--10 States, 2008. MMWR Morb Mortal Wkly Rep. 2009;58:333-7.
11. Friedman, C.R., Hoekstra, R.M., Samuel, M., Marcus, R., Bender, J., Shiferaw, B., et al. Risk factors for sporadic *Campylobacter* infection in the United States: A case-control study in FoodNet sites. Clin Infect Dis. 2004;38 Suppl 3:S285-96.
12. Voetsch, A.C., Angulo, F.J., Rabatsky-Ehr, T., Shallow, S., Cassidy, M., Thomas, S.M., et al. Laboratory practices for stool-specimen culture for bacterial pathogens, including Escherichia coli O157:H7, in the FoodNet sites, 1995-2000. Clin Infect Dis. 2004;38 Suppl 3:S190-7.
13. Chalker, R.B. and Blaser, M.J. A review of human salmonellosis: III. Magnitude of Salmonella infection in the United States. Rev Infect Dis. 1988;10:111-24.
14. Voetsch, A.C., Van Gilder, T.J., Angulo, F.J., Farley, M.M., Shallow, S., Marcus, R., et al. FoodNet estimate of the burden of illness caused by nontyphoidal Salmonella infections in the United States. Clin Infect Dis. 2004;38 Suppl 3:S127-34.

15. St Louis, M.E., Peck, S.H., Bowering, D., Morgan, G.B., Blatherwick, J., Banerjee, S., et al. Botulism from chopped garlic: delayed recognition of a major outbreak. Ann Intern Med. 1988;108:363-8.

16. Woodruff, B.A., Griffin, P.M., McCroskey, L.M., Smart, J.F., Wainwright, R.B., Bryant, R.G., et al. Clinical and laboratory comparison of botulism from toxin types A, B, and E in the United States, 1975-1988. J Infect Dis. 1992;166:1281-6.

17. Huang, D.B. and White, A.C. An updated review on *Cryptosporidium* and *Giardia*. Gastroenterol Clin North Am. 2006;35:291-314.

18. Jones, J.L., Lopez, A., Wahlquist, S.P., Nadle, J., and Wilson, M. Survey of clinical laboratory practices for parasitic diseases. Clin Infect Dis. 2004;38 Suppl 3:S198-202.

19. Alles, A.J., Waldron, M.A., Sierra, L.S., and Mattia, A.R. Prospective comparison of direct immunofluorescence and conventional staining methods for detection of Giardia and Cryptosporidium spp. in human fecal specimens. J Clin Microbiol. 1995;33:1632-4.

20. Garcia, L.S., Shum, A.C., and Bruckner, D.A. Evaluation of a new monoclonal antibody combination reagent for direct fluorescence detection of Giardia cysts and Cryptosporidium oocysts in human fecal specimens. J Clin Microbiol. 1992;30:3255-7.

21. Garcia, L.S. and Shimizu, R.Y. Evaluation of nine immunoassay kits (enzyme immunoassay and direct fluorescence) for detection of Giardia lamblia and Cryptosporidium parvum in human fecal specimens. J Clin Microbiol. 1997;35:1526-9.

22. Kehl, K.S., Cicirello, H., and Havens, P.L. Comparison of four different methods for detection of *Cryptosporidium* species. J Clin Microbiol. 1995;33:416-8.

23. Majowicz, S.E., Michel, P., Aramini, J.J., McEwen, S.A., and Wilson, J.B. Descriptive analysis of endemic cryptosporidiosis cases reported in Ontario, 1996-1997. Can J Public Health. 2001;92:62-6.

24. Visvesvara, G.S., Moura, H., Kovacs-Nace, E., Wallace, S., and Eberhard, M.L. Uniform staining of Cyclospora oocysts in fecal smears by a modified safranin technique with microwave heating. J Clin Microbiol. 1997;35:730-3.

25. Berlin, O.G., Peter, J.B., Gagne, C., Conteas, C.N., and Ash, L.R. Autofluorescence and the detection of cyclospora oocysts. Emerg Infect Dis. 1998;4:127-8.

26. Lopez, B., De Merida, A.M., Arrowood, M.J., Bern, C., Hernandez, D.B., Del Rio, D., et al. Comparison of Two Microscopic Diagnostic Methods for Cyclospora cayetanensis. in International Conference on Emerging Infectious Diseases. 1998. Atlanta, GA.

27. Verweij, J.J., Laeijendecker, D., Brienen, E.A., van Lieshout, L., and Polderman, A.M. Detection of Cyclospora cayetanensis in travellers returning from the tropics and subtropics using microscopy and real-time PCR. Int J Med Microbiol. 2003;293:199-202.

28. Blans, M.C., Ridwan, B.U., Verweij, J.J., Rozenberg-Arska, M., and Verhoef, J. Cyclosporiasis outbreak, Indonesia. Emerg Infect Dis. 2005;11:1453-5.

29. Herwaldt, B.L. *Cyclospora cayetanensis*: a review, focusing on the outbreaks of cyclosporiasis in the 1990s. Clin Infect Dis. 2000;31:1040-57.

30. Herwaldt, B. The ongoing saga of U.S. outbreaks of cyclosporiasis associated with imported fresh produce: what *Cyclospora cayetanensis* has taught us and what we have yet to learn, in Addressing foodborne threats to health: policies, practices, and global coordination. Coordination Workshop Summary, B.o.G.H. Forum on Microbial Threats, Institute of Medicine of the National Academies, Editor. 2006, The National Academies Press: Washington, DC. 85-115, 33-40

31. Voetsch, A.C., Kennedy, M.H., Keene, W.E., Smith, K.E., Rabatsky-Ehr, T., Zansky, S., et al. Risk factors for sporadic Shiga toxin-producing *Escherichia coli* O157 infections in FoodNet sites, 1999-2000. Epidemiol Infect. 2007;135:993-1000.

32. Rangel, J.M., Sparling, P.H., Crowe, C., Griffin, P.M., and Swerdlow, D.L. Epidemiology of *Escherichia coli* O157:H7 outbreaks, United States, 1982-2002. Emerg Infect Dis. 2005;11:603-9.

33. Hedican, E.B., Medus, C., Besser, J.M., Juni, B.A., Koziol, B., Taylor, C., et al. Characteristics of O157 versus non-O157 Shiga toxin-producing *Escherichia coli* infections in Minnesota, 2000-2006. Clin Infect Dis. 2009;49:358-64.

34. Manning, S.D., Madera, R.T., Schneider, W., Dietrich, S.E., Khalife, W., Brown, W., et al. Surveillance for Shiga toxin-producing *Escherichia coli*, Michigan, 2001-2005. Emerg Infect Dis. 2007;13:318-21.

35. Centers for Disease Control and Prevention. Laboratory-confirmed non-O157 Shiga toxin-producing *Escherichia coli*--Connecticut, 2000-2005. MMWR Morb Mortal Wkly Rep. 2007;56:29-31.

36. Centers for Disease Control and Prevention. Non-O157 Shiga toxin-producing E. coli (STEC) outbreaks, United States. Memorandum to the Record. 2010.

37. Mead, P.S., Slutsker, L., Dietz, V., McCaig, L.F., Bresee, J.S., Shapiro, C., et al. Food-related illness and death in the United States. Emerg Infect Dis. 1999;5:607-25.

38. Thomson, R.B., Jr., Haas, R.A., and Thompson, J.H., Jr. Intestinal parasites: the necessity of examining multiple stool specimens. Mayo Clin Proc. 1984;59:641-2.

39. Rosoff, J.D., Sanders, C.A., Sonnad, S.S., De Lay, P.R., Hadley, W.K., Vincenzi, F.F., et al. Stool diagnosis of giardiasis using a commercially available enzyme immunoassay to detect Giardia-specific antigen 65 (GSA 65). J Clin Microbiol. 1989;27:1997-2002.

40. Scheffler, E.H. and Van Etta, L.L. Evaluation of rapid commercial enzyme immunoassay for detection of Giardia lamblia in formalin-preserved stool specimens. J Clin Microbiol. 1994;32:1807-8.

41. Hiatt, R.A., Markell, E.K., and Ng, E. How many stool examinations are necessary to detect pathogenic intestinal protozoa? Am J Trop Med Hyg. 1995;53:36-9.

42. Zimmerman, S.K. and Needham, C.A. Comparison of conventional stool concentration and preserved-smear methods with Merifluor Cryptosporidium/Giardia Direct Immunofluorescence Assay and ProSpecT Giardia EZ Microplate Assay for detection of Giardia lamblia. J Clin Microbiol. 1995;33:1942-3.

43. Cartwright, C.P. Utility of multiple-stool-specimen ova and parasite examinations in a high-prevalence setting. J Clin Microbiol. 1999;37:2408-11.

44. Hanson, K.L. and Cartwright, C.P. Use of an enzyme immunoassay does not eliminate the need to analyze multiple stool specimens for sensitive detection of Giardia lamblia. J Clin Microbiol. 2001;39:474-7.

45. Johnston, S.P., Ballard, M.M., Beach, M.J., Causer, L., and Wilkins, P.P. Evaluation of three commercial assays for detection of *Giardia* and *Cryptosporidium* organisms in fecal specimens. J Clin Microbiol. 2003;41:623-6.

46. Healthcare Cost and Utilization Project. National Inpatient Sample. [cited 2010 February 8]; Available from: http://www.hcup-us.ahrq.gov/nisoverview.jsp.

47. Chute, C.G., Smith, R.P., and Baron, J.A. Risk factors for endemic giardiasis. Am J Public Health. 1987;77:585-7.

48. Daniels, D., Grytdal, S., and Wasley, A. Surveillance for acute viral hepatitis - United States, 2007. MMWR Surveill Summ. 2009;58:1-27.

49. Heron, M., Hoyert, D.L., Murphy, S.L., Xu, J., Kochanek, K.D., and Tejada-Vera, B. Deaths: Final Data for 2006. National Vital Statistics Reports; vol 57 no 14. National Center for Health Statistics. 2009: Hyattsville, Maryland.

50. National Center for Health Statistics. Multiple Cause-of-Death Public-Use Data Files. [cited 2010 February 8]; Available from: http://www.cdc.gov/nchs/products/elec_prods/subject/mortmcd.htm.

51. Klevens, M., Miller, J., Iqbal, K., Thomas, A., Rocchio, E., Hanson, H., et al. The evolving epidemiology of Hepatitis A in the United States: Incidence and molecular epidemiology from population-based surveillance, 2005-2007. Arch Intern Med. In press.

52. Mylonakis, E., Hohmann, E.L., and Calderwood, S.B. Central nervous system infection with Listeria monocytogenes. 33 years' experience at a general hospital and review of 776 episodes from the literature. Medicine (Baltimore). 1998;77:313-36.

53. Schwartz, B., Ciesielski, C.A., Broome, C.V., Gaventa, S., Brown, G.R., Gellin, B.G., et al. Association of sporadic listeriosis with consumption of uncooked hot dogs and undercooked chicken. Lancet. 1988;2:779-82.

54. Pinner, R.W., Schuchat, A., Swaminathan, B., Hayes, P.S., Deaver, K.A., Weaver, R.E., et al. Role of foods in sporadic listeriosis. II. Microbiologic and epidemiologic investigation. The Listeria Study Group. JAMA. 1992;267:2046-50.

55. Riedo, F.X., Pinner, R.W., Tosca, M.L., Cartter, M.L., Graves, L.M., Reeves, M.W., et al. A point-source foodborne listeriosis outbreak: documented incubation period and possible mild illness. J Infect Dis. 1994;170:693-6.

56. Schuchat, A., Deaver, K.A., Wenger, J.D., Plikaytis, B.D., Mascola, L., Pinner, R.W., et al. Role of foods in sporadic listeriosis. I. Case-control study of dietary risk factors. The Listeria Study Group. JAMA. 1992;267:2041-5.

57. Varma, J.K., Samuel, M.C., Marcus, R., Hoekstra, R.M., Medus, C., Segler, S., et al. *Listeria monocytogenes* infection from foods prepared in a commercial establishment: a case-control study of potential sources of sporadic illness in the United States. Clin Infect Dis. 2007;44:521-8.

58. de Wit, M.A., Koopmans, M.P., Kortbeek, L.M., Wannet, W.J., Vinje, J., van Leusden, F., et al. Sensor, a population-based cohort study on gastroenteritis in the Netherlands: incidence and etiology. Am J Epidemiol. 2001;154:666-74.

59. Centers for Disease Control and Prevention. Reported Tuberculosis in the United States, 2007. 2008, Department of Health and Human Services, CDC: Atlanta, GA.

60. Hlavsa, M.C., Moonan, P.K., Cowan, L.S., Navin, T.R., Kammerer, J.S., Morlock, G.P., et al. Human tuberculosis due to *Mycobacterium bovis* in the United States, 1995-2005. Clin Infect Dis. 2008;47:168-75.

61. Taylor, Z., Marks, S.M., Rios Burrows, N.M., Weis, S.E., Stricof, R.L., and Miller, B. Causes and costs of hospitalization of tuberculosis patients in the United States. Int J Tuberc Lung Dis. 2000;4:931-9.

62. Centers for Disease Control and Prevention. Human tuberculosis caused by *Mycobacterium bovis*--New York City, 2001-2004. MMWR Morb Mortal Wkly Rep. 2005;54:605-8.

63. Wheeler, J.G., Sethi, D., Cowden, J.M., Wall, P.G., Rodrigues, L.C., Tompkins, D.S., et al. Study of infectious intestinal disease in England: rates in the community, presenting to general practice, and reported to national surveillance. The Infectious Intestinal Disease Study Executive. BMJ. 1999;318:1046-50.

64. Amar, C.F., East, C.L., Gray, J., Iturriza-Gomara, M., Maclure, E.A., and McLauchlin, J. Detection by PCR of eight groups of enteric pathogens in 4,627 faecal samples: re-examination of the English case-control Infectious Intestinal Disease Study (1993-1996). Eur J Clin Microbiol Infect Dis. 2007;26:311-23.

65. Marshall, J.A., Hellard, M.E., Sinclair, M.I., Fairley, C.K., Cox, B.J., Catton, M.G., et al. Incidence and characteristics of endemic Norwalk-like virus-associated gastroenteritis. J Med Virol. 2003;69:568-78.

66. Patel, M.M., Widdowson, M.A., Glass, R.I., Akazawa, K., Vinje, J., and Parashar, U.D. Systematic literature review of role of noroviruses in sporadic gastroenteritis. Emerg Infect Dis. 2008;14:1224-31.

67. Jansen, A., Stark, K., Kunkel, J., Schreier, E., Ignatius, R., Liesenfeld, O., et al. Aetiology of community-acquired, acute gastroenteritis in hospitalised adults: a prospective cohort study. BMC Infect Dis. 2008;8:143.

68. National Center for Health Statistics. National Hospital Discharge Survey. [cited 2010 February 2]; Available from: http://www.cdc.gov/nchs/nhds.htm.

69. National Center for Health Statistics. Ambulatory Health Care Data. [cited 2010 February 8]; Available from: http://www.cdc.gov/nchs/ahcd/about_ahcd.htm.

70. Kimura, A.C., Reddy, V., Marcus, R., Cieslak, P.R., Mohle-Boetani, J.C., Kassenborg, H.D., et al. Chicken consumption is a newly identified risk factor for sporadic *Salmonella enterica* serotype Enteritidis infections in the United States: a case-control study in FoodNet sites. Clin Infect Dis. 2004;38 Suppl 3:S244-52.

71. Hennessy, T.W., Cheng, L.H., Kassenborg, H., Ahuja, S.D., Mohle-Boetani, J., Marcus, R., et al. Egg consumption is the principal risk factor for sporadic Salmonella serotype Heidelberg infections: a case-control study in FoodNet sites. Clin Infect Dis. 2004;38 Suppl 3:S237-43.

72. Mermin, J., Hutwagner, L., Vugia, D., Shallow, S., Daily, P., Bender, J., et al. Reptiles, amphibians, and human *Salmonella* infection: a population-based, case-control study. Clin Infect Dis. 2004;38 Suppl 3:S253-61.

73. Marcus, R., Varma, J.K., Medus, C., Boothe, E.J., Anderson, B.J., Crume, T., et al. Re-assessment of risk factors for sporadic *Salmonella* serotype Enteritidis infections: a case-control study in five FoodNet Sites, 2002-2003. Epidemiol Infect. 2007;135:84-92.

74. Parry, C.M., Hien, T.T., Dougan, G., White, N.J., and Farrar, J.J. Typhoid fever. N Engl J Med. 2002;347:1770-82.

75. Olsen, S.J., Bleasdale, S.C., Magnano, A.R., Landrigan, C., Holland, B.H., Tauxe, R.V., et al. Outbreaks of typhoid fever in the United States, 1960-99. Epidemiol Infect. 2003;130:13-21.

76. Haley, C.C., Ong, K.L., Hedberg, K., Cieslak, P.R., Scallan, E., Marcus, R., et al. Risk factors for sporadic shigellosis, FoodNet 2005. Foodborne Pathog Dis. 2010;7:741-7.

77. Jones, J.L., Kruszon-Moran, D., Sanders-Lewis, K., and Wilson, M. *Toxoplasma gondii* infection in the United States, 1999-2004, decline from the prior decade. Am J Trop Med Hyg. 2007;77:405-10.

78. World Health Organization. Toxoplasmosis, in Technical Report Series, No. 431. 1969, World Health Organization: Geneva.

79. Lopez, A., Dietz, V.J., Wilson, M., Navin, T.R., and Jones, J.L. Preventing congenital toxoplasmosis. MMWR Recomm Rep. 2000;49:59-68.

80. Cook, A.J., Gilbert, R.E., Buffolano, W., Zufferey, J., Petersen, E., Jenum, P.A., et al. Sources of toxoplasma infection in pregnant women: European multicentre case-control study. European Research Network on Congenital Toxoplasmosis. BMJ. 2000;321:142-7.

81. Kennedy, E.D., Hall, R.L., Montgomery, S.P., Pyburn, D.G., and Jones, J.L. Trichinellosis surveillance - United States, 2002-2007. MMWR Surveill Summ. 2009;58:1-7.

82. McAuley, J.B., Michelson, M.K., Hightower, A.W., Engeran, S., Wintermeyer, L.A., and Schantz, P.M. A trichinosis outbreak among Southeast Asian refugees. Am J Epidemiol. 1992;135:1404-10.

83. Hennekeuser, H.H., Pabst, K., Poeplau, W., and Gerok, W. Zur Klinik und Therapie der Trichinose: Boebachtungen and 47 Patienten wahrend einer Epidemic. Disch Med Wochenschr 1968;93:867-73.

84. Capo, V. and Despommier, D.D. Clinical aspects of infection with *Trichinella* spp. Clin Microbiol Rev. 1996;9:47-54.

85. Centers for Disease Control and Prevention. Cholera and Other Vibrio Illness Surveillance System. [cited 2010 Jul 27]; Available from: http://www.cdc.gov/nationalsurveillance/cholera_vibrio_surveillance.html.

86. Cockerill, F.R., 3rd, Wilson, J.W., Vetter, E.A., Goodman, K.M., Torgerson, C.A., Harmsen, W.S., et al. Optimal testing parameters for blood cultures. Clin Infect Dis. 2004;38:1724-30.

87. Lee, A., Mirrett, S., Reller, L.B., and Weinstein, M.P. Detection of bloodstream infections in adults: how many blood cultures are needed? J Clin Microbiol. 2007;45:3546-8.

88. Townes, J.M., Deodhar, A.A., Laine, E.S., Smith, K., Krug, H.E., Barkhuizen, A., et al. Reactive arthritis following culture-confirmed infections with bacterial enteric pathogens in Minnesota and Oregon: a population-based study. Ann Rheum Dis. 2008;67:1689-96.

89. Ostroff, S. *Yersinia* as an emerging infection: epidemiologic aspects of yersiniosis. Contrib Microbiol Immunol. 1995;13:5-10.
