# Supplementary material for: Foodborne Illness Acquired in the United States—Major Pathogens
Source: Emerg Infect Dis. 2011 Jan;17(1):7–15. doi: 10.3201/eid1701.P11101 (PMC3375761; doi:10.3201/eid1701.P11101)
Supplement: Appendix — Data Used to Estimate Passive and Outbreak Surveillance Underreporting Multipliers. [file 09-1101p1-Techapp4.pdf]

Article DOI: 10.3201/eid1701.P11101

Foodborne Illness Acquired in the United States—Major Pathogens

Technical Appendix 4

Data Used to Estimate Passive and Outbreak Surveillance Underreporting Multipliers

Passive surveillance underreporting multipliers

To estimate the total number of illnesses due to the 9 (of 31) known pathogens with passive surveillance data available from the National Notifiable Disease Surveillance System (NNDSS) and the Cholera and Other *Vibrio* Illness Surveillance System (COVIS) (Box 1), we applied a passive underreporting multiplier to correct for the underreporting of cases. That is, we scaled reported counts of cases to estimated numbers had they been reported through active surveillance.

Box 1: Pathogens with passive surveillance case counts
- *Brucella* spp.
- *Clostridium botulinum*
- *Giardia intestinalis*
- Hepatitis A
- *Trichinella* spp.
- *Vibrio cholerae,* toxigenic
- *Vibrio parahaemolyticus*
- *Vibrio vulnificus*
- *Vibrio* spp., other

The approach taken was that of simple ratio estimation. We assumed that all laboratory-confirmed illnesses were enumerated by FoodNet active surveillance and applied observed ratios from pathogens in FoodNet for which we also had passive NNDSS surveillance case counts. (Box 2). That is, we computed ratios of projected total laboratory-confirmed case counts obtained through active surveillance of FoodNet pathogens to passive surveillance case counts for those pathogens in NNDSS. We then examined the distributions of these numbers. Note that FoodNet does receive counts of laboratory-confirmed illnesses for *Vibrio* spp.; however, we

chose not to use ratios of FoodNet to COVIS case counts to estimate underreporting because of the complex association of *Vibrio* spp. infections with coastal areas.

Box 2: Pathogens with both active and passive surveillance case counts

- *Cryptosporidium* spp.
- *Cyclospora cayetanensis*
- *E. coli* O157, Shiga toxin–producing (STEC) O157
- *Listeria monocytogenes*
- *Salmonella* spp.
- *Shigella* spp.

Based on these empirical distributions we extracted sets of summary features, to create a general description of pathogen-to-pathogen variability in active surveillance to passive surveillance case count ratios. Based on differences in reporting practices, we expected to treat bacterial and parasitic pathogens separately. We then used these features to inform PERT probability distributions of ratios. These PERT distributions were the source of the multipliers that were then applied to the pathogens for which we used passive surveillance data from NNDSS and COVIS to estimate total illnesses.

The observed active to passive surveillance pathogen ratios are shown in Table 1. Note that the table rows do not exactly match the classifications used for FoodNet pathogens in estimating burden of illness. This reflects features of NNDSS surveillance. FoodNet *Salmonella* data has been collapsed. FoodNet *E. coli* data has been split into two classifications: *E. coli* O157 (STEC) for 2000-2006 data and *E. coli* O157 (STEC) combined with *E. coli* non-O157 (STEC) for 2007-2008 data. The table includes four columns of summary measures applied to the individual pathogen annual ratios: mean annual ratio, group means of means for parasites and for bacteria, median annual ratio, and group mean of medians for parasites and for bacteria. The variety of summarizations is motivated by the annual data, displayed in Figure 1. The figure suggests that parasitic and bacterial pathogens should indeed be treated differently, and that is what we chose to do. Based on the data as presented in Table 1 and Figure 1, in addition to subjective inputs from authors on surveillance issues surrounding bacterial and parasitic pathogens we chose PERT distributions as follows:

Bacterial: low=0.9, modal=1.1, high=1.3

Parasitic: low=1.0, modal= 1.3, high=1.6

The PERT variance parameter was fixed at its default value of 4.

Table 1: Active and passive surveillance pathogen case counts and ratios

Pathogen	Group	Number of Years	Sum of FoodNet Projected US Illnesses	Sum of NNDSS Reported US illnesses	Mean Annual Ratio	Group Mean of Means	Median Annual Ratio	Group Mean of Medians
Cryptosporidium spp.	Parasitic	8	43,364	39,912	1.09	1.19	1.20	1.39
Cyclospora cayetanensis	Parasitic	8	1,782	1,382	1.29	1.19	1.58	1.39
E. coli (STEC)	Bacterial	2	10,736	9,279	1.16	1.10	1.16	1.08
E. coli O157 (STEC)	Bacterial	6	23,870	19,491	1.22	1.10	1.23	1.08
Listeria monocytogenes	Bacterial	8	6,837	6,070	1.13	1.10	1.08	1.08
Salmonella spp.	Bacterial	8	345,557	349,312	0.99	1.10	0.98	1.08
Shigella spp.	Bacterial	8	157,667	156,321	1.01	1.10	0.95	1.08

Figure 1: Annual active to passive surveillance ratios by pathogen. Boxplots are overlaid for visual comparison.

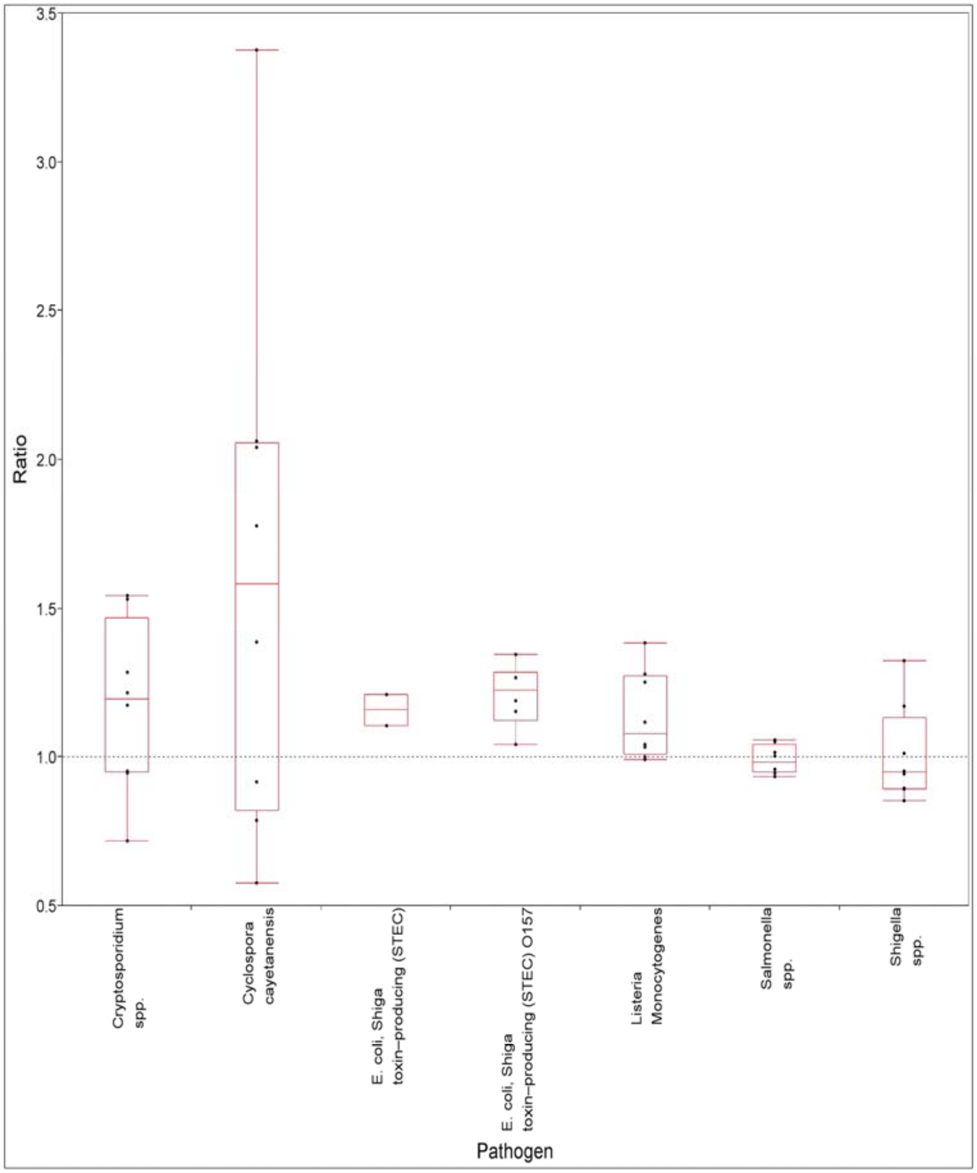

Outbreak surveillance underreporting multipliers

To estimate the total number of illnesses due to the 5 (of 31) pathogens with only outbreak data available from the Foodborne Disease Outbreak Surveillance System (FDOSS) (Box 3), we applied an outbreak underreporting multiplier to scale reported counts of outbreak-related cases to projected counts of national laboratory-confirmed illness.

Box 3: Pathogens with only outbreak-related case counts

- *Bacillus cereus*
- *Clostridium perfringens*
- *E. coli*, enterotoxigenic (ETEC)
- *Staphylococcus aureus*
- *Streptococcus* spp., Group A

The approach taken was again that of simple ratio estimation. We computed ratios of total laboratory-confirmed case counts in FoodNet active surveillance to outbreak-associated laboratory-confirmed case counts in FoodNet active surveillance (Box 4). In this use of FoodNet data, the outbreak-related cases are a subset of the total, obtained by exhaustive review. We used 2004-2008 data because of its completeness over the period. We then examined the distribution of these numbers.

Box 4: Pathogens with both active surveillance case counts and outbreak-related case counts

- *Campylobacter* spp.
- *Cryptosporidium* spp.
- *Cyclospora cayetanensis*
- *E. coli*, Shiga toxin–producing (STEC), O157
- *E. coli*, Shiga toxin–producing (STEC), non-O157
- *Listeria monocytogenes*
- *Salmonella*, non-typhoidal
- *Salmonella* serotype Typhi
- *Shigella* spp.
- *Vibrio* spp.
- *Yersinia entercolitica*

Based on this empirical distribution, we extracted a set of summary features to create a general description of pathogen-to-pathogen variability in active surveillance to outbreak case count ratios. We then used those features to inform a PERT probability distribution of ratios. This PERT distribution was the source of the multipliers that were then applied to the pathogens for which we only had outbreak data. Note that in contrast to the passive surveillance multipliers we chose not to distinguish bacterial and parasitic pathogens. This was done because, while the observed parasitic ratios tended to be smaller than the bacterial ratios, we did not find an

epidemiological or surveillance argument for distinguishing them and the sample size was small. Further, the set of pathogens to which they would be applied was diverse.

The data available to us with both outbreak and laboratory-confirmed case counts was FoodNet data. We assumed that these data produced ratios that were representative of ratios that would be obtained under national surveillance in 2006. We also assumed that pathogens for which we had only outbreak data could be reasonably adjusted using a single multiplier distribution. That is, we did not attempt to estimate a specific multiplier for each of the 5 pathogens. Because of the fine granularity of the FoodNet data, we were able to consider ratios computed at multiple levels of aggregation. That is, we computed ratios of pathogen case counts at the overall level, but also at the level of year and at the level of FoodNet site. Finer aggregations produced too many cells with 0 outbreak cases to be useful. The year-level and site-level analyses produced observed ratios that were sufficiently homogeneous to suggest that our assumption that FoodNet ratios were applicable to national outbreak data (for the same pathogens) was reasonable. The extension to the 5 outbreak surveillance pathogens remains an untested assumption.

The observed FoodNet pathogen ratios are shown in Table 2. The data is strongly skewed toward higher numbers. Further, the four largest multipliers, for *Yersinia*, *Campylobacter*, *Salmonella* serotype Typhi, and *Listeria*, depend on small denominator values and/or derive from a small number of outbreaks. In light of this, did a range of analyses, seeking a highly robust summary. We computed multipliers for the data at different levels of aggregation including state by pathogen and year by pathogen levels. We then computed medians of the resulting multipliers across states and across years. The results were consistent; there was no evidence of substantive variation in ratio distribution by state or year. From the data one might argue that any value between, say, 10 and 75, could be advocated. The overall mean, that is, the total number of active surveillance cases divided by the total number of outbreak associated cases, is 18.4. Maximum likelihood fits of PERT distributions to the complete data and various subsets and variations of the data considered in sensitivity analyses yielded means of between 30 and 60. An ad hoc median of medians analysis yielded a value of 25.6. Given the uncertainties in modeling this adjustment factor, we chose this compromise value of 25.6 as the target mean of our multiplier distribution. We then chose to seek this target with a PERT distribution parameterized using 9 of the 11 FoodNet pathogen ratios; the two extreme ratios (*Listeria*, 381.0) and

Cyclospora, 4.6) were dropped. This trimming was motivated by concerns about the basis for the values of the top 4 pathogen multipliers and that the extreme values may contain additional sampling artifacts. We used the minimum (5), maximum (237), and median (16) of the 9 values to define the minimum, mode, and maximum parameters of the PERT distribution. The remaining PERT variance parameter was chosen to equal 20, producing a PERT distribution with mean equal to 25.5, essentially achieving our target value. It is possible to use the untrimmed data to create a PERT distribution with very similar characteristics, including a mean value of ~25, but we prefer to make our down-weighting of the extreme values explicit.

Table 2: Active and outbreak surveillance pathogen case counts and ratios

Pathogen	Total lab-confirmed cases	Outbreak-related lab-confirmed cases	Ratio
Yersinia entercolitica	762	2	381.0
Campylobacter spp.	28,878	122	236.7
Salmonella serotype Typhi	304	4	76.0
Listeria monocytogenes	651	9	72.3
Vibrio spp.	646	10	64.6
Salmonella, non-typhoidal	33,677	2,121	15.9
Shigella spp.	13,021	1,097	11.9
E. coli, Shiga toxin-producing (STEC) non-O157	963	90	10.7
Cryptosporidium spp.	5,120	767	6.7
E. coli, Shiga toxin-producing (STEC) O157	2,530	470	5.4
Cyclospora cayetanensis	153	33	4.6
**Total**	**86,705**	**4,725**	**18.4**
